# Using realist review to inform intervention development: methodological illustration and conceptual platform for collaborative care in offender mental health

**DOI:** 10.1186/s13012-015-0321-2

**Published:** 2015-09-28

**Authors:** M. Pearson, S. L Brand, C. Quinn, J. Shaw, M. Maguire, S. Michie, S. Briscoe, C. Lennox, A. Stirzaker, T. Kirkpatrick, R. Byng

**Affiliations:** Collaboration for Leadership in Applied Health Research and Care (CLAHRC) for the South West Peninsula, University of Exeter Medical School, South Cloisters, St Luke’s Campus, Exeter, EX1 2LU UK; Centre for Clinical Trials and Health Research, Plymouth University Peninsula Schools of Medicine and Dentistry, ITTC Building, Davy Road, Plymouth Science Park, Plymouth, PL6 8BX UK; Institute of Brain, Behaviour and Mental Health, Jean McFarlane Building, University of Manchester, Oxford Road, Manchester, M13 9PL UK; Centre for Criminology, University of South Wales, Pontypridd, CF37 1DL UK; UCL Centre for Behaviour Change, University College London, 1-19 Torrington Place, London, WC1E 7HB UK; Avon & Wiltshire Mental Health Partnership Trust, Old Town Surgery, Curie Avenue, Swindon, SN1 4GB UK

## Abstract

**Background:**

This paper reports how we used a realist review, as part of a wider project to improve collaborative mental health care for prisoners with common mental health problems, to develop a conceptual platform. The importance of offenders gaining support for their mental health, and the need for practitioners across the health service, the criminal justice system, and the third sector to work together to achieve this is recognised internationally. However, the literature does not provide coherent analyses of *how* these ambitions can be achieved. This paper demonstrates how a realist review can be applied to inform complex intervention development that spans different locations, organisations, professions, and care sectors.

**Methods:**

We applied and developed a realist review for the purposes of intervention development, using a three-stage process. (1) An iterative database search strategy (extending beyond criminal justice and offender health) and groups of academics, practitioners, and people with lived experience were used to identify explanatory accounts (*n* = 347). (2) From these accounts, we developed *consolidated* explanatory accounts (*n* = 75). (3) The identified interactions between practitioners and offenders (within their organisational, social, and cultural contexts) were specified in a conceptual platform. We also specify, step by step, how these explanatory accounts were documented, consolidated, and built into a conceptual platform. This addresses an important methodological gap for social scientists and intervention developers about how to develop and articulate programme and implementation theory underpinning complex interventions.

**Results:**

An integrated person-centred system is proposed to improve collaborative mental health care for offenders with common mental health problems (near to and after release) by achieving consistency between the goals of different sectors and practitioners, enabling practitioners to apply scientific and experiential knowledge in working judiciously and reflectively, and building systems and aligning resources that are centred on offenders’ health and social care needs.

**Conclusions:**

As part of a broader programme of work, a realist review can make an important contribution to the specification of theoretically informed interventions that have the potential to improve health outcomes. Our conceptual platform has potential application in related systems of health and social care where integrated, and person-centred care is a goal.

**Electronic supplementary material:**

The online version of this article (doi:10.1186/s13012-015-0321-2) contains supplementary material, which is available to authorized users.

## Background

This paper reports how we used a realist review, which aims to produce explanations of why mechanisms produce different patterns of outcomes in different contexts [1, 2], to develop a ‘conceptual platform’ specifying how an integrated, person-centred system to improve the mental health of offenders with common mental health problems is proposed to work. In realist terminology, a conceptual platform identifies the core set of processes within a class of interventions, how these processes operate, and the interactions between them [3]. In doing so, conceptual platforms have the potential to significantly increase the efficiency of intervention development and evaluation by providing a ‘recyclable’ core set of processes that can be judiciously applied in related areas [3]. In the example reported here, the conceptual platform is feeding into a wider project to develop, implement, and trial an intervention to improve collaborative mental health care for prisoners with common mental health problems. The review addresses the call for complex interventions with multiple, synergistic components, and which interact with context [4], to be properly theorised [5, 6].

Our use of a realist review is methodologically novel as it was conducted in parallel with the timelines and methods of a wider research project so as to inform intervention development. It therefore focused on extensive theory-building and refinement rather than the evaluation of these theories. In contrast to the use of social-psychological theory to inform the design of primary research that identifies and maps behavioural barriers to theoretical domains in clinical settings, for example, [7–10], this paper demonstrates how a realist review can be applied to inform complex intervention development that spans different locations, organisations, professions, and care sectors and incorporates organisational processes and practitioners’ interactions. To enable careful reflection and critique about how this may be achieved in practice, we provide considerable methodological detail.

International and national policies [11, 12] and guidelines [13] stress that a greater degree of integration between criminal justice, health, and social care systems is desirable to improve the mental health care for offenders, particularly in maintaining access to mental health care when prisoners are released into the community. However, the scientific literature does not provide coherent analyses of *how* this integration, in the form of collaborative care, can be achieved [14–16]. Prisoners with anxiety and depression (common mental health problems) may be impulsive and at risk of self-harm and returning to substance use after their release. Our prior hypothesis was that collaborative care for such prison leavers should include a number of components including the following: therapy; medication; ‘through the gate’ interaction with a link worker; resettlement such as housing, training, or employment support; and/or rehabilitation to change motivation or attitudes.

Our aim was to articulate, at both the organisational and individual levels, *how* an integrated, person-centred system that spans criminal justice and (mental) health and social inclusion service delivery is proposed to lead to improved outcomes. We applied a realist review in a novel way by focusing on the identification, articulation, and consolidation of explanatory accounts in order to inform intervention development. These explanatory accounts are analogous to ‘programme theory’ in that they express ideas about how a problem can best be addressed [17]. They can be seen as the ‘building blocks’ of broader, integrated theory about complex interventions. As such, they are large in number and cover a broad range of issues, including in this case the behaviour of different groups of individuals, how systems of care are organised between different sectors, and how organisational or community contexts enable or inhibit interactions or behaviour. We began by identifying over 300 such accounts, subsequently reducing them to 75 ‘consolidated’ explanatory accounts. Wherever possible, explanatory accounts made reference to the enabling and constraining factors (context) that impact on the operation of mechanisms and lead to outcomes (in realist terminology, context-mechanism-outcome configurations).

The explanatory accounts enabled us to produce a conceptual platform which will inform, together with analyses from focus groups and case studies, the development of an intervention that will be evaluated in a randomised-controlled trial (which is ongoing). We drew on sources beyond the fields of criminal justice and offender health (i.e. relating to other vulnerable populations) to develop explanatory accounts of context-mechanism-outcome configurations. As our goal in the review was to improve understanding of causal mechanisms across the system as a whole by specifying how multiple stakeholders understand and interact within it, our identification of explanatory accounts was therefore not limited to formal academic theory—indeed, the accounts of offenders, their families, and practitioners were crucial to understanding the operation of the system as a whole.

We conceptualised the proposed intervention as involving two steps, engaging first with practitioners (and the environment in which they work). Practitioners would then, through their changed behaviour and actions, engage with and support offenders in new and more effective ways. This conceptualisation was based on our previous experience and knowledge of working and researching with offenders. The intervention would bring about its effects by first influencing the organisational opportunities and constraints. This would enable practitioners to change their behaviour, which in turn could then influence offenders’ behaviour (whilst taking account of their motivations and capabilities) [18]. This approach combines realist and behaviour change approaches and is also consonant with the World Health Organization’s ‘roadmap’ for people-centred health systems [19]. We proposed that intervention practitioners would work with individual offenders for a time-limited ‘pathway’ based on identification prior to release, support ‘through the prison gate’, and support in the community. We envisaged that the intervention would be judiciously modifiable to local contexts; link to, and support, co-ordination of existing resources (rather than being a stand-alone, all-encompassing service); and provide some form of ongoing care and support (rather than primarily having a triage and referral function as is currently advocated [20]). Whilst we did not envisage the intervention involving structural change within prisons [21, 22], we did envisage that it would require some changes to the operating environment to support specific practitioner behaviours. It was also based on the need to overcome the fundamental deficit in prisons whereby mental health issues remain unrecognised and unaddressed [15].

In summary, our aim was to articulate, at both organisational and individual levels, *how* an integrated, person-centred system that spans criminal justice and (mental) health and social inclusion service delivery is proposed to lead to improved outcomes. In the following pages, we document how we applied the realist review, including the identification and consolidation of explanatory accounts. The findings are presented as a narrative description of the conceptual platform and its relation to the structure of the envisaged intervention.

## Methods

A realist review [1] is grounded in a realist philosophy of science which holds that it is possible to discern generative mechanisms within the social systems in which they operate [23, 24]. It is characterised by taking an iterative approach to the identification, appraisal, and synthesis of diverse forms of evidence in the form of programme theories. The aim is to explain how mechanisms (the way in which a programme’s resources or opportunities interact with the reasoning of individuals and lead to changes in behaviour [25]) produce different patterns of outcomes in different contexts (the wider configuration of factors that enable or constrain the operation of mechanisms) [1, 2]. The full protocol for the review, which includes the initial ‘two-step’ conceptualisation of the intervention and programme theory, is registered on the PROSPERO database (CRD42012002640). The review is reported in accordance with the RAMESES publication standards for realist reviews [26].

### Search strategy

The search strategy was designed to locate and retrieve relevant information from a range of published and unpublished sources. This strategy, informed by the formative models presented in the protocol, started with hand-searching a core set of 16 journals to identify sources that could inform citation chasing, website and database searches, and elicitation from experts (including academics, practitioners, and men with lived experience of prison and release (referred to as peer researchers)). Our searches were developed iteratively and explicitly included sources from a range of fields so that learning from criminal justice, mental health care, and health and social inclusion service delivery to vulnerable groups could be incorporated. We conducted the search strategy in stages, progressing from searches focusing on offenders, to vulnerable groups, and then to mental health. Full details of the search strategy are reported in Additional file 1.

### Screening

We first included all sources that provided rich descriptions (see criteria in Additional file 2) of the content and delivery of interventions that proposed a form of collaborative care for offenders with common mental health problems in any Organisation for Economic Co-operation and Development (OECD) country *or* that proposed ways in which intervention components or service configurations achieve, or could achieve, positive outcomes. Sources eligible for inclusion included (but were not limited to) editorials, opinion pieces, commentaries, comparative effectiveness studies, process evaluations, qualitative research, and systematic reviews. In screening, we erred on the side of inclusivity so that components of less holistic forms of collaborative care could still contribute explanatory accounts.

Where insufficient sources relating to offenders were located to enable identification of explanatory accounts, we translated these inclusion criteria to the screening of sources about vulnerable groups, and then mental health. In applying the inclusion criteria, we were aware of the risk of excluding relevant sources if applied mechanistically—we therefore operationalised our inclusion criteria in the form of whether or not sources contained or tested theories that addressed any of the components in the initial model proposed in our protocol. This also facilitated the screening of sources not directly related to the care of offenders.

To inform decision-making in the review, we produced a ‘map’ of all sources identified for potential inclusion through classifying the sources by topic, type of publication, and whether professional or lived experience of people were the source of the explanatory accounts. We also classified all sources by relevance (potential contribution to building explanatory accounts) and ‘rigour’ (Additional file 2).

**Fig. 1:**
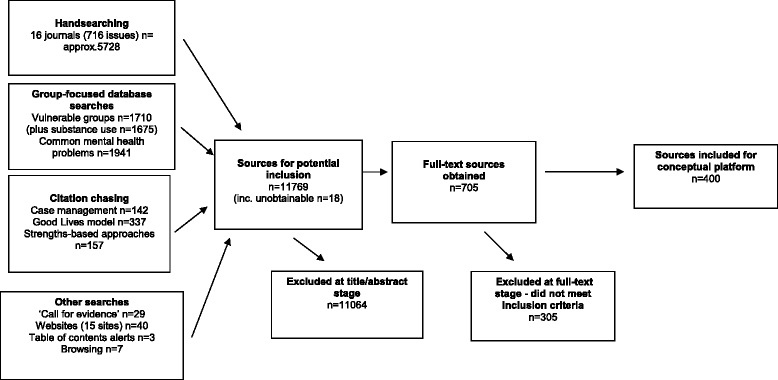
Flowchart of sources through the review

We acknowledge that limits to the extent and depth of information contained in abstracts placed constraints on the accuracy to which we could classify sources. We therefore erred on the side of caution and included sources where it was not possible to establish clearly whether or not they were includable or the precise way in which they should be classified. The flow of sources through the review is shown in Fig. 1.

### Identifying explanatory accounts

We located explanatory accounts in a range of sources (primary studies, opinion pieces, grey literature) and in a range of locations within sources, such as study findings and researchers’ reflections. Explanatory accounts were also identified at a meeting with a peer researcher group of men with lived experience of prison and release (as part of a series of 18 group meetings) and a study group of practitioners and academics (which met once during the review and subsequently corresponded by email). We used Jackson et al.’s [27] strategy for identifying explanatory accounts by ‘working backwards’ from outcomes, both intermediate and final and personal or organisational. Frequent interaction between the two reviewers (MP and SB) took place to enable consistency of application and to address ongoing issues with the application of the strategy.

Identified explanatory accounts were recorded in a table together with a record of the source (academic, practitioner, peer researcher, offender, significant other of offender, or policy maker—see Additional file 3). Wherever possible, we expressed explanatory accounts in the form of ‘If… then’ statements which specified context and mechanism. For example: *If* young homeless people have a deep mistrust of services from their experiences in childhood, *then* their trust in, and engagement with, services in adult life is severely limited (#52). However, we did not limit accounts to only those that could be expressed in this way as we recognised that partial accounts about context could still be informative. For example: Many prisoners were less willing to remain in services on release—in prison, the service had filled their day, but in the community, there were so many other factors affecting their lives (#103). The table enabled both reviewers to comment on and identify inter-relationships and overlaps between explanatory accounts before further development.

To maintain a balance between comprehensiveness and achievable review outputs, we did not record explanatory accounts that substantively repeated or overlapped with earlier accounts but did retain these sources for potential later use. Additional file 3 lists the 347 explanatory accounts located.

### Consolidating and expressing explanatory accounts

We aimed to integrate explanatory accounts in order to produce the most economical expression of context-mechanism-outcome configurations. We initially tried to synthesise using all of the processes of reasoning (juxtaposition, reconciliation, adjudication, consolidation, and situating) suggested by Pawson [1], but given the breadth and complexity of the explanatory accounts, we found this process unwieldy and problematic to document. Instead, we structured our synthesis using a set of questions (below) that focused on the mechanism or outcome within explanatory accounts.

We (MP and SB) agreed on an initial classification of the explanatory accounts according to the components of our initial model [28]. We jointly read the explanatory accounts, for each one asking the following:

- Is this account novel? (and therefore reasonable to ‘import’ directly into the consolidated explanatory accounts table)

If the account was not entirely novel, then our discussion was prompted by asking the following questions:

- Does this account challenge the explanations made in related accounts?
- Does this account add important refinements to the understanding of contexts, mechanisms, or outcomes made in related accounts?

To e*xpress* the consolidated accounts, we asked the following questions:

- Is an aspect of an account novel and therefore possible to transfer verbatim to a consolidated account?
- Is a context, mechanism, or outcome sufficiently similar to that in a consolidated account to warrant integration into that account under a different term? Or should the consolidated account be changed to reflect the new term and the increased explanatory power it offers?
- Does the revised consolidated explanatory account sufficiently reflect the context-mechanism-outcome configurations proposed in the underlying explanatory accounts?

This process of synthesis hinged upon the openness and commitment of the reviewers (MP and SB) to purposefully challenge the developing consolidated explanatory accounts. Central to this was a working relationship that valued constructive debate and which made time for the investigation and resolution of disagreements. Two worked examples of consolidating by mechanism and outcome are shown in the animation and narration in Additional file 4.

During the synthesis process, we shared a selection of the consolidated explanatory accounts with our expert group of practitioners and academics so that feedback about their expression and scope could be incorporated. Additional file 5 lists the 75 consolidated explanatory accounts, which were classified by the type of context or interaction so as to facilitate the use of the accounts in intervention development. The sources from which the consolidated explanatory accounts were developed are shown in Additional file 6.

We developed the conceptual platform, which is described narratively in the ‘Findings’ section below and summarised in bullet points at the end, with a view to the eventual intervention being a two-stage process. This involves engaging first with practitioners (and the environment in which they work) so that they can then, through their changed behaviour and actions, engage with offenders. We envisaged this two-stage process involving feedback loops, emergence, and non-linearity.

## Findings

**Fig. 2:**
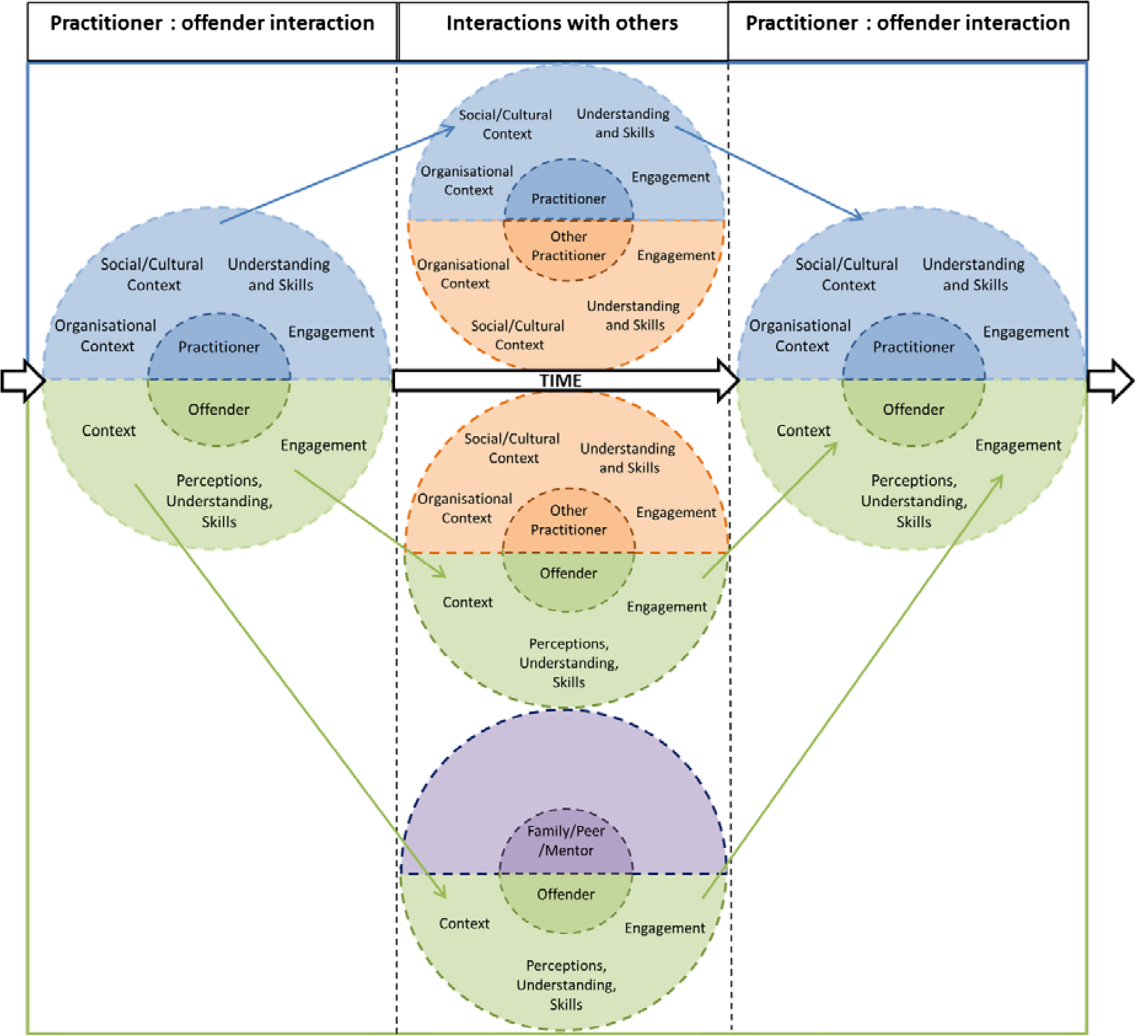
Overview of an integrated, person-centred system to improve collaborative mental health care for offenders

An overview of how the conceptual platform is integrated with the structure of the envisaged intervention is shown in Fig. 2. The three columns delineate different key interactions over time, before and after release, whilst participating in a mental health improvement intervention. Dashed lines bound the interactions during which mechanisms activate. These contextualised interactions can be between practitioners and offenders, between the practitioner/offender and other practitioners, or between the offender and family members, peers, or mentors. At the focal point of the intervention are the core interactions between intervention practitioners and offenders. It is within these interactions that the effect of the intervention on *practitioners*’ behaviour, thinking, and emotion has the potential to affect *offenders*’ behaviour, thinking, and emotion. In between these core interactions, the interactions of both the practitioner and offender with other people (the central three circles in the graphic) affect change in their behaviour, thinking, and emotions and how these interact with their contexts. These changes impact on the subsequent interaction between the practitioner and offender and generate other potentially beneficial effects.

Categories within the semi-circles show the headings under which the 75 consolidated explanatory accounts (referred to in parentheses; listed in Additional file 4) are presented below. The narrative below presents the conceptual platform for how an intervention to promote mental health across institutional and community environments theoretically operates.

### Practitioners—organisational context

Practitioners work within organisations, and the day-to-day operation of organisations impacts on the extent to which practitioners can deliver services that are person-centred. If there is congruence between the goals and values of practitioners and the organisation in which they work, then the resources provided by the intervention are more likely to be used in the way intended (consolidated explanatory accounts 43, 61). However, as delivery of a person-centred intervention is dependent upon different organisations working together collaboratively, the infrastructure to support practitioners’ work is key. At a *strategic* level, attaining agreement between organisations about common purpose is a necessary first step (1, 61) but is insufficient without the practical elements that enable these to be achieved day to day. Such elements can include pooled budgets (56) and collaboratively developed formal agreements about information sharing, assessment tools, roles, and responsibilities (1, 52) and the authority to hold practitioners in other organisations to account (52, 58). At an *operational* level, practitioners need to be given the opportunity to develop their skills (for example, in relation to trauma and self-harm (39, 50)) and have their practice supported by organisational systems that both enable monitoring of individuals in need of care and provide feedback and support that facilitates practitioners’ skill development (39).

Maintaining congruence at both strategic and practitioner levels is vital. An agreement developed between organisations at a strategic level risks piecemeal implementation if it is not congruent with the goals and values of practitioners (61). Organisational agreements do not function solely through their formal status but because they are seen as relevant and workable by practitioners across different organisations. The development of working relationships that can support this inter-organisational congruence may be facilitated by basing practitioners in the same location (53).

### Practitioners—social/cultural context

Practitioners rarely consciously decide to work in a non-collaborative way, but non-collaborative practice can arise from the decisions that practitioners make within the organisational and incentive structures, and cultural contexts, in which they work. Collaborative practice can therefore be supported through a range of facilitative organisational measures. If practitioners understand their role, responsibilities, and the contribution that they make within a system of care and around a particular offender, they are more likely to be able and willing to work collaboratively (25). Clear agreements between organisations about these factors, together with the information and communication systems that enable them to be put into practice, are also necessary but not sufficient on their own (2, 25).

### Interactions between practitioners and practitioners

Information-sharing and care planning (for the delivery of collaborative care) is not a passive process of diffusion between practitioners working in different locations or care sectors. Even though communication systems and inter-organisational agreements may facilitate information-sharing and care planning (52), they do not eclipse the importance of practitioners’ working relationships both within and beyond their immediate working environment. Collaborative working between practitioners therefore has a *relational* aspect but also a *knowing* aspect about the operation of the care system as a whole. These aspects are mutually reinforcing—for example, knowing *who* to contact and *how* is insufficient without a practitioner believing that his/her referral will be welcomed (2). A referral that is welcomed provides an opportunity for relationship building (17, 22). Referrals, training, and supervision can support development of a shared language (24) and greater understanding of the care system as a whole and practitioners’ roles within it (22, 32, 37). In summary, an important part of initiating and maintaining collaborative working is fostering both the *knowing* and *relational* aspects of collaborative working.

### Practitioners—engagement

Interventions and the associated changes in practice do not follow in a straightforward sense from a decision at an organisational level to introduce them. The actions of practitioners are pivotal, as it is through such actions that interventions are made on a day-to-day basis. As practitioners are not passive, their engagement in proposed changes in practice is crucial. This engagement can take place on a number of different levels, ranging from the individual (e.g. facilitating practitioners to feel proud of their work) to the team level (e.g. feeling supported and trusted by colleagues) and through to the organisational level (enabling practitioners to pursue personal and professional goals) (5). The relative importance of addressing each of these levels is unclear, but it may be that it is simply necessary to ensure that all of these levels of engagement are recognised and addressed as judged appropriate in the local context. It is worth bearing in mind that the rationales that practitioners employ in their decision-making are likely to be constrained or enabled by these local contexts, in particular whether or not the work environment is experienced as supportive and colleagues and supervisors are trusted (5).

The extent of concordance between practitioners’ and perceptions at an organisational level of the need (or not) for changes in practice is a key explanatory element of how engagement can take place (6). The extent of this concordance can be first flushed out by acknowledging the potential contribution of practitioners’ *experiential knowledge* to the development of proposed service changes and incorporating this knowledge as appropriate (8). This enables practitioners to feel that they have contributed substantively to the development of, and have an ongoing part to play in the implementation of, the proposed service changes. Second, as practitioners’ *motivations* are both intrinsic (such as practising in a way consistent with their personal values and which gives them pride in a ‘job well done’) and extrinsic (such as the approval of colleagues or the financial rewards associated with practising at a higher level of expertise), then practitioners need to believe that there is concordance between achieving these goals and their participation in proposed service changes. Third, as day-to-day work is usually structured in a way that reflects different practitioners’ current roles, status, and degree of autonomy, proposed service changes that challenge these traditional ways of working can demonstrate a significant lack of concordance between practitioners’ and an organisation’s perceptions about roles and responsibilities (7). The extent to which it is perceived that an intervention *challenges conventional practice* can therefore impact negatively on practitioner engagement.

### Practitioners—understanding and skills

Two aspects of practitioners’ understanding and skills were identified as impacting upon their willingness and ability to develop positive relationships with service users. The first relates to *knowing about* mental health and how mental health problems manifest in people’s behaviour (50). For example, the tension between custody and treatment models can be brought into sharp relief by differences in opinion about how to practice held by health care and criminal justice practitioners (19). The second aspect relates to *knowing how* to develop supportive relationships with people with mental health problems. This is grounded in knowing about mental health but also requires support to develop practitioners’ ability to practise empathically day to day (36) and to continue to do so through supervision that supports practitioners to learn from reflecting on their own practice (28). The examination of assumptions that underpin practise can inform practitioners’ relationship-building in a way that supports offenders’ transitions into the community. For example, if practitioners *assume* that offenders’ families can offer the same social and emotional support that their own family could provide, then their potential for supporting offenders to mobilise their own social capital is reduced (41).

### Interactions between practitioners and offenders

Engagement is defined by its flexible nature. This can manifest in a number of ways, all of which demonstrate to the individual that their needs and views are taken seriously. For example, recognition of an individual’s unique history and its relevance to their current situation can be demonstrated by accurately reflecting back what has been said (67). Initial engagement may need to strike a balance between recognising past experience (which may be negative) and a potentially positive experience of services in the future (34). Keeping individuals engaged will require ongoing, demonstrably credible actions that achieve access to the range of services that an individual requires to support their mental health both in prison and on release (15, 35).

A genuine recognition of offenders’ individuality is the lodestar that can guide practitioners’ interactions with offenders in a way that promotes engagement and a network of actions and relationships that promote mental health. The core mechanism at play is the motivation that individuals gain from being involved in a supportive working relationship that recognises the humanity, strengths, and particular challenges they face. This mechanism is particularly powerful where offenders experience prison as disempowering and lacking in people that care, as the power difference between offenders and practitioners is reduced (75). The relationship begins at the outset of collaborative care formulation by focusing on how to balance working towards an individual’s goals with evidence-informed treatments (10, 12, 62) and the negotiation of access to services to provide that care (13, 48). Such an approach works towards building on the individual’s strengths, although consideration also needs to be given to the way that an offender’s gender, ethnicity, or religion/spirituality is part of their identity. It is vital to understand how these contextual aspects of identity impacts on an individual’s journey towards improved mental health (64, 65). Offering a choice to the individual as to when meetings take place can facilitate initial engagement, and accurately reflecting back what an individual has discussed can demonstrate understanding and empathy (67). The initial recognition and care formulation is just the first step of the journey towards resettlement, rehabilitation, and mental health on which practitioners can accompany offenders.

The metaphor of accompanying the individual on a journey is useful. The path may be long but has a reachable destination, and travelling along it with others will help to get over the lows and reach the highs. Accompanying a person on this journey requires practitioners to not confine therapeutic interactions to formal therapy sessions (33) and to work flexibly by increasing or decreasing their level of support as appropriate for the individual at different stages (64). The support a practitioner should provide is distinguished by not being judgmental or stigmatising (27, 42) and functions by providing a coherent ‘bridge’ between an individual’s current identity and the future identity they want (27).

The principles of mentalisation-based therapy (MBT) [29] can help structure interactions between practitioners and offenders even when not using MBT as a formal therapeutic approach. An offender’s ability to understand the relationship between their thoughts, emotions, and actions can be enabled by a practitioner’s ability to understand, recognise, and manage the impact of an offender’s mental state (in particular their level of arousal) on their ability to interact. Attaining a non-judgemental understanding of the links between one’s own (and other’s) thoughts, emotions, and actions (‘mentalisation’) involves the practitioner enabling the offender to make use of their own capacities for reflection and future planning (72, 74). Interactions characterised by a willingness to explore issues (rather than simply transfer expert knowledge) and support individuals to attend to their own feelings (rather than identifying and naming these feelings) should support the process of mentalisation (74). Such interactions require a delicate balance to be struck between intellectual analysis and emotional involvement, as both of these capabilities are needed to reflect on the links between thoughts, emotions, and actions and how a person may wish to act differently in the future (72). The creation of a safe and sensitive interpersonal environment is necessary for a person to have the confidence to reflect and ‘mentalise’ whilst regulating his/her emotional state (73).

Remaining responsive to an individual’s circumstances is a key aspect that permeates the supportive relationship. This can manifest in a variety of ways, including maintaining sensitivity to the appropriateness of individual or group work (47); providing care that is sensitive to the unique needs of individuals who have experienced trauma (39); facilitating self-expression across a range of psychological needs through, for example, art therapy (45); and recognising offenders’ efforts to progress (63). When, for whatever reason, the supportive relationship falters, if the practitioner takes the time to address the reasons for this happening, then the risk of discontinuity is reduced (29).

However, the practitioner is not the sole actor in providing the breadth and depth of the supportive relationship described above. Practitioners’ support can provide the foundation and stimulus for the individual to repair or create their own supportive relationships with significant others or peers (30), and/or practitioners can encourage and enable significant others and peers to provide support for the individual that can endure long beyond the end of the practitioner’s supportive relationship (30, 42, 64). In short, the practitioner has a key role to play in cultivating facilitative contexts that allow the supportive community mechanisms of relationships with family and friends to operate.

### Offenders—organisational context

For offenders, the prison environment can set the tone for all of the interactions that take place within it and therefore the extent to which offenders are motivated to engage with services. A facilitative environment is characterised by an organisational environment that offers choice in, and access to, services (16). It is also evident in the behaviour of prison staff and the interactions they have with offenders—this can take the form of explaining and consistently applying rules and demonstrating tolerance in interactions with offenders (16). The development of supportive relationships can be assisted when teams of professionals are themselves diverse (for example, in gender and culture), as this increases opportunities for the development of client-practitioner relationships where the client has a particular connection with, or identifies with, a practitioner’s life experiences (57). Although there are clearly restrictions within the prison environment in terms of depriving individuals of their liberty, it can still set a supportive tone for engagement by supporting the common human drive to find meaning in daily activities such as work and exercise (49).

### Offenders—perceptions, understanding, and skills

Two key sequential steps in an offender’s progress towards improved mental health are their constructive engagement with services and the cultivation of skills that enable self-care. Engagement requires *trust* in both individual practitioners and the system in which these practitioners work. Offenders need to have reason to believe that, if they approach and engage with practitioners, they will be treated empathically and fairly (4) and that by discussing mental health issues that may lead to treatment they are not risking a negative impact on the length of their sentence (3). Similarly, offenders need to have reason to believe that communication between agencies is timely and accurate so that care is provided in a co-ordinated manner (4, 32). It is suggested that differences in the perception of organisational boundaries between offenders and practitioners, with offenders seeing ‘one service provided by different people’ and practitioners seeing ‘many separate services with separate provision’, are one reason why offenders’ trust in practitioners and the system can falter (32). If practitioners explore offenders’ concerns (based on their prior experiences), then there is an opportunity to begin engagement even though imperfect service provision is the reality (31).

Engagement provides the foundation for cultivating the skills that will enable self-care. It is suggested that developing ‘mindfulness’ skills, the ability to be aware of one’s own mood state and its relationship with what is currently happening, can underpin the development of other mental health self-care skills (46). In this way, and in conjunction with the development of communication and social skills, an upward self-supporting spiral is initiated—self-awareness can increase receptivity to learning new mental health skills, which increase empathic skills and an offender’s ability to form or re-connect with a supportive social environment, which promotes efforts towards resettlement and rehabilitation (26) and so on. *Inspiration by* and *emulation of* others who have had similar experiences can increase ability to self-manage and take personal responsibility (71).

### Summary of the conceptual platform

Our findings can be summarised in the form of a conceptual platform, specifying the *core set of processes* of how an integrated, person-centred system to improve the mental health of offenders with common mental health problems is proposed to work. Such a system works through the following:

- Different systems, in particular health and criminal justice, having goals that are consistent with one another
- Attaining consistency between strategic goals and the goals of practitioners
- Making referral pathways and links between organisations comprehensible to practitioners and providing opportunity for the development of constructive working relationships
- Practitioners being facilitated and enabled to balance factors that can be in tension—for example, ‘knowing how’ as well as ‘knowing that’, analysing one’s own behaviour whilst remaining attentive to emotions, and working towards an individual’s goals
- Practitioners being facilitated and enabled to apply scientific and experiential knowledge judiciously in working with individual offenders, colleagues, and the systems in which services are delivered
- Practitioners having sufficient knowledge about mental health and how to develop supportive relationships with people with mental health issues
- Recognising the individuality of offenders throughout all interactions in the criminal justice, health, and social care systems
- Aligning resources so as to facilitate offenders to achieve their collaboratively agreed goals
- Practitioners supporting reconnection with, and/or development of, networks of support outside of prison
- Offenders having reasons to trust practitioners, services, and systems

## Discussion

Drawing on sources both within and beyond the fields of criminal justice and offender health, we have developed explanatory accounts of context-mechanism-outcome configurations relating to offenders’ mental health (reported in the ‘Findings’ section). Our findings show how the ideal of delivering an integrated, person-centred system to improve collaborative mental health care for offenders inherently operates through the constrained or enabled decision-making of both practitioners and offenders and how this takes place at the interstices of multiple cross-cutting relationships and systems.

This paper’s distinctive contribution is to bring together these explanatory accounts in concert so as to further understand the ways in which sustainable change can be attained within a complex system. Mapping the theories that can underpin change within a complex system makes the route towards achieving sustainable change clearer, albeit not that much less daunting.

We are using these accounts to inform the development of an intervention to improve collaborative mental health care for offenders with common mental health problems. In addition to the *instrumental* use of the knowledge constructed in this review to contribute to intervention development, there is an important *conceptual* use of this knowledge [30] to prompt critical re-thinking about the way that collaborative care is organised to improve collaborative mental health care for offenders with common mental health problems. The conceptual platform presented in this paper can also be used to stimulate a wide range of stakeholders (service users, practitioners, commissioners, and policy makers) to think differently about how mental health care for offenders is designed and delivered. It also has potential application outside of offender health care in fields of practice where collaborative and integrated working is required, particularly where services are delivered across sectors and professional groups, and where interventions need to complement and not replace existing service and voluntary resources. The integrated care of people with long-term conditions is one area where the conceptual platform may have particular resonance, although it is unlikely to be ‘transferable’ in its entirety.

Our use of a realist review to identify and articulate in detail the accounts that explain how a complex system to improve mental health care of offenders could operate is a novel application of the approach. We found that a realist approach provided a common language and logic of inquiry that enabled us to confidently go beyond a single field (offender health care) in our search for, and articulation of, these accounts. Whilst precise identification of contexts, mechanisms, and outcomes eluded us at times, we found using the working definitions of these concepts highly important for structuring our inquiry and enabling us to discuss, debate, and develop the accounts. The language of ‘contexts, mechanisms, and outcomes’, which we primarily expressed in the form of ‘If…then’ statements, was also straightforward enough to enable us to access both the lived experience of the peer researcher group and the academic and practitioner knowledge of the advisory group in a form that was consistent with the other explanatory accounts we were identifying and articulating in the review.

### Strengths and limitations

Whilst we made significant efforts to ensure transparency in this study, we acknowledge that this is a difficult task in a realist review and we do not claim to have been able to document all of the many decisions that were made in the course of conducting this review. The high number of sources identified and obtained in this review, the variety of presentation and reporting, and the often ‘hidden’ nature of accounts within these sources mean that there is a risk that we missed relevant theories, even though we shared emerging findings with our advisory group throughout the review. The selection of mentalisation-based therapy as a key component was made prior to the review, based on an appraisal of potential therapeutic approaches for individuals with mixed mental health problems; the magnitude of psychological literature precluded a full review of all potential approaches. Given that we did not identify any significant ‘competing’ accounts, there is also a risk that we were not fully aware of how our own conceptualisation of issues impacted on our reading of sources, although we endeavoured to examine and make plain our initial conceptualisations at the outset. Finally, our conceptual platform does not include any accounts relating to the resource implications of changes in service delivery.

## Conclusion

We have demonstrated how a realist review can be applied and adapted for the purposes of specifying the essential components of how an integrated, person-centred system to improve offenders’ mental health is proposed to work. In providing this worked example, we have contributed substantively both to understanding of how integrated, person-centred systems are proposed to work and the development of methods that can underpin theory-driven intervention development [4–6]. Our approach to synthesis, which built on the understandings and insights of both academic theory and the lived experience of both practitioners and offenders, complements approaches to theory synthesis that focus solely on academic theory [31].

### Endnote

See additional file 4 for references from which consolidated explanatory accounts were identified (referred to in parentheses in manuscript).

### Availability of supporting data

The data sets supporting the results of this article are included within the article and its additional files.

## Additional files

Additional files 1:
**Search strategy.**
Additional file 2:
**Screening tools.**
Additional file 3:
**Explanatory accounts.**
Additional file 4:
**Consolidating accounts—narration.**
Additional file 5:
**Consolidated explanatory accounts.**
Additional file 6:
**Sources of explanatory accounts.**
