# Supplementary material for: Using realist review to inform intervention development: methodological illustration and conceptual platform for collaborative care in offender mental health
Source: Implement Sci. 2015 Sep 28;10:134. doi: 10.1186/s13012-015-0321-2 (PMC4584430; doi:10.1186/s13012-015-0321-2)
Supplement: Additional files 1: — Search strategy. [file 13012_2015_321_MOESM1_ESM.docx]

**Supplementary File 1: Search Strategies**

1. COMMON MENTAL HEALTH PROBLEMS

Database: MEDLINE

Host: Ovid

Data Parameters: 1946 to October Week 5 2013

Date Searched: 13/11/2013

Searcher: SB

Hits: 1360

Strategy:

1. ("common mental health" adj2 (problem* or disorder*)).tw.
2. (CMHP or CMHD).tw.
3. (depression or depressed or depressive or melanchol*).tw.
4. Depression/
5. Depressive disorder/
6. (anxiety or phobi* or "panic disorder*").tw.
7. Anxiety/
8. Phobic Disorders/
9. Panic Disorder/
10. ("post-traumatic stress disorder*" or "posttraumatic stress disorder*" or PTSD).tw.
11. Stress Disorders, Post-Traumatic/
12. ("obsessive compulsive disorder*" or OCD).tw.
13. Obsessive-Compulsive Disorder/
14. or/1-13
15. ((share? or sharing or integrat* or collabora* or continuity) adj3 (care or healthcare or service? or provision? or practice?)).tw.
16. 14 and 15
17. limit 16 to english language

Database: PsycINFO

Host: Ovid

Data Parameters: 1806 to November Week 1 2013

Date Searched: 13/11/2013

Searcher: SB

Hits: 1141

Strategy:

1. ("common mental health" adj2 (problem* or disorder*)).tw.
2. (CMHP or CMHD).tw.
3. (depression or depressed or depressive or melanchol*).tw.
4. major depression/
5. (anxiety or phobi* or "panic disorder*").tw.
6. Anxiety/
7. exp phobias/
8. Panic Disorder/
9. ("post-traumatic stress disorder*" or "posttraumatic stress disorder*" or PTSD).tw.
10. posttraumatic stress disorder/
11. ("obsessive compulsive disorder*" or OCD).tw.
12. Obsessive-Compulsive Disorder/
13. or/1-12
14. ((share? or sharing or integrat* or collabora* or continuity) adj3 (care or healthcare or service? or provision? or practice?)).tw.
15. 13 and 14
16. limit 15 to english language

Database: CINAHL

Host: EBSCO

Data Parameters: n/a

Date Searched: 13/11/2013

Searcher: SB

Hits: 819

Strategy:

1. TI ("common mental health" N1 (problem* or disorder*)) OR AB ("common mental health" N1 (problem* or disorder*))
2. TI ( CMHP or CMHD ) OR AB ( CMHP or CMHD )
3. TI(depression or depressed or depressive or melanchol*) OR AB(depression or depressed or depressive or melanchol*)
4. (MH "Depression")
5. TI (anxiety or phobi* or "panic disorder*") OR AB (anxiety or phobi* or "panic disorder*")
6. (MH "Anxiety")
7. (MH "Phobic Disorders")
8. (MH "Panic Disorder")
9. TI ( "post-traumatic stress disorder*" or "posttraumatic stress disorder*" or PTSD ) OR AB ( "post-traumatic stress disorder*" or "posttraumatic stress disorder*" or PTSD )
10. (MH "Stress Disorders, Post-Traumatic")
11. TI ( "obsessive compulsive disorder*" or OCD ) OR AB ( "obsessive compulsive disorder*" or OCD )
12. (MH "Obsessive-Compulsive Disorder")
13. S1 OR S2 OR S3 OR S4 OR S5 OR S6 OR S7 OR S8 OR S9 OR S10 OR S11 OR S12
14. TI(share* or sharing or integrat* or collabora* or continuity) N2 (care or healthcare or service* or provision* or practice*) OR AB(share* or sharing or integrat* or collabora* or continuity) N2 (care or healthcare or service* or provision* or practice*)
15. S13 AND S14

Notes: Limited to English language

Database: Cochrane library

Host: Cochrane Collaboration

Data Parameters: CDSR and CENTRAL: Issue 10 of 12; DARE and HTA: Issue 4 of 4

Date Searched: 14/11/2013

Searcher: SB

Hits: CDSR=6; CENTRAL=244; DARE=7

Strategy:

1. ("common mental health" near/1 (problem* or disorder*)):ti or ("common mental health" near/1 (problem* or disorder*)):ab
2. (CMHP or CMHD):ti or (CMHP or CMHD):ab
3. (depression or depressed or depressive or melanchol*):ti or (depression or depressed or depressive or melanchol*):ab
4. MeSH descriptor: [Depression] this term only
5. MeSH descriptor: [Depressive Disorder] this term only
6. anxiety or phobi* or "panic disorder*":ti or anxiety or phobi* or "panic disorder*":ab
7. MeSH descriptor: [Anxiety] this term only
8. MeSH descriptor: [Phobic Disorders] this term only
9. MeSH descriptor: [Panic Disorder] this term onl
10. "post-traumatic stress disorder*" or "posttraumatic stress disorder*" or PTSD:ti or "post-traumatic stress disorder*" or "posttraumatic stress disorder*" or PTSD:ab
11. MeSH descriptor: [Stress Disorders, Post-Traumatic] this term only
12. "obsessive compulsive disorder*" or OCD:ti or "obsessive compulsive disorder*" or OCD:ab
13. MeSH descriptor: [Obsessive-Compulsive Disorder] this term only
14. #1 or #2 or #3 or #4 or #5 or #6 or #7 or #8 or #9 or #10 or #11 or #12 or #13
15. ((share* or sharing or integrat* or collabora* or continuity) near/2 (care or healthcare or service* or provision* or practice*)):ti or ((share* or sharing or integrat* or collabora* or continuity) near/2 (care or healthcare or service* or provision* or practice*)):ab in Cochrane Reviews (Reviews and Protocols), Other Reviews, Trials and Technology Assessments
16. #14 and #15

**Number of references retrieved and duplicates**

| **Database** | **Hits** |
| --- | --- |
| MEDLINE | 1360 |
| PsycINFO | 1141 |
| CINAHL | 819 |
| CDSR | 6 |
| CENTRAL | 244 |
| DARE | 7 |
| HTA | 0 |
| **Total** | **3577** |
| **Duplicates** | **1636** |
| **Total to screen** | **1941** |

1. SUBSTANCE ABUSE

Database: MEDLINE

Host: Ovid

Data Parameters: 1946 to October Week 5 2013

Date Searched: 13/11/2013

Searcher: SB

Hits: 1092

Strategy:

1. ((alcohol* and (misus* or abuse* or "problem use*" or "problem drink*" or "illicit use*" or addict* or dependen* or dependant or delinquency)) or (alcoholic$1 or alcoholism)).tw.
2. exp Alcohol-Related Disorders/
3. Alcoholics/
4. ((drug* or substance) and (illegal or misus* or abuse* or "problem use*" or "illicit use*" or addict* or dependen* or dependant or delinquency)).tw.
5. exp Substance-Related Disorders/
6. Drug users/
7. or/1-6
8. ((share? or sharing or integrat* or collabora* or continuity) adj3 (care or healthcare or service? or provision? or practice?)).tw.
9. 7 and 8
10. limit 9 to english language

Database: PsycINFO

Host: Ovid

Data Parameters: 1806 to November Week 1 2013

Date Searched: 13/11/2013

Searcher: SB

Hits: 971

Strategy:

1. ((alcohol* and (misus* or abuse* or "problem use*" or "problem drink*" or "illicit use*" or addict* or dependen* or dependant or delinquency)) or (alcoholic* or alcoholism)).tw.
2. alcoholism/
3. ((drug* or substance) and (illegal or misus* or abuse* or "problem use*" or "illicit use*" or addict* or dependen* or dependant or delinquency)).tw.
4. exp drug abuse/
5. or/1-4
6. ((share? or sharing or integrat* or collabora* or continuity) adj3 (care or healthcare or service? or provision? or practice?)).tw.
7. 5 and 6
8. limit 7 to english language

Database: CINAHL

Host: EBSCO

Data Parameters: n/a

Date Searched: 14/11/2013

Searcher: SB

Hits: 444

Strategy:

1. TI ((alcohol* and (misus* or abuse* or "problem use*" or "problem drink*" or "illicit use*" or addict* or dependen* or dependant or delinquency)) or (alcoholic* or alcoholism)) OR AB ((alcohol* and (misus* or abuse* or "problem use*" or "problem drink*" or "illicit use*" or addict* or dependen* or dependant or delinquency)) or (alcoholic* or alcoholism))
2. (MH "Alcohol-Related Disorders+")
3. (MH "Alcoholics")
4. TI (drug* or substance) and (illegal or misus* or abuse* or "problem use*" or "illicit use*" or addict* or dependen* or dependant or delinquency) OR AB (drug* or substance) and (illegal or misus* or abuse* or "problem use*" or "illicit use*" or addict* or dependen* or dependant or delinquency)
5. (MH "Substance Abuse+")
6. S1 OR S2 OR S3 OR S4 OR S5
7. TI (share* or sharing or integrat* or collabora* or continuity) N2 (care or healthcare or service* or provision* or practice*) OR AB (share* or sharing or integrat* or collabora* or continuity) N2 (care or healthcare or service* or provision* or practice*)
8. S6 AND S7

Notes: Limited to English language

Database: Cochrane library

Host: Cochrane Collaboration

Data Parameters: CDSR and CENTRAL: Issue 10 of 12; DARE and HTA: Issue 4 of 4

Date Searched: 14/11/2013

Searcher: SB

Hits: CDSR=1; CENTRAL=58

Strategy:

1. (alcohol* and (misus* or abuse* or "problem use*" or "problem drink*" or "illicit use*" or addict* or dependen* or dependant or delinquency)) or (alcoholic* or alcoholism):ti or (alcohol* and (misus* or abuse* or "problem use*" or "problem drink*" or "illicit use*" or addict* or dependen* or dependant or delinquency)) or (alcoholic* or alcoholism):ab
2. MeSH descriptor: [Alcohol-Related Disorders] explode all trees
3. MeSH descriptor: [Alcoholics] this term only
4. (drug* or substance) and (illegal or misus* or abuse* or "problem use*" or "illicit use*" or addict* or dependen* or dependant or delinquency):ti or (drug* or substance) and (illegal or misus* or abuse* or "problem use*" or "illicit use*" or addict* or dependen* or dependant or delinquency):ab
5. MeSH descriptor: [Substance-Related Disorders] explode all trees
6. MeSH descriptor: [Drug Users] this term only
7. #1 or #2 or #3 or #4 or #5 or #6
8. (share* or sharing or integrat* or collabora* or continuity) near/2 (care or healthcare or service* or provision* or practice*):ti or (share* or sharing or integrat* or collabora* or continuity) near/2 (care or healthcare or service* or provision* or practice*):ab
9. #7 and #8

**Number of references retrieved and duplicates**

| **Database** | **Hits** |
| --- | --- |
| MEDLINE | 1092 |
| PsycINFO | 971 |
| CINAHL | 444 |
| CDSR | 1 |
| CENTRAL | 58 |
| DARE | 0 |
| HTA | 0 |
| **Total** | **2566** |
| **Duplicates** | **891** |
| **Total to screen** | **1675** |

1. VULNERABLE GROUPS

Database: MEDLINE

Host: Ovid

Data Parameters: 1946 to October Week 5 2013

Date Searched: 13/11/2013

Searcher: SB

Hits: 668

Strategy:

1. (vulnerable adj3 (group? or population? or people? or person? or individual?)).tw.
2. Vulnerable Populations/
3. or/1-2
4. (homeless* or vagrant? or vagabond? or "no fixed abode" or "no fixed address").tw.
5. (sleep* adj2 rough).tw.
6. homeless person/
7. or/4-6
8. ("asylum seeker*" or refugee? or "displaced person?" or emigre? or "illegal alien?" or deportee? or "stateless person?" or statelessness).tw.
9. Refugees/
10. or/8-9
11. (travel?er? or g?psy or g?psies or romany or romanies or roma).tw.
12. Gypsies/
13. or/11-12
14. (prostitut* or "sex work*" or "transactional sex*").tw.
15. Sex Workers/
16. or/14-15
17. (sex* adj3 (abus* or assualt* or crime? or offen?e? or traffic*)).tw.
18. (rape or molestation).tw.
19. Sex Offenses/
20. Rape/
21. or/17-20
22. ((domestic* or spousal or partner or marital or family) adj3 (abuse? or violence or rape)).tw.
23. (hono?r adj3 (violence or killing?)).tw.
24. Domestic Violence/
25. Spouse Abuse/
26. or/22-25
27. ("self-harm*" or "self-injur*" or "self-inflicted violence" or "non-suicidal self injur*" or "self-inflicted wound*" or "self-poison*").tw.
28. (suicide adj3 attempt*).tw.
29. Self-Injurious Behavior/
30. self-mutilation/
31. or/27-30
32. (learning adj3 (disabilit* or difficult* or disorder?)).tw.
33. exp learning disorder/
34. (dyslexi* or dyscalculi* or dysgraphi* or dyspraxi* or dysphasi* or aphasi* or "auditory processing disorder*" or "visual processing disorder*").tw.
35. exp Aphasia/
36. or/32-35
37. 3 or 7 or 10 or 13 or 16 or 21 or 26 or 31 or 36
38. ((share? or sharing or integrat* or collabora* or continuity) adj3 (care or healthcare or service? or provision? or practice?)).tw.
39. 37 and 38
40. limit 39 to english language

Database: PsycINFO

Host: Ovid

Data Parameters: 1806 to November Week 1 2013

Date Searched: 13/11/2013

Searcher: SB

Hits: 713

Strategy:

1. (vulnerable adj3 (group? or population? or people? or person? or individual?)).tw.
2. (homeless* or vagrant? or vagabond? or "no fixed abode" or "no fixed address").tw.
3. (sleep* adj2 rough).tw.
4. exp homeless/
5. or/2-4
6. ("asylum seeker*" or refugee? or "displaced person?" or emigre? or "illegal alien?" or deportee? or "stateless person?" or statelessness).tw.
7. Refugees/
8. or/6-7
9. (travel?er? or g?psy or g?psies or romany or romanies or roma).tw.
10. Gypsies/
11. or/9-10
12. (prostitut* or "sex work*" or "transactional sex*").tw.
13. prostitution/
14. or/12-13
15. (sex* adj3 (abus* or assualt* or crime? or offen?e? or traffic*)).tw.
16. (rape or molestation).tw.
17. Sex Offenses/
18. Rape/
19. or/15-18
20. ((domestic* or spousal or partner or marital or family) adj3 (abuse? or violence or rape)).tw.
21. (hono?r adj3 (violence or killing?)).tw.
22. Domestic Violence/
23. exp Partner Abuse/
24. or/20-23
25. ("self-harm*" or "self-injur*" or "self-inflicted violence" or "non-suicidal self injur*" or "self-inflicted wound*" or "self-poison*").tw.
26. (suicide adj3 attempt*).tw.
27. Self-Injurious Behavior/
28. self-mutilation/
29. or/25-28
30. (learning adj3 (disabilit* or difficult* or disorder?)).tw.
31. exp learning disorder/
32. (dyslexi* or dyscalculi* or dysgraphi* or dyspraxi* or dysphasi* or aphasi* or "auditory processing disorder*" or "visual processing disorder*").tw.
33. exp Aphasia/
34. dyslexia/
35. or/30-34
36. 1 or 5 or 8 or 11 or 14 or 19 or 24 or 29 or 35
37. ((share? or sharing or integrat* or collabora* or continuity) adj3 (care or healthcare or service? or provision? or practice?)).tw.
38. 36 and 37
39. limit 38 to english language

Database: CINAHL

Host: EBSCO

Data Parameters: n/a

Date Searched: 13/11/2013

Searcher: SB

Hits: 408

Strategy:

1. TI (vulnerable N2 (group* or population* or people* or person* or individual*)) OR AB (vulnerable N2 (group* or population* or people* or person* or individual*))
2. TI ( homeless* or vagrant* or vagabond* or "no fixed abode" or "no fixed address" ) OR AB ( homeless* or vagrant* or vagabond* or "no fixed abode" or "no fixed address" )
3. TI (sleep* N1 rough) OR AB( sleep* N1 rough)
4. (MH "Homeless Persons")
5. S2 OR S3 OR S4
6. TI ( "asylum seeker*" or refugee* or "displaced person*" or emigre* or "illegal alien*" or deportee* or "stateless person*" or statelessness ) OR AB ( "asylum seeker*" or refugee* or "displaced person*" or emigre* or "illegal alien*" or deportee* or "stateless person*" or statelessness )
7. (MH "Refugees")
8. S6 OR S7
9. TI ( traveler* or traveller* or g?psy or g?psies or romany or romanies or roma ) OR AB ( traveler* or traveller* or g?psy or g?psies or romany or romanies or roma )
10. (MH "Gypsies")
11. S9 OR S10
12. TI ( prostitut* or "sex work*" or "transactional sex*" ) OR AB ( prostitut* or "sex work*" or "transactional sex*" )
13. (MH "Prostitution")
14. S12 OR S13
15. TI ( sex* N2 (abus* or assualt* or crime* or offen?e* or traffic*) ) OR AB ( sex* N2 (abus* or assualt* or crime* or offen?e* or traffic*) )
16. TI ( rape or molestation ) OR AB ( rape or molestation )
17. (MH "Sexual Abuse")
18. (MH "Rape")
19. S15 OR S16 OR S17 OR S18
20. TI (domestic* or spousal or partner or marital or family) N2 (abuse* or violence or rape) OR AB (domestic* or spousal or partner or marital or family) N2 (abuse* or violence or rape)
21. TI (honor or honour) N2 (violence or killing*) OR AB (honor or honour) N2 (violence or killing*)
22. (MH "Domestic Violence")
23. (MH "Intimate Partner Violence")
24. S20 OR S21 OR S22 OR S23
25. TI ( "self-harm*" or "self-injur*" or "self-inflicted violence" or "non-suicidal self injur*" or "self-inflicted wound*" or "self-poison*" ) OR AB ( "self-harm*" or "self-injur*" or "self-inflicted violence" or "non-suicidal self injur*" or "self-inflicted wound*" or "self-poison*" )
26. TI suicide N2 attempt* OR AB suicide N2 attempt*
27. (MH "Self-Injurious Behavior")
28. S25 OR S26 OR S27
29. TI ( learning N2 (disabilit* or difficult* or disorder*) ) OR AB ( learning N2 (disabilit* or difficult* or disorder*) )
30. (MH "Learning Disorders+")
31. TI ( dyslexi* or dyscalculi* or dysgraphi* or dyspraxi* or dysphasi* or aphasi* or "auditory processing disorder*" or "visual processing disorder*" ) OR AB ( dyslexi* or dyscalculi* or dysgraphi* or dyspraxi* or dysphasi* or aphasi* or "auditory processing disorder*" or "visual processing disorder*" )
32. (MH "Aphasia")
33. S29 OR S30 OR S31 OR S32
34. S1 OR S5 OR S8 OR S11 OR S14 OR S19 OR S24 OR S28 OR S33
35. TI (share* or sharing or integrat* or collabora* or continuity) N2 (care or healthcare or service* or provision* or practice*) OR AB (share* or sharing or integrat* or collabora* or continuity) N2 (care or healthcare or service* or provision* or practice*)
36. S34 AND S35

Notes: Limited to English language

Database: Cochrane library

Host: Cochrane Collaboration

Data Parameters: CDSR and CENTRAL: Issue 10 of 12; DARE and HTA: Issue 4 of 4

Date Searched: 14/11/2013

Searcher: SB

Hits: CDSR=3; CENTRAL=19

Strategy:

1. vulnerable near/2 (group* or population* or people* or person* or individual*):ti or vulnerable near/2 (group* or population* or people* or person* or individual*):ab
2. MeSH descriptor: [Vulnerable Populations] this term only
3. #1 or #2
4. homeless* or vagrant* or vagabond* or "no fixed abode" or "no fixed address":ti or homeless* or vagrant* or vagabond* or "no fixed abode" or "no fixed address":ab
5. sleep* near/1 rough:ti or sleep* near/1 rough:ab
6. MeSH descriptor: [Homeless Persons] this term only
7. #4 or #5 or #6
8. "asylum seeker*" or refugee* or "displaced person*" or emigre* or "illegal alien*" or deportee* or "stateless person*" or statelessness:ti or "asylum seeker*" or refugee* or "displaced person*" or emigre* or "illegal alien*" or deportee* or "stateless person*" or statelessness:ab
9. MeSH descriptor: [Refugees] this term only
10. #8 or #9
11. traveller* or traveler* or g?psy or g?psies or romany or romanies or roma:ti or traveller* or traveler* or g?psy or g?psies or romany or romanies or roma:ab
12. MeSH descriptor: [Gypsies] this term only
13. #11 or #12
14. prostitut* or "sex work*" or "transactional sex*":ti or prostitut* or "sex work*" or "transactional sex*":ab
15. MeSH descriptor: [Sex Workers] this term only
16. #14 or #15
17. sex* near/2 (abus* or assualt* or crime* or offen?e* or traffic*):ti or sex* near/2 (abus* or assualt* or crime* or offen?e* or traffic*):ab
18. rape or molestation:ti or rape or molestation:ab
19. MeSH descriptor: [Sex Offenses] this term only
20. MeSH descriptor: [Rape] this term only
21. #17 or #18 or #19 or #20
22. (domestic* or spousal or partner or marital or family) near/2 (abuse? or violence or rape):ti or (domestic* or spousal or partner or marital or family) near/2 (abuse? or violence or rape):ab
23. (honor or honour) near/2 (violence or killing*):ti or (honor or honour) near/2 (violence or killing*):ab
24. MeSH descriptor: [Domestic Violence] this term only
25. MeSH descriptor: [Spouse Abuse] this term only
26. #22 or #23 or #24 or #25
27. "self-harm*" or "self-injur*" or "self-inflicted violence" or "non-suicidal self injur*" or "self-inflicted wound*" or "self-poison*":ti or "self-harm*" or "self-injur*" or "self-inflicted violence" or "non-suicidal self injur*" or "self-inflicted wound*" or "self-poison*":ab
28. suicide near/2 attempt*:ti or suicide near/2 attempt*:ab
29. MeSH descriptor: [Self-Injurious Behavior] this term only
30. MeSH descriptor: [Self Mutilation] this term only
31. #27 or #28 or #29 or #30
32. learning near/2 (disabilit* or difficult* or disorder*):ti or learning near/2 (disabilit* or difficult* or disorder*):ab
33. MeSH descriptor: [Learning Disorders] explode all trees
34. dyslexi* or dyscalculi* or dysgraphi* or dyspraxi* or dysphasi* or aphasi* or "auditory processing disorder*" or "visual processing disorder*":ti or dyslexi* or dyscalculi* or dysgraphi* or dyspraxi* or dysphasi* or aphasi* or "auditory processing disorder*" or "visual processing disorder*":ab
35. MeSH descriptor: [Aphasia] explode all trees
36. #32 or #33 or #34 or #35
37. #3 or #7 or #10 or #13 or #16 or #21 or #26 or #31 or #36
38. (share* or sharing or integrat* or collabora* or continuity) near/2 (care or healthcare or service* or provision* or practice*):ti or (share* or sharing or integrat* or collabora* or continuity) near/2 (care or healthcare or service* or provision* or practice*):ab
39. #37 and #38

Database: ASSIA and selected ProQuest Sociology Collection databases (IBSS, ProQuest Sociology, Social Services Abstracts, Sociological Abstracts)

Host: ProQuest

Data Parameters: n/a

Date Searched: 14/11/2013

Searcher: SB

Hits: 429

Strategy:

1. TI(vulnerable near/2 (group* or population* or people* or person* or individual*)) OR AB(vulnerable near/2 (group* or population* or people* or person* or individual*))
2. TI(homeless* or vagrant* or vagabond* or "no fixed abode" or "no fixed address") OR AB(homeless* or vagrant* or vagabond* or "no fixed abode" or "no fixed address") OR SU.EXACT("Homelessness") OR SU.EXACT.EXPLODE("Homeless people")
3. TI("asylum seeker*" or refugee* or "displaced person*" or emigre or "illegal alien*" or deportee or "stateless person" or statelessness) OR AB("asylum seeker*" or refugee* or "displaced person*" or emigre or "illegal alien*" or deportee or "stateless person" or statelessness) OR SU.EXACT("Asylum seekers") OR SU.EXACT.EXPLODE("Refugees")
4. TI(traveller* or traveler* or gipsy or gypsy or gipsies or gypsies or romany or romanies or roma) OR AB(traveller* or traveler* or gipsy or gypsy or gipsies or gypsies or romany or romanies or roma) OR SU.EXACT("Gypsies")
5. TI(prostitut* or "sex work*" or "transactional sex*") OR AB(prostitut* or "sex work*" or "transactional sex*") OR TI(sex* near/2 (abus* or assualt* or crime* or offender* or offence* or traffic*)) OR AB(sex* near/2 (abus* or assualt* or crime* or offender* or offence* or traffic*)) OR TI(rape or molestation) OR AB(rape or molestation) OR SU.EXACT.EXPLODE("Sex crimes") OR SU.EXACT.EXPLODE("Sex workers")
6. TI((domestic* or spousal or partner or marital or family) near/2 (abuse* or violence or rape)) OR AB((domestic* or spousal or partner or marital or family) near/2 (abuse* or violence or rape)) OR TI((honor or honour) near/2 (violence or killing*)) OR AB((honor or honour) near/2 (violence or killing*)) OR SU.EXACT.EXPLODE("Family Violence")
7. TI(self-harm* or self-injur* or "self-inflicted violence" or "non-suicidal self injur*" or "self-inflicted wound*" or "self-poison*") OR AB(self-harm* or self-injur* or "self-inflicted violence" or "non-suicidal self injur*" or "self-inflicted wound*" or "self-poison*") OR TI(suicide near/2 attempt*) OR AB(suicide near/2 attempt*) OR SU.EXACT("Self Destructive Behavior")
8. TI(learning near/2 (disabilit* or difficult* or disorder*)) OR AB(learning near/2 (disabilit* or difficult* or disorder*)) OR TI(dyslexi* or dyscalculi* or dysgraphi* or dyspraxi* or dysphasi* or aphasi* or "auditory processing disorder*" or "visual processing disorder*") OR AB(dyslexi* or dyscalculi* or dysgraphi* or dyspraxi* or dysphasi* or aphasi* or "auditory processing disorder*" or "visual processing disorder*") OR SU.EXACT.EXPLODE("Learning disabilities") OR SU.EXACT("Dyslexia")
9. TI((share* or sharing or integrat* or collabora* or continuity) near/2 (care or healthcare or service* or provision* or practice*)) OR AB((share* or sharing or integrat* or collabora* or continuity) near/2 (care or healthcare or service* or provision* or practice*))
10. (1 OR 2 OR 3 OR 4 OR 5 OR 6 OR 7 OR 8) AND 9

Database: Social Policy and Practice

Host: Ovid

Data Parameters: 201310

Date Searched: 14/11/2013

Searcher: SB

Hits: 409

Strategy:

1. (vulnerable adj3 (group? or population? or people? or person? or individual?)).tw.
2. (homeless* or vagrant? or vagabond? or "no fixed abode" or "no fixed address").tw.
3. (sleep* adj2 rough).tw.
4. 2 or 3
5. ("asylum seeker*" or refugee? or "displaced person?" or emigre? or "illegal alien?" or deportee? or "stateless person?" or statelessness).tw.
6. (travel?er? or g?psy or g?psies or romany or romanies or roma).tw.
7. (prostitut* or "sex work*" or "transactional sex*").tw.
8. (sex* adj3 (abus* or assualt* or crime? or offen?e? or traffic*)).tw.
9. (rape or molestation).tw.
10. or/7-9
11. ((domestic* or spousal or partner or marital or family) adj3 (abuse? or violence or rape)).tw.
12. (hono?r adj3 (violence or killing?)).tw.
13. 11 or 12
14. (self-harm* or self-injur* or "self-inflicted violence" or "non-suicidal self injur*" or "self-inflicted wound*" or "self-poison*").tw.
15. (suicide adj3 attempt*).tw.
16. 14 or 15
17. (learning adj3 (disabilit* or difficult* or disorder?)).tw.
18. (dyslexi* or dyscalculi* or dysgraphi* or dyspraxi* or dysphasi* or aphasi* or "auditory processing disorder*" or "visual processing disorder*").tw.
19. 17 or 18
20. 1 or 4 or 5 or 6 or 10 or 13 or 16 or 19
21. ((share? or sharing or integrat* or collabora* or continuity) adj3 (care or healthcare or service? or provision? or practice?)).tw.
22. 20 and 21

**Number of references retrieved and duplicates**

| **Database** | **Hits** |
| --- | --- |
| MEDLINE | 668 |
| PsycINFO | 713 |
| CINAHL | 408 |
| CDSR | 3 |
| CENTRAL | 19 |
| DARE | 0 |
| HTA | 0 |
| ProQuest databases | 429 |
| Social Policy and Practice | 409 |
| **Total** | **2649** |
| **Duplicates** | **939** |
| **Total to screen** | **1710** |

1. PRISONERS

Database: MEDLINE

Host: Ovid

Data Parameters: 1946 to October Week 5 2013

Date Searched: 13/11/2013

Searcher: SB

Hits: 192

Strategy:

1. (prisoner? or offender? or criminal? or inmate? or convict? or felon?).tw.
2. (ex-prisoner? or ex-offender? or ex-criminal? or ex-inmate? or ex-convict? or ex-felon?).tw.
3. prisoners/
4. or/1-3
5. ((share? or sharing or integrat* or collabora* or continuity) adj3 (care or healthcare or service? or provision? or practice?)).tw.
6. 4 and 5
7. ((indeterminate or indefinite) adj3 (sentenc* or imprisonment)).tw.
8. ("imprisonment for public protection" or "imprisonment for life").tw.
9. ("through the gate" or "in-reach" or inreach).tw.
10. or/7-9
11. 4 and 10
12. 6 or 11
13. limit 12 to english language

Database: MEDLINE-in-Process
Host: Ovid
Data Parameters: November 12, 2013
Date Searched: 13/11/2013
Searcher: SB
Hits: 22
Strategy:

1. (prisoner? or offender? or criminal? or inmate? or convict? or felon?).tw.
2. (ex-prisoner? or ex-offender? or ex-criminal? or ex-inmate? or ex-convict? or ex-felon?).tw.
3. or/1-2
4. ((share? or sharing or integrat* or collabora* or continuity) adj3 (care or healthcare or service? or provision? or practice?)).tw.
5. 3 and 4
6. ((indeterminate or indefinite) adj3 (sentenc* or imprisonment)).tw.
7. ("imprisonment for public protection" or "imprisonment for life").tw.
8. ("through the gate" or "in-reach" or inreach).tw.
9. or/6-8
10. 3 and 9
11. 5 or 10

Database: PsycINFO

Host: Ovid

Data Parameters: 1806 to November Week 1 2013

Date Searched: 13/11/2013

Searcher: SB

Hits: 372

Strategy:

1. (prisoner? or offender? or criminal? or inmate? or convict? or felon?).tw.
2. (ex-prisoner? or ex-offender? or ex-criminal? or ex-inmate? or ex-convict? or ex-felon?).tw.
3. prisoners/
4. exp criminals/
5. or/1-4
6. ((share? or sharing or integrat* or collabora* or continuity) adj3 (care or healthcare or service? or provision? or practice?)).tw.
7. 5 and 6
8. ((indeterminate or indefinite) adj3 (sentenc* or imprisonment)).tw.
9. ("imprisonment for public protection" or "imprisonment for life").tw.
10. ("through the gate" or "in-reach" or inreach).tw.
11. or/8-10
12. 5 and 11
13. 7 or 12
14. limit 13 to english language

Database: CINAHL

Host: EBSCO

Data Parameters: n/a

Date Searched: 14/11/13

Searcher: SB

Hits: 125

Strategy:

1. TI (prisoner* or offender* or criminal* or inmate* or convict* or felon*) OR AB (prisoner* or offender* or criminal* or inmate* or convict* or felon*)
2. TI ( ex-prisoner* or ex-offender* or ex-criminal* or ex-inmate* or ex-convict* or ex-felon* ) OR AB ( ex-prisoner* or ex-offender* or ex-criminal* or ex-inmate* or ex-convict* or ex-felon* )
3. (MH "Prisoners")
4. S1 OR S2 OR S3
5. TI (share* or sharing or integrat* or collabora* or continuity) N2 (care or healthcare or service* or provision* or practice*) OR AB (share* or sharing or integrat* or collabora* or continuity) N2 (care or healthcare or service* or provision* or practice*)
6. S4 AND S5
7. TI (indeterminate or indefinite) N2 (sentenc* or imprisonment) OR AB (indeterminate or indefinite) N2 (sentenc* or imprisonment)
8. TI ( "imprisonment for public protection" or "imprisonment for life" ) OR AB ( "imprisonment for public protection" or "imprisonment for life" )
9. TI ( "through the gate" or "in-reach" or inreach ) OR AB ( "through the gate" or "in-reach" or inreach )
10. S7 OR S8 OR S9
11. S4 AND S10
12. S6 OR S11

Notes: Limited to English language.

Database: Cochrane

Host: Cochrane Collaboration

Data Parameters: CDSR and CENTRAL: Issue 10 of 12; DARE and HTA: Issue 4 of 4

Date Searched: 14/11/2013

Searcher: SB

Hits: CENTRAL=4

Strategy:

1. prisoner* or offender* or criminal* or inmate* or convict* or felon*:ti or prisoner* or offender* or criminal* or inmate* or convict* or felon*:ab
2. ex-prisoner* or ex-offender* or ex-criminal*or ex-inmate* or ex-convict* or ex-felon*:ti or ex-prisoner* or ex-offender* or ex-criminal*or ex-inmate* or ex-convict* or ex-felon*:ab
3. MeSH descriptor: [Prisoners] this term only
4. #1 or #2 or #3
5. (share* or sharing or integrat* or collabora* or continuity) near/2 (care or healthcare or service* or provision* or practice*):ti or (share* or sharing or integrat* or collabora* or continuity) near/2 (care or healthcare or service* or provision* or practice*):ab
6. #4 and #5
7. (indeterminate or indefinite) near/2 (sentenc* or imprisonment):ti or (indeterminate or indefinite) near/2 (sentenc* or imprisonment):ab
8. ("imprisonment for public protection" or "imprisonment for life"):ti or ("imprisonment for public protection" or "imprisonment for life"):ab
9. ("through the gate" or "in-reach" or inreach):ti or ("through the gate" or "in-reach" or inreach):ab
10. #7 or #8 or #9
11. #4 and #10
12. #6 or #11

Database: ASSIA and selected ProQuest Sociology Collection databases (IBSS, ProQuest Sociology, Social Services Abstracts, Sociological Abstracts)

Host: ProQuest

Data Parameters: n/a

Date Searched: 14/11/2013

Searcher: SB

Hits: 276

Strategy:

1. TI(prisoner* or offender* or criminal* or inmate* or convict* or felon*) OR AB(prisoner* or offender* or criminal* or inmate* or convict* or felon*) OR TI(ex-prisoner* or ex-offender* or ex-criminal* or ex-inmate* or ex-convict* or ex-felon*) OR AB(ex-prisoner* or ex-offender* or ex-criminal* or ex-inmate* or ex-convict* or ex-felon*)
2. SU.EXACT("Prisoners")
3. TI((share* or sharing or integrat* or collabora* or continuity) near/2 (care or healthcare or service* or provision* or practice*)) OR AB((share* or sharing or integrat* or collabora* or continuity) near/2 (care or healthcare or service* or provision* or practice*))
4. TI((indeterminate or indefinite) near/2 (sentenc* or imprisonment)) OR AB((indeterminate or indefinite) near/2 (sentenc* or imprisonment))
5. TI("imprisonment for public protection" or "imprisonment for life") OR AB("imprisonment for public protection" or "imprisonment for life")
6. TI("through the gate" or "in-reach" or inreach) OR AB("through the gate" or "in-reach" or inreach)
7. (1 OR 2) AND 3
8. (4 OR 5 OR 6) AND (1 OR 2)
9. 7 OR 8

Database: Social Policy and Practice

Host: Ovid

Data Parameters: 201310

Date Searched: 14/11/2013

Searcher: SB

Hits: 160

Strategy:

1. (prisoner? or offender? or criminal? or inmate? or convict? or felon?).tw.
2. (ex-prisoner? or ex-offender? or ex-criminal? or ex-inmate? or ex-convict? or ex-felon?).tw.
3. or/1-2
4. ((share? or sharing or integrat* or collabora* or continuity) adj3 (care or healthcare or service? or provision? or practice?)).tw.
5. 3 and 4
6. ((indeterminate or indefinite) adj3 (sentenc* or imprisonment)).tw.
7. ("imprisonment for public protection" or "imprisonment for life").tw.
8. ("through the gate" or "in-reach" or inreach).tw.
9. or/6-8
10. 3 and 9
11. 5 or 10

Database: Campbell library

Host: Campbell Collaboration

Data Parameters: n/a

Date Searched: 14/11/2013

Searcher: SB

Hits: 7

Strategy:

1. "shared care" or "shared healthcare" or "shared services" or "or shared service" or "shared provisions" or "shared provision" or "shared practices" or "shared practice"
2. "sharing care" or "sharing healthcare" or "sharing services" or "sharing service" or "sharing provisions" or "sharing provision" or "sharing practices" or "sharing practice"
3. "integrated care" or "integrated healthcare" or "integrated services" or "integrated service" or "integrated provisions" or "integrated provision" or "integrated practices" or "integrated practice"
4. "collaborative care" or "collaborative healthcare" or "collaborative services" or "collaborative service" or "collaborative provisions" or "collaborative provision" or "collaborative practices" or "collaborative practice"
5. "continuity care" or "continuity healthcare" or "continuity services" or "continuity service" or "continuity provisions" or "continuity provision" or "continuity practices" or "continuity practice"

Notes: Searched in All Text and lines combined using OR.

**Number of references retrieved and duplicates**

| **Database** | **Hits** |
| --- | --- |
| MEDLINE | 192 |
| MEDLINE-inProcess | 22 |
| PsycINFO | 372 |
| CINAHL | 125 |
| CDSR | 0 |
| CENTRAL | 4 |
| DARE | 0 |
| HTA | 0 |
| ProQuest databases | 276 |
| Social Policy and Practice | 160 |
| Campbell Library | 7 |
| **Total** | **1158** |
| **Duplicates** | **353** |
| **Total to screen** | **805** |
