# Supplementary material for: Using realist review to inform intervention development: methodological illustration and conceptual platform for collaborative care in offender mental health
Source: Implement Sci. 2015 Sep 28;10:134. doi: 10.1186/s13012-015-0321-2 (PMC4584430; doi:10.1186/s13012-015-0321-2)
Supplement: Additional file 2: — Screening tools. [file 13012_2015_321_MOESM2_ESM.docx]

**Supplementary File 2: Screening Tools**

**Abstract and Full-Text Classification Tool**

| **Author/Citation/ ID** |  | | | |
| --- | --- | --- | --- | --- |
| **Activity** | **Sub-section** | **Description** | **Code** |  |
| Reviewer ID |  | Who is screening? | MP/ SB/ CT |  |
| How source was located |  | Call for evidence (Twitter, JISCMail, IDG) | CFE |  |
|  |  | Handsearching | HS |  |
|  |  | Website | Web |  |
|  |  | Citation chasing | CC |  |
|  |  | Table of contents alerts | ToC |  |
|  |  | Browsing | Brws |  |
|  |  | Database | DB |  |
| **ON BASIS OF ABSTRACTS** | | | | |
| Inc./Exc. Decision | Does the source contain or test programme theories about any of the components in the provisional Theoretical Intervention Model or Delivery Platform? | | | |
|  |  | Include | IncAb |  |
|  |  | Exclude | ExcAb |  |
| Classification | 1. Field of practice to which source predominantly refers (code one only) | Prisoners/offenders | AbCJ |  |
|  |  | Vulnerable groups other than prisoners/offenders | AbVG |  |
|  |  | Mental health | AbMH |  |
|  |  | Other healthcare | AbOH |  |
|  | 2. Type of source (code one only) | Policy document | AbPD |  |
|  |  | Viewpoint/editorial | AbVE |  |
|  |  | Primary research | AbPR |  |
|  |  | Review | AbRev |  |
|  | 3. Areas covered (code all that apply) | Service organisation | AbSO |  |
|  |  | Practitioner characteristics | AbPC |  |
|  |  | Care interventions | AbCI |  |
|  |  | Case management | AbCM |  |
|  |  | Integration of and liaison between teams | AbILT |  |
|  |  | Individuals’ contexts | AbInCx |  |
|  | 4. Sources of PTs (code all that apply) | Prisoners/offenders | AbPrOf |  |
|  |  | Significant others of prisoners/offenders | AbSOPO |  |
|  |  | Practitioners | AbPrc |  |
|  |  | Academics | AbAc |  |
|  |  | Policy makers | AbPM |  |
|  |  | Other | AbPTO |  |
|  | 5. Aspect of review applicable to (code all that apply) | Theoretical Intervention Model | AbTIM |  |
|  |  | Delivery Platform | AbDP |  |
|  | 6. ‘Richness’ - see criteria table (code one only) | Conceptually-rich | AbCR |  |
|  |  | Thick | AbTk |  |
|  |  | Thin | AbTn |  |
| If unsure how to code |  | To discuss with review team (note which aspects) | AbDsc |  |
|  |  |  |  |  |
|  |  |  |  |  |
|  |  |  |  |  |
|  |  |  |  |  |
|  |  |  |  |  |
|  |  |  |  |  |
| **Author/Citation/ ID** |  | | | |
| **Activity** | **Sub-section** | **Description** | **Code** |  |
| **ON BASIS OF FULL-TEXT** | | | | |
| Inc./Exc. Decision | Does the source contain or test programme theories about any of the components in the provisional TIM or DP? | | | |
|  |  | Include | IncFT |  |
|  |  | Exclude | ExcFT |  |
| Classification | 1. Field of practice to which source predominantly refers (code one only) | Prisoners/offenders | FTCJ |  |
|  |  | Vulnerable groups other than prisoners/offenders | FTVG |  |
|  |  | Mental health | FTMH |  |
|  |  | Other healthcare | FTOH |  |
|  | 2. Type of source (code one only) | Policy document | FTPD |  |
|  |  | Viewpoint/editorial | FTVE |  |
|  |  | Primary research | FTPR |  |
|  |  | Review | FTRev |  |
|  |  | Manual | FTMan |  |
|  | 3. Areas covered (code all that apply) | Service organisation | FTSO |  |
|  |  | Practitioner characteristics | FTPC |  |
|  |  | Care interventions | FTCI |  |
|  |  | Case management | FTCM |  |
|  |  | Integration of and liaison between teams | FTILT |  |
|  |  | Individuals’ contexts | FTInCx |  |
|  | 4. Sources of PTs (code all that apply) | Prisoners/offenders | FTPrOf |  |
|  |  | Significant others of prisoners/offenders | FTSOPO |  |
|  |  | Practitioners | FTPrc |  |
|  |  | Academics | FTAc |  |
|  |  | Policy makers | FTPM |  |
|  |  | Other | FTPTO |  |
|  | 5. Aspect of review applicable to (code all that apply) | Theoretical Intervention Model | FTTIM |  |
|  |  | Delivery Platform | FTDP |  |
|  | 6. ‘Richness’ - see criteria table (code one only) | Conceptually-rich | FTCR |  |
|  |  | Thick | FTTk |  |
|  |  | Thin | FTTn |  |
| If unsure how to code |  | To discuss with review team (note which aspects) | FTDsc |  |

**Use notes field in EndNote for all codes – and *use separate line for each code***

**Criteria for assessing conceptual-richness of sources**

| **Conceptually-rich** | **‘Thick’ description** | **‘Thin’ description’** |
| --- | --- | --- |
| Theories or concepts grounded in a body of literature | Consideration of context | Limited or no consideration of the context |
|  | Discussion of the differences between what people intended and what actually happened | Limited or no discussion of the differences between what people intended and what actually happened |
| Likely language (but not limited to these):  Terms - theory, concept, conceptual model  Verbs - integrate  Topics - interaction of [conceptualised] phenomena within a [conceptualised] system | Likely language (but not limited to these):  Terms - model, process or function  Verbs - investigate, describes, explains  Topics - experiences |  |

‘Thick’ and ‘thin’ criteria based on [1]

1. Roen K, Arai L, Roberts H, Popay J. Extending systematic reviews to include evidence on implementation: methodological work on a review of community-based initiatives to prevent injuries. Social Science & Medicine. 2006;63:1060-71.
