# Supplementary material for: Using realist review to inform intervention development: methodological illustration and conceptual platform for collaborative care in offender mental health
Source: Implement Sci. 2015 Sep 28;10:134. doi: 10.1186/s13012-015-0321-2 (PMC4584430; doi:10.1186/s13012-015-0321-2)
Supplement: Additional file 3: — Explanatory accounts. [file 13012_2015_321_MOESM3_ESM.docx]

**Supplementary File 3: Explanatory Account (EA) Table**

| **EA#** | **‘Raw data’ (citation ID#)** | **Expressed as an explanatory account** | **Whose EA?** |
| --- | --- | --- | --- |
| 1 | Byng et al. (2012), p.181 (#931) | *If ‘…*offenders, healthcare practitioners, and criminal justice staff can co-create and implement a resettlement package’  *then* CMHPs in offenders will be better addressed. The components of such a package would be:  - trusting relationships  - integration of social inclusion and health  - organisational arrangements that prioritise communication and collaborative care | Academic |
| 2 | Byng et al. (2012), p.181 (#931) | *If* the relationship between practitioners (healthcare and CJS) and offenders is central to ‘…generating emotional well-being, resettlement and reduced offending’  *then* ‘…the relatively stable context of probation and prison [is key] to exploiting the potential for positive feedback cycles’ | Academic |
| 3 | Byng et al. (2012), p.181 (#931) | *If* primary care services could also ‘…perform the liaison and diversion function in police and courts settings’  *then* CMHPs in offenders will be better addressed. | Academic |
| 4 | Byng et al. (2012), p.193 (#931) | *If* IAPT services ‘…allowed access for those with some substance misuse and with difficult personality traits’  *then* CMHPs in offenders will be better addressed. | Academic |
| 5 | Byng et al. (2012), p.193 (#931) | *If* mental health care is ‘…embedded within non-health organisations (e.g. employment, training, addressing relationships)  *then* CMHPs in offenders will be better addressed. | Academic |
| 6 | Byng et al. (2012), p.193 (#931) | *If* ‘…in-reach services in prison were re-shaped to make better use of limited resources and be more primary care focused’  *then* CMHPs in offenders will be better addressed. | Academic |
| 7 | Byng et al. (2012), p.193 (#931) | *If* ‘…mental health promotion activities were collaboratively arranged between probation and prison resettlement and healthcare [and] focused on recovery rather than deficits’  *then* attendance would improve. | Academic |
| 8 | Peer Researcher Group Meeting, September 2013 | *If* men in prison know that their family is receiving support, counselling (esp. children) financial advice and there is a liaison person between family and offender in prison  *then* offender mental health will be less negatively impacted by being imprisoned (‘would relieve huge amount of pressure’). | Peer Researcher |
| 9 | Peer Researcher Group Meeting, September 2013 | *If* men in prison have social support from other men in prison  *then* they feel more safe and comfortable in themselves and their surroundings  *then* their mental health is less negatively affected by being imprisoned. | Peer Researcher |
| 10 | Peer Researcher Group Meeting, September 2013 | *If* men in prison have social support from other men in prison  *then* they get more information about how to get what they need inside prison and then their mental health is less negatively affected by being imprisoned (and able to get what they need to get by) | Peer Researcher |
| 11 | Peer Researcher Group Meeting, September 2013 | *If* men being released from prison who ‘have nothing’ on the outside have had their benefits made ready for their release (e.g. by job centre visiting men prior to release)  *then* they can financially support themselves as soon as they leave prison  *and then* they are less likely to reoffend. | Peer Researcher |
| 12 | Peer Researcher Group Meeting, September 2013 | *If* men in prison are given information about services available to them during their induction (not in the form of leaflets but rather word of mouth) by engaged and informed prison officers  *then* a barrier to service use (not knowing about services available inside) will be removed. | Peer Researcher |
| 13 | Peer Researcher Group Meeting, September 2013 | *If* men in prison are treated as individuals with the need to keep in communication with their families, and this is taken as a priority  *then* this takes away further disempowerment that their circumstances ascribe and makes them more likely to adhere to rules (ie not smuggling in mobile phones so that continued communication can be kept). | Peer Researcher |
| 14 | Peer Researcher Individual Meeting, October 2013 | *If* men in Prison believe that their sentence will be affected by being sent to Hospital (as it is believed that time spent in hospital doesn’t count on sentence)  *then* they will be reluctant to present themselves as having a mental health issue to Prison or healthcare staff  *then* their mental health will be negatively affected (As they are not receiving treatment) | Peer Researcher |
| 15 | Richard Byng, Personal Communication, October 2013 | *If* care making up the intervention for the individual is based around a choice of what the individual wants  *Then* individuals’ are motivated and engaged in their care | Study Group; #703; #714; 824; #825 |
| 16 | Richard Byng, Personal Communication, October 2013 | *If* care generated by the intervention is designed to utilise the strengths and creativity of an individual (rather than them being a passive object of intervention)  *Then* wellbeing is improved | Study Group; #703; #714; 824; #825 |
| 17 | Richard Byng, Personal Communication, October 2013 | *If a broad spectrum of already existing health and non-health services are mobilised by the intervention towards a range of* ideographic goals  *Then* wellbeing will be further increased | Study Group |
| 18 | Richard Byng, Personal Communication, October 2013 | *If* ideographic goals, selected as important to the offender, are the focus of care (rather than just the ‘known’ average needs of care group)  *Then* motivation/energy/agency of offenders taking part in intervention is mobilised. | Study Group; #703; #714; #824; #825 |
| 19 | Quinn et al. (2012) p.13 (#329) | *If* offender assessment systems do not include structured sections to assess healthcare needs  *Then* offenders’ healthcare needs risk remaining unidentified and unmonitored | Academic |
| 20 | Quinn et al. (2012) p.13 (#329) | ‘When offenders are in crisis and in need of support… they are less likely to access healthcare services of their own volition.’ | Academic |
| 21 | Quinn et al. (2012) p.13 (#329) | Probation officers’ ability to refer to services is limited by their sole source of information, i.e. their experiential knowledge of agencies and their remit | Academic |
| 22 | Quinn et al. (2012) p.14 (#329) | *If* services refer to, and involve, other services in a purely instrumental sense  *Then* a downward spiral of non-collaborative working is perpetuated | Academic |
| 23 | Quinn et al. (2012) p.14 (#329) | *If* health services are located where offenders are already visiting for their criminal justice requirements  *Then* they are more likely to use them | Academic |
| 24 | Quinn et al. (2012) p.14 (#329) | *If* health services in the community are located within probation services and staffed by probation-specific health professionals  *Then* offenders perceive less risk of being stigmatised  *and* offender uptake of services is improved | Academic |
| 25 | Quinn et al. (2012) p.15 (#329) | Practitioner attitude and understanding of wider issues affecting an offender is more important than specialist knowledge or experience | Practitioner |
| 26 | Quinn et al. (2012) p.22 (#329) | *If* services in prisons were ‘configured in such a way as to encourage offenders to take more responsibility for themselves’  *Then* offenders’ would be in a better position to make good use of existing community services upon release | Practitioner |
| 27 | Quinn et al. (2012) p.26 (#329) | *If* referrers (e.g. Police, GPs) to community services  - understand the referral process  - have access to documentation to make the referral  - perceive that referrals will be welcomed, not rejected  *Then* collaborative working relationships are fostered  *and* more offenders are appropriately referred to community health services | Practitioner |
| 28 | Quinn et al. (2012) p.27 (#329) | *If* practitioners understand the roles of practitioners working in different organisations  *Then* collaborative working is increased | Practitioner |
| 29 | Quinn et al. (2012) p.27 (#329) | *If* non-health professionals who work with offenders do not have referral powers in relation to CMHPs  *Then* access to health professionals is impeded and CMHPs risk remaining unaddressed | Practitioner |
| 30 | Feron et al. (2008) p.149 (#27) | Where ‘tedium, anxiety, idleness, loneliness, and isolation from relatives or other sources of support [characterise prison life]… prisoners view the time they spend in prison as a punishment [rather than rehabilitation]… and a feeling of not existing in society’ | Offender |
| 31 | Feron et al. (2008) p.151 (#27) | *If* offenders do not trust healthcare staff  *Then either* health-seeking behaviour is discouraged  *or* offenders pursue rights and status through ‘non-justified’ health-seeking behaviour (a ‘bargaining strategy’) | Academic |
| 32 | Tompkins et al. (2007) p.199 (#37) | *If* substitute drugs (e.g. methadone) are not prescribed/provided for users on release  *Then* offenders’ drug withdrawal is put at risk and offenders feel ‘let down’ by the system | Offender |
| 33 | De Viggiani (2006) p.86-87 (#38) | *As* prisons are closed environments into which existing social and structural determinants of health are imported and concentrated  *and* the resulting competitive, macho, violent, homophobic and racist environment decreases physical and mental health and inhibits help-seeking behaviours  *Then* improvement will only occur through addressing these ‘upstream’ (outside of prison) determinants | Academic |
| 34 | Peer Researcher Group Meeting, October 2013 | *If* men in Prison that are suffering from withdrawal symptoms are taught yoga so that they can do it individually  *then* when they are withdrawing from drugs they would have an active way of exercising and relaxing from their own cell  *then* their mental health will be less negatively affected as they have a tool to use to both exercise and relax. | Peer Researcher |
| 35 | Peer Researcher Group Meeting, October 2013 | *If* services (such as the job centre) employed ex-offenders to advise men that have just been released from Prison  *then* men that have just been released would not feel as intimidated going to services (job centre)  *and* they would have more belief that services have the relevant skills and experience to advise them | Peer Researcher |
| 36 | Reed et al. (2009) p.22 (#54) | (context)  Longer-term prisoners tend to use more emotion-focused, and less problem-focused, coping strategies.  Shorter-term prisoners are also more likely to seek social support. | Academic |
| 37 | Reed et al. (2009) p.22 (#54) | *If* a shorter-term prison sentence provides a concentrated time and space in which a ‘relatively stable’ social network within prison can be built  *then* short-term prisoners have a significant ‘advantage’ over long-term prisoners for whom fellow inmates may be constantly changing. | Academic |
| 38 | Reed et al. (2009) p.23 (#54) | *If* social support networks can be ‘mobilised’ both within and outside of prison  *then* the negative psychological impact of stress/prison will be decreased. | Academic |
| 39 | F. M. Smith and Marshall (2007) p.166 (#67) | *Where* service users are vulnerable and/or stigmatised, keeping the same key worker:  - builds on the rapport that fragile service users find hard to develop  - reduces the need to recount traumatic ‘life stories’  - maintains trust | Service user |
| 40 | Smith & Marshall (2007) p.167 (#67) | *Where* life is perceived as out-of-control and desperate, service users can feel ready to engage with services only infrequently and/or at short notice  *so*  *If* services do not/cannot respond very quickly  *then* the opportunity to engage is lost, perhaps for a significant period of time.  (Context: substance use, where substances may provide a ‘quick fix’ in place of the more prolonged engagement with services) | Service user |
| 41 | Masterson and Owen (2006) (#76) | Addressing social (structural) issues and individual empowerment work in tandem – doing one without the other does not improve mental health service users’ well-being. | Academic |
| 42 | Emlyn-Jones (2007) p.240 (#79) | Necessary components of an intervention for facilitating behaviour change around substance use by offenders:  - Skills: ‘well-trained, well-supported staff to engage, advocate and enable’  - Timing: ‘the relationship begins in prison and continues without a break until the goals are achieved’  - Intensity of input  - Relationships ‘between worker and service user built on mutual respect, empathy and an understanding that the individual is an expert in their own life; the solutions lie in their strengths and preferred futures, not ours’ | Other |
| 43 | Lindqvist (2007) p.246 (#80) | *If* there is informed and networking leadership, elimination of financial factors that inhibit clinical management, and practitioners have a positive attitude to interdisciplinary work  *then* collaboration between mental health and social services ‘works well’ | Academic |
| 44 | Rutherford et al. (2008) p.265 (#83) | *If* access to mental health care, accommodation and finding and sustaining employment can be maximised  *then* the likelihood of successful resettlement is increased. | Other |
| 45 | Klimidis et al. (2007) p.474 (#118) | One or more of the following may explain lack of engagement with mental health services:  - (potential) service users’ perception of the symptoms of mental illness as something other than psychological  - (lack of) practical knowledge about services  - attitude towards mental health  - extent of ‘tolerance’ of mental illness, e.g. stoicism | Academic |
| 46 | Verhaeghe et al. (2008) p.215 (#120) | Stigma impacts negatively on self-esteem *except* where commonality of experience is found within a group, in which case the functioning of peer support substantially moderates this negative impact. | Academic |
| 47 | Schön et al. (2009) p.340 (#122) | *If* an individual’s (positive) personal characteristics can be ‘harnessed’, and they perceive some level of control over their own life  *then* recovery from mental illness is facilitated, | Other |
| 48 | Schon et al. (2009) p.340 (#122) | *If* ‘meaning making’ (understanding) mental illness and its consequences for an individual’s life is an essential part of recovery  *then* ‘facilitative activities’ for self-expression (e.g. diary, painting)  *and* avoiding activities that cause stress and illness  are both important for recovery. | Other |
| 49 | Schon et al. (2009) p.341 (#122) | In mental health care, *where* professionals ‘show an interest and involvement in an individual over and beyond the patient and the diagnosis’, had helped to ‘make sense of what was important for the patient’, and ‘showed an interest in patients’ experiences and knowledge  *then* service users feel valued. | Other |
| 50 | Schon et al. (2009) p.344 (#122) | A social network (family, friends) provides practical and emotional support that is crucial for recovery from mental illness.  Family members’ contribution to recovery from mental illness can be distinct because of the depth and complexity of their knowledge about an individual. | Other |
| 51 | Collins & Barker (2009) p.378 (#123) | *If* vulnerable (homeless) people mistrust formal sources of help, and/or believe that asking for help exposes one to disappointment and exploitation  *then* services will not be accessed and self-sufficiency and independence will be valued above help-seeking. | Other |
| 52 | Collins & Barker (2009) p.378 (#123) | *If* young homeless people initially have high expectations about community service provision which they then find are unrealistic  *then* a negative cycle of disappointment and disengagement from services is initiated. | Other |
| 53 | Collins & Barker (2009) p.379 (#123) | *If* young homeless people have a deep mistrust of services from their experiences in childhood  *then* their trust in, and engagement with, services in adult life is severely limited. | Other |
| 54 | Collins & Barker (2009) p.379 (#123) | Young homeless people place a high value on the quality of their relationships with practitioners – the components of this relationship being:  - ‘they really do care’  - empathic and non-judgemental (perhaps strengthened if practitioner has lived experience of issues)  - an ear that genuinely listens  - trust, for example demonstrated through actions taken to address the (practical) priorities of the service user. | Other |
| 55 | Kinderman (2009) p.465-466 (#124) | Biological, social and circumstantial factors (that can lead to psychological and social problems) are mediated by psychological processes  *therefore* addressing these psychological processes (which may manifest as disillusionment, lack of personal efficacy and sense of agency) should play a central role in mental health interventions (e.g. to improve self-concept, interpersonal relationships, and motivation) | Academic |
| 56 | Windell & Norman (2012) p.495 (#132) | *If* the distinct contributions of those within a person’s social network (partner, family, friends, friend ‘within the psychiatric community’, work colleagues) can be harnessed  *then* a sense of social relatedness and value is provided  *and* expectations for recovery are enhanced  *and*, ultimately, prospects for recovery are improved. | Other |
| 57 | Windell & Norman (2012) p.496 (#132) | ‘Personal investment’ by practitioners in mental health service users, through ‘getting to know the person and his or her strengths as well as challenges, personal preferences and priorities’, can ‘foster a sense of trust and of being valued’ which is vital for promoting recovery.  (other, less key, factors may include: constancy and accessibility of support, messages of hope and positive expectations for recovery, nonjudgmental attitudes, multiple service options, personalised treatment and support). | Other |
| 58 | Windell & Norman (2012) p.496 (#132) | *If* mental health service users can take part in activities that provide a sense of meaning, purpose, and worth, or activities that have social status  *then* recovery from mental illness is enhanced. | Other |
| 59 | Windell & Norman (2012) p.497 (#132) | Effects of stigma on recovery from mental illness:  - interferes with acceptance of being ill and needing treatment  - reduced sense of control, self-esteem, and confidence  - prevents social inclusion  - (at worst) demoralises individuals completely | Other |
| 60 | Buetow (2007) p.183 (#3) | *If* health professionals feel committed to goals, understand what they need to do to achieve these goals, and feel competent to act as the goals require  *Then* they will be motivated to improve their performance  (Theory of Goal Setting; GST) | Academic |
| 61 | Buetow (2007) p.184 (#3) | *If* health professionals’ efforts are channelled towards policy-defined goals through motivating effects of money  *Then* goal-related performance will be improved  (GST ‘business case’) | Academic |
| 62 | Buetow (2007) p.184 (#3) | *If* health professionals’ goals have intrinsic ‘moral’ rewards or incentives, such as pride,  *Then* they will be more motivated to improve performance towards a goal  (GST ‘pride case’) | Academic |
| 63 | Buetow (2007) p.184 (#3) | *If* there is a good ‘fit’ between health professional and their environment (i.e organisational culture) which sets opportunities for them to express their personality, values, needs, and goals,  *Then* they will be motivated at work | Academic |
| 64 | Buetow (2007) p.185 (#3) | *If* policies are responsive to the complex interplay of individuals’ needs, values, personality traits, cognition, affect, environment, & behaviour  *Then* health professionals will be more motivated | Academic |
| 65 | Buetow (2007) p.185 (#3) | *If* health professionals’ basic needs for competence, autonomy, and relatedness are met  *Then* they will be more motivated | Academic |
| 66 | Davies et al. (2007) p. 129 (#5) | *If* health professionals have limited knowledge and understanding of current concepts and methods of quality improvement, and if there are differing definitions between health professionals regarding what high quality care is, and they believe that local care is of a high quality  *Then* they will not engage in quality improvement of care | Academic |
| 67 | Davies et al (2007) p. 129 (#5) | *If* quality improvement involves unfamiliar methodologies or unwelcome messages (explicit or implicit; such as ‘local care is of poor quality’)  *Then* this will be a significant barrier to engagement in care quality improvement by professionals | Academic |
| 68 | Davies et al (2007) p. 129 (#5) | *If* health professionals believe quality initiatives are a waste of scarce personal and organisational resources  *Then* they will not engage | Academic |
| 69 | Davies et al (2007) p. 129 (#5) | *If* quality improvement initiatives require, implicitly or explicitly, health professionals to pay greater attention to patient experience and patient engagement in ways that are at odds with their prior training, socialisation, and customary practice  *Then* they will not engage | Academic |
| 70 | Davies et al (2007) p. 130 (#5) | *If* quality improvement requires changes in the traditional division of labour between health professionals and may require some professionals to give up aspects of their autonomy, leadership, or status  *Then* there will be low engagement | Academic |
| 71 | Davies et al (2007) p. 130 (#5) | *If* quality improvement involves finding creative ways to mobilise health professionals’ knowledge and experience to change services  *Then* engagement in quality improvement will be improved | Academic |
| 72 | Caulfield & Twort (2012) p. 12 (#61) | *If* there is a lack of understanding between the prison service and the NHS regarding how the other organisation operates  *Then* this will act as a barrier to partnership working between prison service and NHS | Academic |
| 73 | Caulfield & Twort (2012) p. 13 (#61) | *If* there is investment in staff training on new practices and processes  *Then* prison staff experiences of change will be more positive | Academic |
| 74 | Marks et al. (2006) p.49 (#31) | *If* health practitioners in prison receive practical and ethical guidance to help them to address tensions between custodial needs and clinical requirements  *Then* equivalence in healthcare in NHS and prison service will be supported | Academic / Practitioner |
| 75 | Marks et al. (2006) p.52 (#31) | *If* GPs in prison have induction procedures such as shadowing a doctor, intensive 1 week induction, daily lectures, prison visits  *Then* GP service in prisons would be improved because GPs would not be working by trial and error in unfamiliar environment | Academic / Practitioner |
| 76 | Marks et al. (2006) p.53 (#31) | *If* GPs in prison receive ongoing peer support and a mentor  *Then* clinical, legal, and ethical dilemmas would be better identified and dealt with by GP | Academic / Practitioner |
| 77 | Marks et al. (2006) p.53 (#31);  Winter (2008) p. 180 (#157) | *If* medical records systems and filing systems were improved in prisons  *Then* the administrative system in prison would support the sort of back up found in GPs in community | Academic / Practitioner |
| 78 | Marks et al. (2006) p.53 (#31);  Winter (2008) p. 177 (#157) | *If* computers were available to prison GPs  *Then* medication prescribing, routine admin tasks, long-term condition management could be improved in prison, and fragmented care following prison transfers could be avoided, then e-health and telemedicine could be practiced | Academic / Practitioner |
| 79 | Marks et al. (2006) p.53 (#31) | *If* protocols were inter-disciplinary, if healthcare staff in prison met together to discuss common problems (as they do in the community), and if communication was better between different GPs in prison and with community GPs  *Then* continuity of care would be easier to achieve | Academic / Practitioner |
| 80 | Marks et al. (2006) p.54 (#31) | *If* GP induction included advice on prisoner behaviour, expectations of prison staff, ability of prison staff to support healthcare staff, security implications and difficulties in sending prisoners out for healthcare, and restrictions on availability of prisoners for healthcare, as well as the inevitable tensions between ‘control and care’  *Then* delivery of primary care in prisons would improve | Academic / Practitioner |
| 81 | Marks et al. (2006) p.54 (#31) | *If* GPs in prison had training on likely clinical challenges they will face, in particular in relation to MH, learning disabilities, suicide (for example by shadowing a forensic psychiatrist)  *Then* prison primary care would be more catered to prison population | Academic / Practitioner |
| 82 | Marks et al. (2006) p.55 (#31) | *If* induction for GPS in prisons included/covered:   - Prison regime and culture (including: keys, dogs, self-defence and personal security) - Rules on segregation, on shackling, and transfer of high security prisoners - Policies on fights, bullying, barricading and dirty protests - Management of disturbed patients - Prison jargon and prison specific administration - Explicit prison rules - Health care issues, including lists of services and how these can be accessed, arrangements for referral to secondary care, access to prisoners, any restrictions on prescribing and dosage requirements - Patterns of behaviour and illness behaviour in prisoners, common complaints - Drug culture and use in prisons, drugs prisoners are likely to ask for and ones they attach importance to - Clear explanation of CJS, MH Act, human rights legislation, and how these effect work of doctors in prison   *Then* primary care in prisons would be improved | Academic / Practitioner |
| 83 | Reimer (2007) p.165 (#142) | *If* the correctional healthcare provider understands inmate populations  *Then* they will be able to communicate with offenders effectively | Academic |
| 84 | Reimer (2007) p.165 (#142) | *If* the correctional healthcare provider has the ability to develop and implement a firm, consistent plan of care for the inmate  *Then* they will be able to maintain boundaries and place responsibility and accountability with the offender | Academic |
| 85 | Swenson et al. (2008) p.299, p.305/6 (#159) | *If* correctional officers work night and shift work  *Then* there are special demands on their health and performance: physiological, psychological, and behavioural changes that compromise attention, reaction time, risk taking, and efficiency, and that promote errors  *Then* organisational, home, and personal shift-adjustment procedures (such as scheduling training and education for officers during their work hours or videotaping daytime meetings; napping policies; shift-proofing the home, including posting sleep schedules, modifying the sleep environment, reducing noise disturbance, improving diet, and drinking caffeinated beverages) can help officers and their families to cope with shift stress and reduce staff turnover, sick time and absenteeism (and therefore overtime) | Academic |
| 86 | Walsh & Freshwater (2009) p.304/5/7 (#168) | *If* prison officers have appropriate mental health training (i.e. incorporating training elements outlined in table 1 p.305)  *Then* prison officers will be better able to identify prisoners at risk of developing mental health problems, identify prisoners experiencing mental health problems, and respond appropriately to the needs of these prisoners, and be able to make appropriate referrals, and will have less stigma regarding people with mental health problems, and to be less judgemental in practice with inmates with mental health problems, and more confident in dealing with prisoners with mental health problems | Academic / Practitioner |
| 87 | Walsh & Freshwater (2009) p.306/7 (#168) | *If* training for prison officers uses facilitators with on the job experience and knowledge and an ability to use prison-specific examples and prison terminology with ease, a relaxed atmosphere to encourage self-disclosure in a safe environment, the use of case studies and personal experience to support experiential learning, the use of group working, the use of didactic teaching methods and participant workbook  *Then* it will be more effective and acceptable to prison officers and will avoid resistance to change  [Context note: resistance to change in prison officers as a defence against anxiety related to change, p.307] | Academic / Practitioner |
| 88 | Walsh & Freshwater (2009) p.307 (#168) | *If* prison staff work more closely with inreach teams  *Then* this will support affirmation of their practice (relating to offenders with mental health problems) and support the development of prison officer practice in regards to offenders with mental health problems | Academic / Practitioner |
| 89 | Winter (2008) p. 170/1 (#157) | *If* systems and processes are redesigned to find, eliminate, and prevent deficiencies, and behaviour of individuals is changed, including:   - Establishment of a quality improvement leadership team - Participation of everyone in the organisation (roles and responsibilities of team members should be clearly defined and individuals held accountable for their part, and all employees trained and encouraged to identify problems and opportunities, discover causes, and develop and implement remedies) - Definition of an appropriate standard of performance - Measurement of the current process to determine problem areas - Development of a hypothesis of possible causes of identified problems - Development and implementation of strategies to improve the processes, preferably tested in a small pilot site - Modification of the quality improvement strategies based on pilot test results - Full-scale implementation of the modified quality improvement interventions - Conditioned monitoring to hold and extend gains - Ongoing communication and common understanding (including clear communication of problem areas and solutions, as well as communicating short-term progress involving visible signs of improvement to keep individuals and teams motivated   *Then* quality of healthcare in corrections can be improved | Academic |
| 90 | Winter (2008) p. 171 (#157) | *If* the internal environment of an organisation is supportive in terms of its policies, leadership, core values, and allocation of resources  *Then* a quality-conscious culture can be institutionalised | Academic |
| 91 | Winter (2008) p. 177 (#157) | *If* correctional healthcare providers had a practice-based primary care provider network to discuss similar challenges unique to the corrections environment  *Then* these would serve as a mechanism to disseminate innovations and evidence-based medical findings and knowledge transfer, link practitioners to other practitioners/academics [and provide support for GPs] | Academic |
| 92 | Winter (2008) p. 178 (#157) | If   - There is buy-in from healthcare team (which depends upon changes being seen as relevant and reasonable and the change process consistent and timely) - There are effective lines of communication vertically and horizontally - Resistance to change is overcome (achieved through education and communication, participation, facilitation and support, negotiation, manipulation, co-optation, and coercion) - A tension for change is established - There is adequate support from power groups and middle management - There is self-efficacy regarding a group’s ability to change (achieved by highlighting existing skills and previous successful change attempts) - An on-going training plan that adequately prepares change participants is provided - There is timely and accurate feedback to learn from experience - Providers accept change   *Then* change towards better quality in corrections healthcare will be supported | Academic |
| 93 | Winter (2008) p. 179 (#157) | *If* healthcare providers feel they have contributed to an improved process, rather than having it imposed on them  *Then* they are more likely to accept change | Academic |
| 94 | Winter (2008) p. 179 (#157) | *If* interventions to target different barriers to change are multi-faceted rather than single interventions, and require provider interaction rather than passive approaches  *Then* intervention is more likely to be effective in bringing about change | Academic |
| 95 | Winter (2008) p. 180 (#157) | *If* there is acknowledgement that fundamental differences in opinion about custody and treatment models may exist between security and support staff when compared to healthcare providers  *Then* understanding and cooperation between security and treatment roles will improve  *Then* the quality of care provided to inmates will improve | Academic |
| 96 | Keinan & Malach-Pines (2007) p. 395 (#319) | *If* training programmes are provided for prison personnel to reduce stress, including simulations of typical stress situations, analysing stressful events from the past, practicing ways to cope with such situations  *If* the perception that one’s job is meaningful and important is enhanced (e.g. through talks from senior staff emphasising importance of work, or analysis of successful events like workshops in which prison personnel actions that were successful in preventing violence are presented and analysed)  *If* workshops for prison employees and their families are held to support work-life balancing difficulties  *If* there is a program of physical training for all prison employees, and a fitness room in each prison for staff  *If* a stress-management unit is opened in each prison authority including stress management and other related professionals focusing on individual and organisation levels  *Then* the high levels of burnout and stress experienced by prison personnel in their work will be reduced | Academic / Practitioner |
| 97 | Griffin et al. (2012) p. 1140 (#473) | *If* prison employees have job autonomy and job variety  *Then* they will experience less burnout and more positive employee psychological states | Academic / Practitioner |
| 98 | Griffin et al. (2010) p. 252 (#474) | *If* prison staff experience burnout  *Then* this is harmful to the inmates and the organisation | Academic |
| 99 | Robillard et al. (2011) p.348 (#177) | *If* staff working with inmates and releasees are honest (for example about the types of service available and not promising services that are not available)  *Then* it is more likely they will establish a positive relationship with the client | Academic / Practitioner |
| 100 | Robillard et al. (2011) p.352 (#177) | *If* there is a lack of services on release  *Then* clients sometimes choose reincarceration | Practitioner |
| 101 | Robillard et al. (2011) p.352 (#177) | *Many frontline staff agreed that obtaining services for their clients was the most time-consuming and challenging aspect of their job. For clients with multiple service requirements there were more interactions required by staff on clients behalf* | Practitioner |
| 102 | Robillard et al. (2011) p.352 (#177) | *If* there are more pressing basic needs on release, such as housing or food  *Then* medical care is not a priority for releasees | Practitioner |
| 103 | Robillard et al. (2011) p.352 (#177) | *Many prisoners were less willing to remain in services on release. In prison the service had filled their day, but in the community there were so many other factors affecting their lives.* | Practitioner |
| 104 | Robillard et al. (2011) p.352 (#177) | *If* staff work hard to get releasees in to services and clients do not work to stay in the program (or if they leave program and return to staff to get service access at later date  *Then* staff can experience frustration | Practitioner |
| 105 | Robillard et al. (2011) p.353 (#177) | *If* staff understood and respected their clients decisions, maintained honesty, and appreciated teeny tiny steps  *Then* client success was more likely | Practitioner |
| 106 | Robillard (2011) p.352 (#177);  Peer Researcher Group Meeting, October 2013 | *If* former inmates serve as employees with services, such as housing  *Then* this can be very effective with offender clients and also positive for the individual | Practitioner |
| 107 | Robillard et al. (2011) p.353 (#177) | *If* there are a multitude of services working within a facility  *Then* good communication between the different entities, including staff, affects how effectively a program operates | Practitioner |
| 108 | Robillard et al. (2011) p.353 (#177) | *If* health care providers providing in-reach services in prisons have strong relationships with correctional officers (who are seen as gatekeepers “in charge” who have decision making authority regardless of administrative mandates)  *Then* in-reach healthcare services are less successful (in accessing clients needing service) | Practitioner |
| 109 | Robillard et al. (2011) p.353 (#177) | *If* releasees do not have documentation on release to access services  *Then* they can’t access services  *If* prison issues all releasees a birth certificate on release  *Then* this removes a barrier to service access post-release | Practitioner |
| 110 | Robillard et al. (2011) p.356 (#177) | *If* a social ecological perspective to intervention development is taken in which the practitioner (who is central in transitioning the clients from corrections to community) is central rather than the client  *Then* intervention will support the practitioner to contact and obtain the relevant services and support for clients (which multiple aspects of intervention at multiple levels need to be in place) | Academic / Practitioner |
| 111 | R. E. Martin et al. (2009) p.99 (#55) | *If* women in Prison are engaged in participatory health research (this kind of research involves the subjects of the research in various ways) which involves discussions on spiritual and emotional healing  *Then* some women within those groups will begin to adopt peer support roles. | Academic |
| 112 | Barber et al. (2006) p. 4/5 (#2) | *If* offenders have a style of attachment that is anxious/avoidant or anxious/ambivalent  *Then* it will be difficult to develop a positive and supportive therapeutic relationship with them | Academic / Practitioner |
| 113 | Barber et al. (2006) p. 6 (#2) | *If* mental healthcare staff delivering therapeutic interventions have not established a secure base for themselves (feeling validated and respected by one another and consistently contained and supported by the system)  *Then* they are unable to provide a secure base for patients | Academic / Practitioner |
| 114 | Barber et al. (2006) p. 6 (#2) | *If* there is shared ownership of the secure attachment model of care, with the core team having in-depth knowledge of the patient, working collaboratively together to support each member in maintaining consistent availability and a consistent and empathic response, and boundary maintenance  *Then* the therapeutic team will have greater tolerance of the toxicity of the offender’s interpersonal dynamics | Academic / Practitioner |
| 115 | Barber et al. (2006) p. 8 (#2) | *If* an offender has a more disrupted and incoherent attachment pattern before attachment therapy  *Then* they may be more at risk of reverting to previous more maladaptive attachment strategies at times of transition, when they experience actual or threatened separations from caregivers | Academic / Practitioner |
| 116 | Curd et al. (2007) p.305 (#148) | *If* interventions in correctional settings are participatory, with involvement and support from the upper levels of the corrections facility down to e.g. substance abuse programme residents; are community based; socially valid to the realities of the community  *Then* it will be more likely to be successful and can tap in to the community’s energy, enthusiasm, and willingness to participate in a program where there was a sense of ownership | Academic |
| 117 | Curd et al. (2007) p.306 (#148) | *If* there is a robust organisational ability to effectively use available resources to promote inmate health and if an intervention does not introduce a need for new resources but rather a focused redirection of resources already being used in the setting  *Then* the intervention will have on-going success | Academic |
| 118 | Sumter et al. (2009) p. 47/54 (#161) | *If* women in prison are taught meditation practice   - *Then* they experience fewer sleeping difficulties, less desire to throw things or hit people, and less nail or cuticle biting; and are more hopeful about their future; and feel less guilt - *Then* they experience a reduction in medical symptoms, negative emotions and behaviours | Academic |
| 119 | Sumter et al. (2009) p.49 (#161) | *If* people in prison are taught meditation techniques  *Then*   - they may find meditation a useful tool in coping with stresses of incarceration - substance abuse may decline - violence may be reduced | Academic |
| 120 | Sumter et al. (2009) p.49 (#161) | *If* meditation is used in prisons  *Then* it could be a cost-effective tool for helping with inmate problems such as insomnia, substance abuse, stress, and conflict management | Academic |
| 121 | Sumter et al. (2009) p.54 (#161) | *If* meditation is practiced in prison by female inmates  *Then* it helps people to manage their feelings of frustration and anger, gives them more emotional stability and control over their lives, gives them positive techniques to deal with their frustration and anger in addition to releasing negative emotions, which reduces the likelihood of them wanting to throw things or hit people | Academic |
| 122 | Sumter et al. (2009) p.54 (#161) | *If* meditation is practiced in prison by female inmates  *Then* they are less likely to bite their nails and cuticles suggesting that it reduces stress and anxiety and supports them to remain focused, peaceful and calm in any situation | Academic |
| 123 | Sumter et al. (2009) p.55 (#161) | *If* meditation is practiced in prison by female inmates  *Then* they are more hopeful about the future and feel less worry and guilt | Academic |
| 124 | Marle (2007) p.117 (#32) | *If* staff ’support group cohesion, faciltate education and recreation and have respect for the man-behind-the-offender’  *then* ward atmosphere will be improved | Academic |
| 125 | Marle (2007) p.117 (#32) | *If* there are ’circles of stress’ in the relationship between staff and prisoners  *then* ’the well-being of both parties is compromised’ | Academic |
| 126 | Marle (2007) p.118 (#32) | *If* mental health care promotion and education is delivered to *all* prisoners  *then* equality of mental health care provision between prison and society will be achieved | Academic |
| 127 | Marle (2007) p.120 (#32) | *If* a secure environment in prison is provided – ’[where secure is defined as] predictable, with transparent regulations and an empathic attitude based on interaction between inmates and between inmates and staff’  *then* continuity of care will be attained | Academic |
| 128 | Marle (2007) p.120 (#32) | Offenders’ motivation can be highly important for preventing and/or addressing mental health issues.  The prison environment and interactions within it have a significant impact on offenders’ motivation, e.g.:  - an atmosphere of tolerance  - explanation of rules and interventions  - offering choice of interventions and providing feedback and education about current interactions  - offenders’ trust in services, e.g. ’that their treatment will last as long as necessary and that they will not be let down when released’ | Academic |
| 129 | C. Brooker et al. (2009) p.173 (#58) | *If* integrated mental health services (including dual diagnosis, common mental health disorders, and personality disorder) are provided in prisons  *then* the mental health of offenders will be improved | Academic |
| 130 | C. Brooker et al. (2009) p.173 (#58) | *If ’*offenders leaving prison with mental health issues are stigmatised by routine mental health service providers’  *then* offenders are frequently excluded from mainstream community mental health services | Academic |
| 131 | C. Brooker et al. (2009) p.174 (#58) | *If* commissioners of offender health care were ’held to account for the management of offenders transition to mainstream mental health services on release’  *then* continuity of care for offenders with mental health problems will be improved | Academic |
| 132 | Backhouse (2007) p.79 (#21) | *If* there are prison-induced factors that hinder healthcare delivery in prison, such as overcrowding, prison procedures, culture and practices  *Then* healthcare services will be less likely to be equivalent to patients living in the community | Academic |
| 133 | Backhouse (2007) p.79 (#21) | *If* there are patient-induced factors that hinder healthcare delivery in prison, such as clinician fear of violence, restricted choice of clinician for patient  *Then* healthcare services will be less likely to be equivalent to patients living in the community | Academic |
| 134 | Backhouse (2007) p.79 (#21) | *If* there are NHS-induced factors that hinder healthcare delivery in prison, such as low priority because outside the scope of normal NHS target setting or under-funding  *Then* healthcare services will be less likely to be equivalent to patients living in the community | Academic |
| 135 | Backhouse (2007) p.80 (#21) | *If* prison healthcare is nurse led as opposed to the traditional referral to GP (i.e. nurse-led triaging on the ward supported by minor intervention and nurse-led practice and screening based in healthcare centre in prison)  *Then* prisoners are enabled to promptly access skilled practitioners offering first level nurse triage and intervention  *Then* nursing staff will have greater morale through using their skills effectively  *Then* GP appointments can be limited to those appropriate and relevant to patient need | Academic |
| 136 | Ricketts et al. (2007) p.240 (#34) | *If* an in-reach mental health team has the ability to adapt to the context of the prison  *Then* they will have a greater impact in the prison | Practitioner |
| 137 | Ricketts et al. (2007) p.240 (#34) | *If* clinical decisions can be or are overruled by a Governor  *Then* inreach staff will be less effective and feel disempowered | Practitioner |
| 138 | Ricketts et al. (2007) p.240 (#34) | *If* inreach mental health team members do not have access to keys (and for example cannot get out of their office to go to the toilet)  *Then* they will feel disempowered and more aware of the culture clash between the prison and NHS | Practitioner |
| 139 | Ricketts et al. (2007) p.241 (#34) | *If* there is a strong us versus them culture between the prison and NHS staff in in-reach teams  *Then* mental health inreach services are less able to offer service equivalence to community MHTs  *But* culture clash can also provide a positive asset, for example providing an outsider perspective, making things less polarised and potentially less abusive | Practitioner |
| 140 | Ricketts et al. (2007) p.241 (#34) | *If* inreach teams can liaise with the Care Programme Approach co-ordinator CPN who is full-time in the prison  *Then* they can start the referral process as soon as need is identified and start making contacts (with other services) | Practitioner |
| 141 | Ricketts et al. (2007) p.242 (#34) | *If* inreach MHTs work with patients who are awaiting transfer out of prison into hospital facilities (which in many inreach teams is seen as outside of their remit and rather the responsibility of the other parts of the prison healthcare service  *Then* continuity of care would be improved | Practitioner |
| 142 | Ricketts et al. (2007) p.242 (#34) | *If* training prison staff in mental health awareness is seen as part of the remit of the MH inreach teams  *Then* stigma of prison officers, prisoners would be reduced, and prison staff confidence in dealing with MH issues as well as appropriate referral of MH problems in prison would be improved | Practitioner |
| 143 | Ricketts et al. (2007) p.242 (#34) | *If* MH awareness training for prison officers by inreach teams is a three day course  *Then* it is too long and will not be allowed or attended in prison | Practitioner |
| 144 | Ricketts et al. (2007) p.242 (#34) | *If* there are good relationships between the inreach team and relevant other teams within prison  *Then* referrals are more likely to come through and the service valued by prison staff | Practitioner |
| 145 | Ricketts et al. (2007) p.242 (#34) | *If* prison officers feel they have a good relationship with inreach team member  *Then* they are more likely to work with them | Practitioner |
| 146 | Ricketts et al. (2007) p.242 (#34) | *If* prison officers can just pick up the phone and talk about a MH related issue in prison or with prisoner, and it doesn’t involve paperwork  *Then* it is easier for them and they like it | Practitioner |
| 147 | Ricketts et al. (2007) p.242/3 (#34) | *If* inreach MH teams have good relationships outside the prison with an individual in a relevant service  *Then* they can work more effectively, for example getting prisoners transferred to other services outside prison or in another prison | Practitioner |
| 148 | Ricketts et al. (2007) p.243 (#34) | *If* the inreach team manager and the healthcare manager in the prison have a good relationship  *Then* the inreach team can work more effectively and overcome frustrations and difficulties in working within the prison system more easily | Practitioner |
| 149 | Ricketts et al. (2007) p.243 (#34) | *If* an inreach team has built up networks within and outside of the prison  *Then* they can work more effectively | Practitioner |
| 150 | Ricketts et al. (2007) p.243 (#34) | *If* the MH inreach team has a strong leader, who can rise to the challenge of providing leadership both within the team and in the wider prison and health system context  *Then* the inreach team will be more effective in overcoming contextual problems of working in prisons and providing equivalent to community healthcare to prisoners | Practitioner |
| 151 | Ricketts et al. (2007) p.245 (#34) | *If* MH inreach teams link staff on the landings, primary healthcare in prison, probation, and the mainstream NHS services  *Then* they will be more successful | Practitioner |
| 152 | Duncan et al. (2006) p. 217 (#51) | *If* problem solving and anger management structured group interventions are carried out in secure hospital settings with mentally disordered offenders  *Then* they will have a moderate to high effect on mentally disordered offenders’(social) problem solving and anger management | Academic |
| 153 | McMurran (2007) p. 228 (#78) | *If* post-sentence testing is carried out (for drug using offenders)  *Then* it opens the possibility for treatment in prison and follow-through on licence | Academic |
| 154 | McMurran (2007) p. 228 (#78) | *If* arrest-referral schemes are carried out (for drug using offenders) with drug workers employed to approach arrestees in custody and offer advice or channel them into treatment  *Then* early identification and referral are possible and treatment throughout prison and into community are supported  [Mark, I put this in as although review is drug user specific it potentially could inform mental health services points for MH issue identification and referral to treatment? Is review of what works in drug misuse treatments for offenders] | Academic |
| 155 | McMurran (2007) p. 229 (#78) | *If* there is effective multi-disciplinary and inter-agency working  *Then* diversion from criminal justice into treatment is supported | Academic |
| 156 | McMurran (2007) p. 229 (#78) | *If* offender programmes are more responsive by selecting offenders for treatment relevant to their needs (in terms of type of programme on offer, intensity of treatment, and specific needs of sub-groups of offenders)  *Then* there will be less attrition from these programmes (a big problem with offender interventions) | Academic |
| 156 | McMurran (2007) p. 229 (#78) | *If* offender programmes are more responsive by selecting offenders for treatment relevant to their needs (in terms of type of programme on offer, intensity of treatment, and specific needs of sub-groups of offenders)  *Then* there will be less attrition from these programmes (a big problem with offender interventions) | Academic |
| 157 | McMurran (2007) p. 229 (#78) | *If* offender interventions can reduce their attrition rates  *Then* a greater number of offenders can be supported to better health | Academic |
| 158 | McMurran (2007) p. 230 (#78) | *If* interventions are higher dosage, with at least 26 weeks’ duration, two or more contacts per week, and more than 100 hours of treatment  *Then* these will be most effective for high risk offenders and serious, long-term drug users  [and potentially for effectively treating MH issues in some sub groups of offenders?] | Academic |
| 159 | McMurran (2007) p. 230 (#78) | *If* there is a stepped care approach in prison treatment programmes (as in MH NHS services)  *Then* care will suit the needs of different groups of offenders; for example, with brief interventions working for some groups of offenders, and more intensive ‘high dosage’ treatment programmes working for those unaffected by brief interventions. This is a more responsive model of care for offenders. | Academic |
| 160 | McMurran (2007) p. 231 (#78) | *If* services are designed to be responsive to specific offender groups  *Then* programmes for offenders will be more effective and have less attrition | Academic |
| 161 | Unruh et al. (2009) p.290) (#105) | *If* a ‘transition specialist’ provides interactions that stress self-determination, social skill training, and developing a set of services based on the needs and strengths of the youth offender and generally supports youth engagement in services  *Then* these interactions may be more powerful than offender being only engaged in education and employment and will support youth offender engagement in services | Academic |
| 162 | Unruh et al. (2009) p.291 (#105) | *If* youth offender has a mentoring type relationship  *Then* their transition from facility to community is improved | Academic |
| 163 | Unruh et al. (2009) p.291 (#105) | *If* there is a targeted set of community-based services that focus on the unique needs of each individual and their families or support network  *Then* service engagement in community of youth offenders will be higher | Academic |
| 164 | Unruh et al. (2009) p.291 (#105) | *If* an individual, such as a transition specialist, can provide the guidance to ensure the unique service needs of each youth, family, and support network and providing a positive and mentoring relationship during the critical community adjustment phase  *Then* this supports the unique needs of individuals across multiple settings and agencies in the community | Academic |
| 165 | Unruh et al. (2009) p.291 (#105) | *If* there are additional services around a particular service (housing, education, accommodation, MH etc)  *Then* these services may provide the stabilisation needed for the individual to engage with that particular service | Academic |
| 166 | Unruh et al. (2009) p.292 (#105) | *If* there are targeted and appropriate wraparound services in the community for youth with mental health needs  *Then* this may prevent them from escalating to (re) involvement with CJS | Academic |
| 167 | Murray et al. (2013) p. 4 (#110) | *If* a committee of individuals from high levels of all relevant organisations/services is created with the authority to provide direction and instigate modifications as deemed necessary  *Then* the infrastructure to guide organisational/system change is supported through clarity and consistency of efforts and roles | Academic |
| 168 | Murray et al. (2013) p. 2/3 (#110) | *If* organisation wanting to undergo change starts with the assumption that all staff have the skill set that enables them to incorporate the tools from training into practice with modest levels of support and consultation  *Then* improvement theory may not be turned in to practice in organisation at practitioner level | Academic |
| 169 | Murray et al. (2013) p. 5 (#110) | *If* there is an understanding of the why behind the intervention or change in practice  *Then* implementation of practice improvement is supported | Academic |
| 170 | Murray et al. (2013) p. 5 (#110) | If there is a needs assessment of staff members and their current training needs and how training can be provided  *Then* implementation of practice improvement is supported | Academic |
| 171 | Murray et al. (2013) p. 5 (#110) | *If* there is staff training including a 2 day training event to show importance of new practice and follow-up refresher training sessions for sustaining training and training for new staff (through training staff to train other staff)  *Then* implementation of practice improvement is supported | Academic |
| 172 | Murray et al. (2013) p. 7 (#110) | *If* staff across different organisations and services are given the same training  *Then* it gives everyone a common language (which can support inter-team communication and understanding) | Academic |
| 173 | Murray et al. (2013) p. 7 (#110) | *If* tools used to implement service improvement or organisational change are able to be revised in local settings  *Then* there is more ownership and tools can better fit the model of care or culture of local services or organisations | Academic |
| 174 | Murray et al. (2013) p. 8 (#110) | *If* there is strong leadership and buy in from staff at all levels of the organisation  *Then* there is greater ownership and understanding through the organisation that facilitates implementation | Academic |
| 175 | Murray et al. (2013) p. 8 (#110) | *If* researchers/developers have a good understanding of the organisations starting point, current practices, management structure/approach, and philosophy  [*Then* intervention will be context-appropriate. Note: this links to complexity-led intervention development] | Academic |
| 176 | Fortune et al. (2010) p. 190 (#129) | *If* a PD offender service has a long assessment period including interviews with various members of a multidisciplinary team  *Then* this is a barrier to engagement of service users in further treatment | Academic |
| 177 | Fortune et al. (2010) p. 190 (#129) | *If* staff in a PD offender service are young and still in training  *Then* PD ofenders will be able to manipulate them and perceive them as naïve and vulnerable | Academic |
| 178 | Fortune et al. (2010) p. 190/1 (#129) | *If* staff in a PD offender service are honest with themselves, self-aware, imaginative, motivated and able to hold personal boundaries  *Then* PD offenders are more engaged in treatment and can build relationships with PD service providers | Academic |
| 179 | Fortune et al. (2010) p. 192 (#129) | *If* prisoners, prison transfers, sex offenders and mental health patients are mixed together in treatment  *Then* it can cause tensions in treatment between different offender profiles and groups | Offender |
| 180 | Fortune et al. (2010) p. 190 (#129) | *If* there is a successful relationship between staff and PD offender client characterised by a perception of mutual trust and respect with staff seen as honest, tolerant, and non-judgemental  *Then* PD offender treatment will be experienced by offender and staff as more helpful | Offender, Practitioner |
| 181 | Fortune et al. (2010) p. 190 (#129) | If relationships between multi-disciplinary teams, such as staff in an inpatient unit, are strained and there are power struggles between discipline leaders ‘fighting’ for overall leadership of group  Then staff will be unclear about who holds ultimate clinical responsibility | Practitioner |
| 182 | Fortune et al. (2010) p. 194 (#129) | *If* work with offender’s with PD has the underlying ethos of providing some form of constancy and avoids frequent changes of professionals (i.e. limited use of bank or inexperienced staff and rather the use of a skilled pool of staff who are familiar with both the service and client group)  *Then* feelings of loss and abandonment that characterised offenders previous relationships will not be reawakened | Academic |
| 183 | Goldstein et al. (2006) p. 189/195/196 (#136) | *If* a mental health clinician (MHC) model of care [a stepped care approach] is used in a US jail where inmates requesting psychiatric care are screened for possible referral to a MHC clinic, if they meet certain criteria they receive initial evaluation by a MHC rather than direct referral to psychiatrist. Inmate is then referred to either holistic treatment to address problems such as sleep disturbance, anxiety, depression, and drug withdrawal or to a psychiatrist  *Then* many inmates are treated successfully by holistic treatment (low-level), referred to appropriate other jail services, or treated by a psychiatrist and psychiatric waiting times in jail are significantly reduced | Academic |
| 186 | Richard Byng, Personal Communication, October 2013 | *If* ideographic goals in different outcome domains (e.g. mental health or housing) are linked  *Then* two-way causation between domains will be enhanced  *Then* an outcome in one domain can support an outcome in another domain | Study Group |
| 187 | Richard Byng, Personal Communication, October 2013 | *If* practitioners utilise their strengths and creativity  *Then* intervention effectiveness and resultant wellbeing will be improved | Study Group |
| 188 | Study Group Meeting, November 2013 | *If* offenders can form or re-connect with a supportive social environment which offers opportunities for pursuing a non-offender identity *then* resettlement and rehabilitation is promoted | Academic |
| 189 | Study Group Meeting, November 2013 | *If* offenders have the opportunity to explore with a practitioner their personal narrative in a non-judgmental environment  *and* support is provided for building and/or re-modelling an identity that is coherent with but does not discount past identities  *then* resettlement and rehabilitation is promoted. | Academic |
| 190 | Study Group Meeting, November 2013 | *If* practical and emotional support are offered in isolation from each other  *then* offenders’ progress towards wellbeing is not fully promoted | Academic |
| 191 | Study Group Meeting, November 2013 | *If* services are commissioned around conventional organisational structures rather than the people who those organisations are designed to serve  *then* the likelihood of collaborative, person-centred service provision is lessened | Academic |
| 192 | Study Group Meeting, November 2013 | *If* practitioners can develop the skills to reflect on their own practice  *and* work towards practising empathically  *then* offenders’ well-being will increase | Academic |
| 193 | Study Group Meeting, November 2013 | *If* practitioners can ‘repair’ relationships with offenders when offenders feel that this relationship has ‘gone wrong’  *then* progress towards resettlement and rehabilitation can be maintained | Academic |
| 194 | Study Group Meeting, November 2013 | *If* the positive (but ultimately short-term) practitioner relationship provides support for the repair or creation of an/other primary relationships in an offender’s life  *then* offender’s social support in community will be improved  *and then* progress towards resettlement and rehabilitation can be maintained beyond practitioner involvement | Academic |
| 195 | Study Group Meeting, November 2013 | *If* offenders have previous positive or negative experiences of services  *then* these experiences will impact strongly on their current level of engagement with services | Academic |
| 196 | Study Group Meeting, November 2013 | *If* offenders can be engaged by appropriate services at the start of their time in prison  *then* a positive precedent for continuing engagement with services is set | Academic |
| 197 | Study Group Meeting, November 2013 | *If* offenders’ concerns about past experiences  *and* their expectations about service provision are discussed  *then* engagement can be started and maintained even where service provision is imperfect | Academic |
| 198 | Study Group Meeting, November 2013 | *When* offenders see health services in prison as one service but practitioners see them as separate services with separate provision  *then* it is more likely there will be repeated assessment or disjointed care provision and  *then* offenders will be more likely to become disengaged with all health services in prison | Academic |
| 199 | Study Group Meeting, November 2013 | *If* all contacts between an offender and any practitioners in prison and community are conceived of as potentially ‘therapeutic’ by the practitioners  *then* offender engagement with services is supported | Academic |
| 200 | Study Group Meeting, November 2013 | *If* the very first contact between services and offenders emphasises the positives without flagging up any negatives of service engagement  *and* if first contact gives the offender something they need/want  *then* offenders’ first perception of services is positive  *then* this will affect the motivation of the offender to engage with services | Academic |
| 201 | Study Group Meeting, November 2013 | *If* there is good assessment practice, such that offenders do not have repeated assessments from different services  *then* engagement with services will increase | Academic |
| 202 | Study Group Meeting, November 2013 | *If* practitioner acts as an advocate for the offender, practicing assertive outreach and getting the offender it to services they need  *then* offender service engagement will increase | Academic |
| 203 | Study Group Meeting, November 2013 | *If* offenders’ and practitioners’ ability to look at another person’s perspective is increased through the use of mentalisation-based skills training  *then* practitioner creativity will be supported  *and* offender relationships will be supported | Academic |
| 204 | Chew-Graham et al. (2008) p. 6 (#12) | *If* there is a lack of direct doctor to doctor communication  *Then* this will contribute to fragmentation of patient care | Academic |
| 205 | Chew-Graham et al. (2008) p. 6 (#12) | *If* a primary care practitioner has reached their personal threshold for dealing with a case  *Then* they will refer to secondary services | Academic |
| 206 | Goodwin (2008) p.59 (#14) | *If* all collaborators within a network feel the net worth of their involvement in the network (i.e. practitioners, institutions, agencies) through appropriate incentives  *Then* collaborative working is enabled | Academic |
| 207 | Calnan & Rowe (2008) p. 97 (#15) | *If* there is trust between services in terms of the care quality, empathy, and competence of practitioners in that service  *Then* inter-agency working is supported | Academic |
| 208 | Fraser (2009) p. 133 (#56) | *If* the public had a better awareness and understanding of the prisons’ role in society, including prisons’ commitment to mental health and illness  *Then* this would support prisoner MH post-release (i.e. through better reintegration into communities etc.) | Academic |
| 209 | Scott & Doughty (2012) p. 154 (#91) | *If* notes about a patient are made in a collaborative, empowering and transparent manner  *Then* patients are empowered, trust is engendered between patient and provider, and person-centred care is supported | Academic |
| 210 | Tirril Harris, Individual Communication, November 2013 | *If* emotional support/befriending is provided by a volunteer (rather than only by a professional) who continues to be involved with the offender’s life on release  *then* the benefits of emotional support extending over months rather than weeks can be realised  *and* emotional support will be experienced as genuine by offenders | Academic |
| 211 | Tirril Harris, Individual Communication, November 2013 | *If* an offender has one person in their life who offers good emotional support  *then* wellbeing is increased and recidivism decreased | Academic |
| 212 | Tirril Harris, Individual Communication, November 2013 | A map of *local* resources available to offenders can provide a valuable guide for both offenders and practitioners (who can signpost these services) | Academic |
| 213 | DeHart et al. (2009) p.129 (#162);  Short et al. (2009) p.408 (#206) | *If* practitioners perceive inmate self-injury to be a coping mechanism and ‘genuine’, rather than an attempt to manipulate and ‘non-genuine’  *And* If they feel more confident and trained and supported in their welfare role (in addition to their custody role)  *Then* they are more likely to respond compassionately, feel confident to deal with self-harming offenders, and to respond with a referral to therapy rather than to isolation | Academic |
| 214 | Nedderman et al. (2010) p. 171 (#171) | *If* an intervention instils hope  *Then* this hope will act as a therapeutic factor and motivating energy that promotes goal development, reduction of negative emotions, and coping ability. | Academic |
| 215 | Wallace et al. (2011) p.336 (#176) | *If* practitioners and consumers can select from a recommended menu of evidence-based and promising treatments capable of addressing the comorbidity of interpersonal trauma, mental illness, and substance misuse to create individually tailored, integrated treatments  *Then* it is possible to respond to an offender’s unique constellation of issues | Academic |
| 216 | Wallace et al. (2011) p.340 (#176);  Charlie Brooker & Birmingham (2009) p. S3 (#212) | *If* there is trauma screening and assessment to identify those most vulnerable to trauma in the prison setting as well as practitioner training and effective monitoring of offenders to prevent and respond to incidents of trauma (because there is a high risk or trauma in prison in addition to a likely prior history of trauma in an offenders past)  *And* practitioners are skilled in dealing with people who have experienced abuse/trauma in the past  *Then* risk of re-traumatisation in prison and the community is reduced  *And* offender wellbeing is improved | Academic |
| 217 | Marlow et al. (2012) p. 8 (#178) | *If* care interventions with offenders address problematic coping and communication styles (e.g. Nonviolent Communication Training)  *Then* their empathy will increase  *Then* this will support them to build and sustain positive social support networks | Academic |
| 218 | Chafin & Biddle (2013) p. 124 (#185) | *If* retention of practitioners working in correctional settings is supported through services such as new hire orientation, clinical ladder programs, and team building  *Then* offender healthcare will be improved | Academic |
| 219 | Hayward et al. (2008) p. 243 (#202) | *If* an intervention targets social problem solving skills  *Then* this supports offenders to cope with being imprisoned and to be less vulnerable | Academic |
| 220 | Khalifa et al. (2008) p.2/3 (#200) | *If* telepsychiatry is used as a delivery method for mental health interventions in prisons  *Then* it increases access to mental healthcare services for offenders, reduces expense and resource challenges of service delivery in prison, and improve the quality and equivalency of care provided in prisons | Academic |
| 221 | Hall & Long (2009) p.481 (#207) | *If* interventions use progressive muscle relaxation techniques  *Then* this will act as a both a prophylactic, to reduce hypothesised high level of arousal or tendency to become easily agitated or anxious, and a therapy, helping develop a portable coping strategy to manage anxiety/high arousal ‘in situ’ on a daily basis | Academic |
| 222 | Ferguson et al. (2009) p.906 (#210) | *If* goal setting and planning skills in offenders are enhanced through intervention  *Then* wellbeing will be enhanced and symptoms of mental illness reduced | Academic |
| 223 | Wolff & Draine (2004) p.461 (#310) | *If* imprisonment weakens offenders’ connections with the community  *Then* offenders identify more with prison culture  *Then* individuals’ confidence in, and ability to, live independently (non-institutionally) is significantly weakened | Academic |
| 224 | Wolff & Draine (2004) p.466 (#310) | Psychopathology and behavioural history can negatively affect the ability of offenders to ‘mobilise the goodwill and resources embedded in their social relations’ | Academic |
| 225 | Wolff & Draine (2004) p.471 (#310) | *If* practitioners assume that offenders’ families can offer support (social, emotional, values, resources) in a similar way to ‘conventional middle-class families’  *Then* the potential for enabling offenders to mobilise their social capital is lessened | Academic |
| 226 | Mezey et al. (2010) p.693 (#225) | *If* the recovery approach to recovering from mental illness is adapted to forensic populations such that it provides offenders with realistic expectations about what lies ahead, rather than false hope; and takes into account the importance of self-esteem and stigma in this population  *And* takes in to account the offender and their family/communities perceptions of their offending behaviour as a barrier to recovery  *Then* this approach can better support recovery from mental illness in forensic populations | Academic |
| 227 | Mezey et al. (2010) p.692 (#225) | Some of the central concepts around recovery in the recovery literature, i.e. hope, self-acceptance, self-management, and having ones achievements recognised may be particularly problematic for forensic psychiatric patients. | Academic |
| 228 | Minoudis et al. (2012) p.230 (#230) | *If* prison officers have the confidence to challenge erroneous rejections of referrals from MH teams  *Then* referral success will be increased | Academic |
| 229 | Lambert et al. (2010) p.1230 (#479);  Lambert et al. (2012) (#481) | *If* prison officers feel supported by, and trust, colleagues and supervisors  *then* social and practical work issues are more likely to be addressed  *then* job burnout is decreased | Academic |
| 230 | Lambert et al. (2011), p.455 (#480) | *If* there is congruence between staff’s and the organisation’s (prison’s) values  *then* staff satisfaction in the workplace is improved  *and* staff implement care/custody programmes in the way intended by the organisation | Academic |
| 231 | Devilly et al. (2005) p.229 (#259) | *If* peer counselling is used instead of professional counselling  *Then* it can take up more of the counsellors time than counselling themselves and there is some evidence suggesting offenders prefer counselling by professionals | Academic |
| 232 | Dhaliwal & Harrower (2009) p. 35 (#274);  Jaffe (2012) #316 | *If* offenders act as listeners in prison  *Then* they experience significant personal growth and changing attitudes to self and others. | Academic |
| 233 | Van Harreveld et al. (2007) p. 697 (#320) | *If* offenders use an active emotion-focused coping strategy by sharing negative emotions with people in their social network  *And* engage in emotion management in a more cognitive way, by emphasising more positive aspects of the situation  *Then* they have better mental and physical health | Academic |
| 234 | Brough & Williams (2007) p. 566 (#321) | *If* prison officers have supervisory support  *Then* it will reduce the impact of job demands on adverse job satisfaction for correctional officers | Academic |
| 235 | Martinez (2010) p.145-7 (#346) | *If* offenders’ relationships (and the responsibilities that these relationships entail) can be resumed or initiated at the time of release  *Then* a positive upward spiral of social integration and mutual obligation is enabled | Academic |
| 236 | Opie (2012) p.210-11 (#348) | *If* offenders can be helped to articulate their own vision of the future (‘one where one’s relationship to the social is not primarily determined by one’s criminal record’)  *and* ongoing help to enable further reflection and elaboration is provided  *Then* ‘self-respect and a desire to contribute socially [are] nurtured’  *and* the potential for wellbeing of the person in society is improved | Academic |
| 237 | Persons (2009) p.439 (#448) | *If* offenders with mental health issues (depression, PTSD, conduct disorder) are engaged using art therapy  *Then* they have a positive avenue for self-expression across a range of psychological needs (identity, security, adventure/fun, parental relationships, affiliation and affection, erotic and sexual needs, experience of depression, expression of childhood trauma and other serious psychological problems, and spiritual or religious needs)  *and* destructive behaviour is reduced  *and* mental wellbeing is improved | Academic |
| 238 | Nee & Farman (2007) p.176 (#196) | *If* DBT, which offers a large range of skills which can be effectively tailored to meet the needs of individual prisoners is used with offenders in prison  *Then* the constraints of the prison environment can be radically accepted which supports offenders to cope with prison | Practitioner |
| 239 | Nee & Farman (2007) p.176 (#196) | *If* an approach is used with offenders with a PD that focuses on developing core mindfulness skills, with an emphasis on awareness of the moment and identification of mood states  *Then* this will reduce dissociation and reckless behaviours  Then offenders will be more capable of learning other types of skills because of this which can also increase sense of self and regulation of emotion and thus quality of life | Practitioner |
| 240 | Gudjonsson & Young (2007) p. 548 (#199) | *Present a model of organisational plan of psychological therapies for forensic inpatients. Incorporates many aspects we are interested in in care planning for offenders with CMHPs. Not sure how best to use this but feel we should.* | Practitioner |
| 241 | Crawford & Rutter (2007) p. 60 (#332) | *If* efforts are made to change the environment of prisoners  *Then* offenders with PD who are unwilling to engage in therapy may still be helped to achieve better mental health | Academic |
| 242 | Crawford & Rutter (2007) p. 58 (#332) | *If* limits on availability of staff and other boundaries are made clear to offenders with PD when engaged with treatment services  *And* there are facilities to access more intensive services at times of crisis, inc telephone support or immediate access to some type of support  *Then* offenders with PD will be more effectively supported by services | Academic |
| 243 | McMurran & Ward (2010) p. 82 (#150) | *If* individuals display lower levels of distress  *Then* they may benefit from more prescriptive, group-based therapies  *But* if they have higher levels of distress  *Then* they may be better suited to individually tailored and implemented treatment | Academic |
| 244 | McMurran & Ward (2010) p. 82 (#150) | *If* offenders are encouraged to detect the needs and personal goals associated with their past offending and clinical inquires abuout other possible ways of achieving these  *Then* their implicit life plan will be made more explicit and it will be easier to focus on the things that matter most to them and to think about what social and psychological resources are needed to implement such plans in the future | Academic |
| 245 | McMurran & Ward (2010) p. 83 (#150) | MI is a brief treatment approach that can be used to increase problem recognition and treatment take-up. It is client-centred, directive method for enhancing intrinsic motivation to change by exploring and resolving ambivalence | Academic |
| 246 | Ungar et al. (2012) p. 14 (#521) | *If* vulnerable people exercise agency and are involved in the negotiations for service provision and discussing their plan of care  *Then* the more likely it is that treatment is going to be effective | Academic |
| 247 | Ungar et al. (2012) p. 14 (#521) | *The provision of a continuum of services from least to most intrusive is critical to the management of complex cases* | Academic |
| 248 | Bodenheimer et al. (2002) p. 1775 (#579) | *CHRONIC CARE MODEL (CCM) – Wagner*  *If system is designed such that acute care takes precedent in primary care then chronic disease care suffers, with patients not adequately taught to care for their own illnesses, visits are brief and little planning takes place to ensure that acute and chronic needs are addressed. A division of labour that allows non-physician personnel to take greater responsibility in chronic care management is proposed. CCM describes a health care system including many (all?) of the principles that Engager II strives to create.*  RB and CQ would like this included as a theoretical background paper | Academic |
| 249 | Parsonage (2009) p. 56 (#524) | *A mental health worker from diversion/liaison team could attend probation meetings as a way of encouraging and monitoring an offender’s engagement with services* (DP about how our practitioner can do CM in community) | Policy maker / third sector |
| 250 | Durcan (2008) p. 7 (#533);  Whittle et al. (2012) (#445) | *Prisoners say that they need something to do during the day: meaningful activity, including work and exercise.* | Policy maker / third sector |
| 251 | M. M. Cornes, Jill; Hennessy, Catherine; Anderson, Sarah (2013) p.3 (#555) | *ILT and shared case management can be supported by Communities of Practice. They outline how they set up Communities of Practice and the benefits of these. (Could be something our practitioner works to create to support cases.)* | Policy maker / third sector |
| 252 | Forrest (2013) p. 1, 2 (#564) | *If* there is a strong prison culture (the inmate code: don’t grass, do your own time, man up; hierarchy of offenders rigid)  *And i*t is reinforced in the community (outside prison culture may support this prison culture to greater or lesser extent)  *Then* this can militate against many of the approaches used in psychological therapies, such as being aware of and open about emotions. | Practitioner |
| 253 | Forrest (2013) p. 1, 2 (#564) | *If* inappropriate accommodation; risk and safety; individual’s culture and lack of emotional vocabulary; inability to trust; anxiety about confidentiality; are present for an offender or if the therapist has to provide a report for parole boards, offender managers, and other bodies  Then these can make the establishment of a strong therapeutic alliance difficult | Practitioner |
| 254 | Forrest (2013) p. 1, 2 (#564) | *Practitioners in prisons need training on issues of comorbidity, dual diagnosis, PD, self-harm, dealing with literacy problems and learning disabilities, risk and safety. Extra emotional load of working with this population requires above average levels of supervision.* | Practitioner |
| 255 | Lad (2013b) p. 3 (#565) | *Important to complete psycho-education and case formulation understanding early life events rather than basic hot cross buns* | Practitioner |
| 256 | Lad (2013) p. 3 (#565)  Lad (2013a) (#818) | *Repeated trauma having an overlap with offending behaviour can be intervened with using a CBT approach* | Practitioner |
| 257 | Anderson (2011) p. 9 (#558) | *If* there is a failure to communicate with other agencies so as to coordinate care  *Then* offenders will be put under the stress of repeated assessments and resulting care plans that fail to identify the ‘depth’, ‘breadth’, and interconnectedness of need | Policy maker / third sector |
| 258 | Anderson (2011) p. 10 (#558) | *If* there is stigma of people with multiple needs (such as demanding or manipulative) by health professionals  *Then* this is a barrier to change in treatment of people with multiple needs | Policy maker / third sector |
| 259 | Repper (2013) p. 8, 9 (#544) | *Peer support – some of main principles: 1) recovery-focused. This includes: inspiring hope by helping to generate personal belief, energy and commitment; supporting people to take back control of their personal challenges and face their own destiny; facilitating access to opportunities that the person values, enabling them to participate in roles, relationships and activities in the communities of their choice. 2) strengths based, including: being with someone in their distress, but also seeing within that distress the seeds of possibility and creating a fertile ground for those seeds to grow. Explores what a person gained from experience, seeks out their qualities and assets, identifies hidden achievements and celebrates what may seem like the smallest steps forward.* | Policy maker / third sector |
| 260 | Saitz et al. (2008) p. 2/3 (#599) | *If* addiction is seen as a chronic disease with high rates of comorbidity requiring long-term treatment  Then this will change treatment from short-term interventions or detoxification and no follow up to an integrated system of care focusing on long-term management of substance dependence | Academic |
| 261 | Alexander et al. (2007) p. 221 (#600) | *If* more active case management is used during the referral process  *And* case management is provided both on and off site  *Then* there is greater use of health and ancillary social services by substance misuse clients | Academic |
| 262 | Strauss (2006) p. 64 & 69 (#609) | *If* low to medium-threshold services taking a risk reduction rather than an abstinence approach engage the user for at least six months in services that require very little effort for user and assist them in developing treatment readiness prior to treatment referral  *then* drug users have higher levels of readiness for longer-term and higher-threshold services  *Then* they will have greater involvement in a treatment programme  *And* therefore a greater level of success | Policy maker |
| 263 | Ales et al. (2011) p. S17 (#621) | *If* a collaboration studies whether desired results are achieved through outcomes measurements integrated in to each activity  *And* the findings are fed back to the collaborators  *Then* collaborators will be more engaged in collaboration and its aims  *And* collaboration will be more effective at reaching its goals | Academic |
| 264 | Ales et al. (2011) p. S17 (#621) | *If* case formulation involves risk management strategies so that one resource, person, or organisation is not relied on too heavily  *Then* collaboration is more likely to be sustainable | Academic |
| 265 | Ales et al. (2011) p. S18 (#621) | *If* a collaborative shares a common purpose that is clearly articulated to each member  *And* that includes attainable goals that are unique from but related to the mission of any single organisation in collaborative  *And* there is a mixture of short-term and long-term goals that allow for quick wins and extended motivation  *Then* individual members of collaborative will be engaged and motivated to continue to participate in collaboration | Academic |
| 266 | Vanderplasschen et al. (2004) p.915 (#624) | *If* case management includes extensive training and supervision to foster collaboration and precontracting of services to ascertain their availability  *And* there are formal agreements and protocols concerning the tasks, responsibilities, and authorities of case managers and other service providers involved; the use of common assessment and planning tools; and exchange and management of client information  *Then* cooperation and coordination between services can be enhanced | Academic |
| 267 | Vanderplasschen et al. (2004) p.919 (#624) | *If* there is client-driven goal setting facilitated by case manager’s assistance in teaching clients how to set goals  *Then* retention will be improved and thus case management will be more effective | Academic |
| 268 | Vanderplasschen et al. (2004) p.919 (#624) | *If* the roles of case manager and counsellor are combined  *Then* this dilutes both aspects of the program | Academic |
| 269 | Vanderplasschen et al. (2004) p.919 (#624) | *If* a strengths-based approach is used  And there is a strong client-case manager relationship  *Then* clients’ involvement can be stimulated | Academic |
| 270 | Vanderplasschen et al. (2004) p.919 (#624) | *If* there is extensive initial training, regular supervision, admin support, application of protocols and manuals, treatment planning, and a team approach  *Then* fidelity and implementation of case management can be optimised | Academic |
| 271 | Vanderplasschen et al. (2004) p.920 (#624) | Individual case managers’ personalities, client characteristics, motivation, legal status, and treatment participation and retention will have an effect on the direct impact of case management of client functioning | Academic |
| 272 | Vanderplasschen et al. (2004) p.920 (#624) | *If* case management involves integration of the program in a comprehensive network of services, accessibility and availability, provision of direct services, use of a team approach, application of a strengths-based perspective, intensive training, and regular supervision  *Then* implementation of case management will be improved  *and* thus there will be more beneficial outcomes | Academic |
| 273 | Amaro et al. (2007) p. 508 & 512 (#630) | *If* a trauma-informed approach is integrated in to drug treatment and mental health services for women with trauma histories (including trauma and MH assessment, treatment planning, and case management; 25 session trauma recovery and empowerment model group; and manualised trauma-informed skills-building groups)  *Then* there are significantly better outcomes in drug abstinence rates and MH symptoms and PTSD | Academic |
| 274 | Allsop & Stevens (2009) p. 545 (#635) | *If* developing expertise or attitudes of individuals is done without addressing organisational factors  *Then* it is likely to have limited impact and may even create frustration and will be much less productive than broad organisation/system approaches | Academic |
| 275 | Thompson (2000) p. 6 (#637) | *If* patients are pulled in to postacute care by a formal specifying of which clinicians (both inpatient and outpatient providers) are responsible for which elements of discharge planning and then following up with patient shortly after discharge  *Then* patient transitions are significantly improved and the number who fall through the cracks significantly reduced | Academic |
| 276 | Brown et al. (2004) p.93 (#674) | *If* AOD, MH, welfare, criminal justice, homelessness, and health services are all aware that they all intersect around the co-occurring issue of trauma  *Then* this can educate all providers to become more trauma-sensitive and provide services which are integrated and more trauma-informed | Academic |
| 277 | Smith & Mogro-Wilson (2007) p.554 (#677) | *If* staff are trained in inter-agency collaborative practice or helped to see potential benefits of collaborative practice  *Then* staff will have more positive perceptions of inter-agency collaboration and more knowledge about it  *Then* staff will be more likely to collaborate | Academic |
| 278 | Urada et al. (2012) p.297 (#687) | *If* there are discussions between staff of the current patterns and expectations of communications and how processes might be improved  *Then* this may promote improved integration and provider satisfaction | Academic |
| 279 | J. Martin (2006) p.17 (#709) | *If* health and social care professionals are co-located  *Then* the extent of partnership working is increased and integrated care is improved | Practitioner |
| 280 | Salman (2011) p.11 (#711) | *If* care budgets are ‘individualised’ (i.e. decisions about how to spend are made by recipients)  *Then* the matrix of services provided will match individuals’ needs and wants more closely | Practitioner |
| 281 | Salman (2011) p.16 (#711) | *If* the relationship between commissioners, providers and service users is balanced  *Then* personalised support is enabled | Practitioner |
| 282 | Miles (2009) p.342 (#712) | *If* structured, succinct, standardised forms which are easily distinguishable from other records (e.g. by colour) are used  *Then* continuity of care is enhanced | Academic |
| 283 | Berkowitz et al. (1992) p.104 (#751) | *If* the means of accessing services (identifying, obtaining services from, maintaining contact with, co-ordinating with other services) is unclear and/or complicated  *Then* a case manager can assist service users to effectively negotiate the system and gain access to services | Academic |
| 284 | Weinstein et al. (2013) p.285 (#785) | *If* funding is aligned with strategic goals to integrate services  *Then* progress towards service integration is facilitated | Academic |
| 285 | Rosenheck et al. (2001) p.706 (#793) | *If* trust, co-operation and co-ordination between practitioners can be fostered by systems integration and social capital of practitioners  *Then* the performance of service delivery agencies is improved and user health outcomes are improved | Academic |
| 286 | Shelter (2007) p.4 (#794) | *If* cross-cutting targets *across* organisations can be agreed and supported by pooled budgets  *Then* services could be more inclusive of street homeless people with a dual diagnosis | Other |
| 287 | Shelter (2007) p.4 (#794) | *If* formal partnerships between organisations (drug and alcohol, housing and homelessness, mental health) can be created  *and* multi-agency protocols developed in consultation with all stakeholders  *and* these are widely promoted, maintained and reviewed by all participating organisations  *Then* services for street homeless people with a dual diagnosis will be improved | Other |
| 288 | Milaney (2012) p.5 (#704) | *If* a multidisciplinary team is diverse (in terms of gender, culture, etc.)  *Then* clients will have greater opportunity to develop relationships with people who they have a particular connection with | Academic |
| 289 | Milaney (2012) p.5 (#704) | *If* there is clarity about the roles within, boundaries between, and mutual goals of teams  *Then* integrated working is facilitated | Academic |
| 290 | Milaney (2012) p.5 (#704) | *If* the lead case manager holds multidisciplinary team members accountable, and coordinates meetings and communication for all teams  *Then* integrated working is facilitated | Academic |
| 291 | Markoff et al. (2005) p.236 (#695) | *If* after a year of cross-training staff to understand the interaction between trauma, mental health and drug misuse the staff feel that they are more knowledgeable but that their actual practice has not changed  *Then* having an expert on integrated treatment available to the staff for a couple of hours a month for supervision or discussion of cases or system issues related to integrated care will support the movement from knowledge to practice for the staff. | Practitioner |
| 292 | Dillard et al. (2010) p. 137 (#653) | *If* employment readiness is targeted  *Then* job prospects and poverty risk are improved | Academic |
| 293 | Kirchner et al. (2004) p. 21 (#645) | *If* there is an understanding of the culture and context of the community, the health care setting, and the interaction between the two  *Then* there will be greater success is adaptation and adoption of health care innovations | Academic |
| 294 | Nebelkopf & Penagos (2005) p. 262/3 (#644) | *If* there is the capacity to build community amongst disenfranchised HIV+ natives, including the promotion of positive mental health, self-responsibility, and commitment to the family  *Then* success of integrated services in this group is more likely | Academic |
| 295 | Kipping et al. (2011) p. 232 (#638) | *If* a move is made from ‘health needs assessment’ to an opening up of the conceptual framework to focus on ‘health’, ‘wellbeing’, and ‘dignity’  *Then* this will enable the partnership board to focus on the prison as a whole system, and to identify aspects of the environment and culture which may enhance or detract from the promotion of health in its widest sense | Academic |
| 296 | Kim et al. (2004) p.110 (#701) | *If* a philosophy of care emphasises individualisation of interventions, empowerment of service users, is strengths-based, and the service user works ‘in concert’ with the support team and sets the priorities and timing of services  *Then* a holistic service that improves health outcomes is attained | Academic |
| 297 | Kim et al. (2004) p.114 (#701) | *If* treatment plans foster coping and decision-making capabilities, self-advocacy, self-care, and sustainable support systems that will continue after professional services have ended  *Then* a holistic service that improves health outcomes is attained | Academic |
| 298 | Kim et al. (2004) p.114 (#701) | *If* a forum is provided for care professionals to meet regularly to refine their skills, receive support and feedback, and improve their own network of resources  *Then* skill development is enabled and supported | Academic |
| 299 | Cameron (2006) (#735) | *If* there is ‘buy-in’ to joint working at both a strategic and operational level  *And* there is ongoing two-way communication between the levels  *Then* collaborative, cross-sector working is enabled | Academic |
| 300 | Cameron (2006) (#735) | *If* governance and management arrangements, and professionals’ responsibilities and boundaries of working are clear  *Then* collaborative, cross-sector working is enabled | Academic |
| 301 | M. Cornes et al. (2014) p.140 (#823) | *If* short-term ‘recovery’ outcomes are prioritised over longer-term ‘maintenance and prevention’ outcomes  *Then* service-user centred goals and integrated working are inhibited | Academic |
| 302 | M. Cornes et al. (2014) p.141 (#823) | *If* commissioning of services emphasises ‘personal budgets’ over large-scale projects (such as Housing First)  *Then* the opportunity of support within secure accommodation is put at risk | Academic |
| 303 | McNeill & Weaver (2010) p.6 (#824) | *If* offenders’ efforts to desist from crime are recognised and rewarded by practitioners  *Then* reintegration in communities is promoted | Academic |
| 304 | McNeill & Weaver (2010) p.7 (#824) | *If* work with offenders is conducted on the basis that desistance from crime is a (potentially long) journey  *and* offenders are facilitated to understand their journey in its social and cultural context  *and* offenders’ significant others are engaged as partners on the journey  *and* workers retain sufficient flexibility to increase or decrease their support at different stages of the offenders’ journey  *Then* desistance from crime and community reintegration is facilitated | Academic |
| 305 | McNeill & Weaver (2010) p.7 (#824) | *If* relationships between practitioners and offenders are of sufficient quality (trust, respect, confidence)  *Then* this underpins all other aspects of the supervision process (i.e. it is necessary, but not sufficient) | Academic |
| 306 | McNeill & Weaver (2010) p.64-70 (#824) | *If* practitioners’ interactions with offenders are not sensitive to the importance of core aspects of offenders’ identities (gender, ethnicity, religion/spirituality)  *Then* an individualised, strengths-based approach to desistance is hindered | Academic |
| 307 | LeBel et al. (2008) p.154 (#831) | *If* offenders feel stigmatised  *Then* desistance is hindered  (and conversely)  *If* offenders have a positive self-image, e.g. of having ‘moved on’ from crime to being a ‘family man’  *Then* desistance is enabled | Academic |
| 308 | LeBel et al. (2008) p.154 (#831) | *If* a person has an ‘adequate sense of hope’  *Then* they are more likely to both take advantage of positive social opportunities (e.g. employment, marriage) and ‘weather disappointments or setbacks in these areas (i.e. greater resilience) | Academic |
| 309 | McNeill (2006) p.47 (#832) | The *meaning* that life changes and events have for individual offenders outweighs the general impact of these changes/events | Academic |
| 310 | Vaughan (2007) p.401 (#833) | An offenders’ ability to change their behaviour pivots around their ability to see a ‘past self’ and a desire ‘future self’, and an ability to ‘grab’ a ‘hook for change’ when it arises. Practitioners and significant others can play a substantial role by ‘holding up a mirror to the offenders sense of self’ | Academic |
| 311 | Horsfall et al. (2009) p. 27 (#693) | Model for motivation (Prochaska and DiClemente) - Five stages of readiness in substance abuse population: precontemplation, contemplation, preparation, action, maintenance | Academic |
| 312 | Horsfall et al. (2009) p. 27 (#693) | *If* motivational interviewing is used by the therapist of people with substance abuse issues  *Then* people with substance abuse issues can be supported to move from precontemplation to contemplation | Academic |
| 313 | Horsfall et al. (2009) p. 27 (#693) | *If* people with dual diagnosis of mental health issues and drug misuse have family support from people who remain involved  *Then* this may enhance both group and individual approaches, and have a significant impact on clinical outcomes and recovery | Academic |
| 314 | Horsfall et al. (2009) p. 27 (#693) | *If* interventions are tailored to an individual’s readiness to change stages  *And* there is continual assessment and reassessment of their motivation to change  *Then* treatment of dually diagnosed mental health issues and drug misuse issues will be more effective | Academic |
| 315 | Draine & Herman (2007) p. 1577 (#619) | CTI is a nine month three stage intervention for people with mental health problems leaving prison to strategically develop individualised linkages in the community and seeks to enhance engagement with treatment and community supports through building problem-solving skills, motivational coaching, and advocacy with community agencies. It emphasises the role of community ties in individual and social outcomes | Academic |
| 316 | Draine & Herman (2007) p. 1577 (#619) | *If* temporary support is provided as a person with mental health issues leaving prison re-builds their community living skills  *And* they are supported to develop a persisting network of community ties  *Then* this will support long-term recovery and reintegration into the community | Academic |
| 317 | Draine & Herman (2007) p. 1579 (#619) | *If* ties in community are increased  Then social capital will increase  Then outcomes for person with MH issues leaving prison will be improved | Academic |
| 318 | Draine & Herman (2007) p. 1580 (#619) | *If* there is community impoverishment  *Then* involvement in the criminal justice system increases and prosocial outcomes decrease among people in urban communities | Academic |
| 319 | Draine & Herman (2007) p. 1580 (#619) | *If* people leaving prison with mental health issues are connected to sustainable formal and informal relationships in the community  *Then* offender wellbeing will be increased, and hopefully also recidivism | Academic |
| 320 | Daniels et al. (2009) p. 108 (#612) | The chronic care model of Wagner’s needs to be modified for behavioural health. Including the addition of social inclusion and opportunities, which impact the outcomes of health systems, coordinated medical and community-based care and person-centred recovery | Academic |
| 321 | Karoll (2010) p. 265 (#589) | *If* practitioners practice in a way that assists people to empower themselves  *Then* this will facilitate the attainment of knowledge, skills, and resources of an emotional and material nature  *And then* this will support them to achieve meaningful social roles |  |
| 322 | Karoll (2010) p. 266 (#589) | *If* people misusing drugs are seen by practitioners from a viewpoint of their capacities and capabilities, competencies, possibilities, talents, visions, hopes, and values, regardless of how altered and shattered they may have become due to their circumstances, trauma, and oppression  *And if* practitioners see them as capable of making significant progress in facing their difficulties when helped to reawaken their personal abilities  *And if* practitioners see potential and possibilities rather than problems, options and choices rather than constraints, and wellness rather than sickness  *Then* clients’ strengths, resources, and environment will remain the central focus of the helping process  *And then* practitioners will support the clients’ ability to creatively draw upon their own strengths and community resources to cope in ways that promote self-efficacy and community integration | Academic /  Person with lived experience |
| 323 | Karoll (2010) p. 270 (#589) | *If* initial contact with a person with drug dependency includes listening attentively and reflecting back their story  *And* it includes offering choices for meeting times that are outside 9 – 5 hours (flexibility in availability)  *Then* initial engagement in intervention will be improved | Academic /  Person with lived experience |
| 324 | Karoll (2010) p. 270 (#589) | *If* before initial contact with a person with drug dependency they are asked to write out their expectations of therapy and their treatment goals to be discussed at the first session  *Then* this will impress on them that what they say and want to accomplish are of prime concern and the driving force of the relationship  *And then* their compliance will also inform the practitioner where they are on the stages of change  *And then* whether to employ experiential approaches to enhance motivation or to initiate behavioural interventions | Academic /  Person with lived experience |
| 325 | Karoll (2010) p. 271 (#589) | *If* practitioners eliminate all negative words and phrases, that is, denial, resistance, unmotivated, uncommitted, and labels associated with these disorders (e.g. reframe defence mechanisms as care strategies)  *Then* this will support them in showing the client that they are suspending all judgement around alcohol or drug use | Academic /  Person with lived experience |
| 326 | Karoll (2010) p. 271 (#589) | *If* practitioners convey that all clients possess various competencies and resources they may tap in to during the assessment  *Then* this offers hope and provides initial motivation to fully engage in treatment process | Academic /  Person with lived experience |
| 327 | Karoll (2010) p. 271 (#589) | *If* practitioners offer hope and reframing to their clients during the assessment  *Then* this allows for the development of attitudes and language about the nature of opportunity and possibility | Academic /  Person with lived experience |
| 328 | Karoll (2010) p. 271 (#589) | The assessment process provides an early and ongoing opportunity for the client/social worker partnership to name and rename the problem, shifting perspectives from deficits to strengths and providing the client opportunities to have voice in shaping the method for problem remediation. | Academic /  Person with lived experience |
| 329 | Karoll (2010) p. 271 (#589) | *If* the practitioner has clients report their current stressors and, on a scale of 1 – 10 , how serious they see their current stress level  *Then* this aids in determining clients’ impressions of their problems and their probable stage of change | Academic |
| 330 | Karoll (2010) p. 274 (#589) | *If* a practitioner assists clients in learning to express their feelings early in the helping relationship  *Then* this helps clients become aware that they have actual control over how they feel by changing what they think about  *And then*, by accomplishing this during a session, gives them cause to rejoice about their tangible therapeutic success and strengthens their hope that full recovery is possible | Academic |
| 331 | Heckman et al. (2004) p.177 (#588) | *If* practitioners know the treatment barriers (e.g. transportation, childcare)  *And* provide external client supports (e.g. reminder phone calls, transportation, childcare)  *And* engage people in services immediately following their interest  *And* has an understanding of client treatment readiness  *Then* client engagement in services will be increased | Academic |
| 332 | Kavanagh & Borrill (2013) p. 411 (#845) | *If* ex-offenders act as mentors to other offenders  Then they feel empowered, are enabled to continue on a path of desistance by having an opportunity to give back to society, are encouraged in their personal and professional growth and increasing self-esteem, develop an array of interpersonal skills, benefit from building trusting and open relationships with their mentees, and are kept grounded, being reminded in their mentoring activities of paths they never want to walk down again, such as homelessness, addiction, and criminal behaviour, and their own positive changes are reinforced. | Academic |
| 333 | Bensimon et al. (2013) p.1 (#846) | *If* relaxing music is played to men in prison  Then they experience less state anxiety and state anger | Academic |
| 334 | Holtfreter & Wattanaporn (2014) (#848) | Transition from Prison to Community Initiative and Transitional Accountability Plan (TAP). Very similar approach to our intervention, with the TAP being the rolling assessment and care plan. | Academic |
| 335 | Johnson (2013) p.41 (#571) | *If* there is re-housing for vulnerable people  *Then* this will have a distinct positive effect on their mental health | Academic |
| 336 | Johnson (2013) p.42 (#571) | *If* interventions are conscious of the multi-factorial linkages and benefits between social capital, satisfactory housing, welcoming features in neighbourhoods, and a sense of well-being  *Then* they are more likely to bring about change in each of these areas | Academic |
| 337 | Johnson (2013) p.42 (#571) | *If* vulnerable people have a greater choice and/or sense of control over the immediate environment (homes/neighbourhoods)  *Then* they will have greater satisfaction and greater well-being | Academic |
| 338 | Reilly et al. (2010) p.139 (#834) | *If* the case manager role is combined with other clinical responsibilities  *Then* the availability of time and resources for effective case management is reduced | Academic |
| 339 | Reilly et al. (2010) p.139 (#834) | *If* case managers do not have influence over the form and content of services  *Then* case management is impeded | Academic |
| 340 | Reilly et al. (2010) p.147 (#834) | *If* management of caseload size takes account of different need profiles and absolute numbers  *Then* effective case management is enabled | Academic |
| 341 | Challis et al. (2002) p.323 (#837) | *If* case managers have the resources (i.e. budgetary control) to respond flexibly (e.g. creating own services, buying-in independent sector services, using existing health/social service resources)  *Then* effective case management is enabled | Academic |
| 342 | Ward (2002b) p.173-4 (#850);  Ward (2002a) (#851);  Ward & Brown (2004) (#852);  Ward et al. (2007) (#854) | A ‘good lives’ conceptualisation of offender rehabilitation (an approach based on offenders’ strengths rather than deficits (risk factors)) revolves around:  1. ‘Primary goods’ – valued human activities and experiences  2. Identity – people pursue meaningful activity as part of their identity  3. Wellbeing – the pursuit of ‘goods’ necessary for wellbeing (e.g. intimacy, health, autonomy, creativity, knowledge)  4. Individual contexts – and how adaptive and coping skills are closely linked to these  5. A treatment plan that is explicitly constructed around the pursuit of positive goals  Positive activities can contribute to an individual’s autonomy, relatedness (intimate, romantic, family), and sense of competence (in play and work). | Academic |
| 343 | Jaffe (2012) (#316) | *If* prisoners have the opportunity to discuss their anxieties with a peer who is skilled in listening  *Then* a negative spiral of anxiety and depression can be arrested  *And* a firmer foundation laid for wellbeing | Offender |
| 344 | Le Boutillier et al. (2011) p.1474 (#359) | *If* people in recovery from mental ill-health share their own stories and model empowerment  *Then* this can help others to improve their self-management skills and ability to take personal responsibility | Academic |
| 345 | Bateman & Krawitz (2013) p.176 (#919) ;  Fonagy et al. (2012) p.37 (#921) | *If* a person can be enabled to become aware of and think in a non-judgemental way about their own thoughts, emotions and actions  *And* a balance can be struck between intellectual analysis and emotional involvement  *Then* a person’s *relationship* with their thoughts and emotions can be improved (even if the thoughts and emotions remain unchanged)  *And* the foundations are laid for a person to decide how they may wish to act differently in the future | Academic |
| 346 | Bateman & Fonagy (2012b) p.xvi (#920) | *If* “a patient feels that his or her subjective states of mind are understood”  *Then* “they are more likely to be receptive to therapeutic intervention” | Academic |
| 347 | Fonagy et al. (2012) p.34 (#921);  Fonagy & Bateman (2006) p.424 (#923) | *If* a safe and sensitive interpersonal environment can be created  *Then* there is space for a person to focus on reflection and ‘mentalisation’ whilst regulating their affect | Academic |
| 348 | Fonagy et al. (2012) p.37 (#921);  Bateman & Fonagy (2012a) p.68 (#922);  Fonagy and Bateman (2007) p.93 (#925) | Mentalisation (understanding why oneself and others do what they do) ‘works’ by:   - 1. stimulating a joint consideration of underlying processes (rather than the therapist ‘knowing’ and the person ‘receiving’ this knowledge) - 2. acknowledging and exploring the different components of thought processes (rather than the therapist showing a person their ‘inaccuracy’)   3. helping a person to attend to his or her own feelings (rather than identifying and naming these) | Academic |
| 349 | Jordan (2012) p. 730 (#250) | *If* an offender perceives that there is an absence of care in the general prison environment  *And* that in the prison environment they are not in a position of power in relation to other people  *And* the relationship with a practitioner is perceived as caring and equalising the power differential  *Then* the relationship is additionally therapeutic | Academic |

**Note:** Explanatory account numbering reaches a higher number than the total number of explanatory accounts due to non-consecutive numbering (#184 and #185 were missed)

Ales, M.W., Rodrigues, S.B., Snyder, R., & Conklin, M. (2011). Developing and implementing an effective framework for collaboration: the experience of the CS2day collaborative. *Journal of Continuing Education in the Health Professions,* 31 Suppl 1, S13-20.

Alexander, J.A., Pollack, H., Nahra, T., Wells, R., & Lemak, C.H. (2007). Case management and client access to health and social services in outpatient substance abuse treatment. *Journal of Behavioral Health Services & Research,* 34, 221-236.

Allsop, S.J., & Stevens, C.F. (2009). Evidence-based practice or imperfect seduction? Developing capacity to respond effectively to drug-related problems. *Drug & Alcohol Review,* 28, 541-549.

Amaro, H., Dai, J., Arevalo, S., Acevedo, A., Matsumoto, A., Nieves, R., et al. (2007). Effects of integrated trauma treatment on outcomes in a racially/ethnically diverse sample of women in urban community-based substance abuse treatment. *Journal of Urban Health,* 84, 508-522.

Anderson, S. (2011). Complex Responses: Understanding poor frontline responses to adults with multiple needs: A review of the literature and analysis of contributing factors. Revolving Doors.

Backhouse, M. (2007). NHS walk-in centres - A potent catalysist for developing prison healthcare. *International Journal of Prisoner Health,* 3, 79-81.

Barber, M., Short, J., Clarke-Moore, J., Lougher, M., Huckle, P., & Amos, T. (2006). A secure attachment model of care: meeting the needs of women with mental health problems and antisocial behaviour. *Criminal Behaviour and Mental Health,* 16, 3-10.

Bateman, A.W., & Fonagy, P. (2012a). Individual techniques of the basic model. In A.W. Bateman, & P. Fonagy (Eds.), *Handbook of mentalising in mental health practice* pp. 67-80). Washington. DC: American Psychiatric Publishing, Inc.

Bateman, A.W., & Fonagy, P. (2012b). Preface. In A.W. Bateman, & P. Fonagy (Eds.), *Handbook of mentalizing in mental health practice* pp. xv-xxi). Washington, DC: American Psychiatric Publishing, Inc.

Bateman, A.W., & Krawitz, R. (2013). *Borderline Personality Disorder: An evidence-based guide for generalist mental health professionals*. Oxford: Oxford University Press.

Bensimon, M., Einat, T., & Gilboa, A. (2013). The Impact of Relaxing Music on Prisoners’ Levels of Anxiety and Anger. *International Journal of Offender Therapy and Comparative Criminology*, 0306624X13511587.

Berkowitz, G., Halfon, N., & Klee, L. (1992). Improving Access to Health Care: Case Management for Vulnerable Children. *Social Work in Health Care,* 17, 101-123.

Bodenheimer, T., Wagner, E.H., & Grumbach, K. (2002). Improving primary care for patients with chronic illness. *JAMA,* 288, 1775-1779.

Brooker, C., & Birmingham, L. (2009). The psychiatric aspects of imprisonment revisited. *The Journal of Forensic Psychiatry & Psychology,* 20, S1-S4.

Brooker, C., Gojkovic, D., Sirdifield, C., & Fox, C. (2009). Lord Bradley’s review of people with mental health or learning disabilities in the criminal justice system in England: All not equal in the eyes of the law? *International Journal of Prisoner Health,* 5, 171-175.

Brough, P., & Williams, J. (2007). Managing Occupational Stress in a High-Risk Industry: Measuring the Job Demands of Correctional Officers. *Criminal Justice and Behavior,* 34, 555-567.

Brown, V.B., Rechberger, E., & Bjelajac, P. (2004). A Model for Changing Alcohol and Other Drug, Mental Health, and Trauma Services Practice: Prototypes Systems Change Center. *Alcoholism Treatment Quarterly,* 22, 81-94.

Buetow, S. (2007). What motivates health professionals? Opportunities to gain greater insight from theory. *Journal of Health Services Research & Policy,* 12, 183-185.

Byng, R., Quinn, C., Sheaff, R., Samele, C., Duggan, S., Harrison, D., et al. (2012). COCOA: Care for Offenders, Continuity of Access. *Final report. NIHR Service Delivery and Organisation programme*.

Calnan, M., & Rowe, R. (2008). Trust relations in a changing health service. *Journal of Health Services Research & Policy,* 13, 97-103.

Cameron, A. (2006). An evaluation of the Supporting People Health Pilots.

Caulfield, L.S., & Twort, H. (2012). Implementing change: staff experiences of changes to prison mental healthcare in England and Wales. *International Journal of Prisoner Health,* 8, 7-15.

Chafin, W.S., & Biddle, W.L. (2013). Nurse Retention in a Correctional Facility: A Study of the Relationship Between the Nurses’ Perceived Barriers and Benefits. *Journal of Correctional Health Care,* 19, 124-134.

Challis, D., Von Abendorff, R., Brown, P., Chesterman, J., & Hughes, J. (2002). Care management, dementia care and specialist mental health services: An evaluation. *International Journal of Geriatric Psychiatry,* 17, 315-325.

Chew-Graham, C., Slade, M., Montâna, C., Stewart, M., & Gask, L. (2008). Loss of doctor-to-doctor communication: lessons from the reconfiguration of mental health services in England. *Journal of Health Services Research & Policy,* 13, 6-12.

Collins, P., & Barker, C. (2009). Psychological help-seeking in homeless adolescents. *International Journal of Social Psychiatry,* 55, 372-384.

Cornes, M., Manthorpe, J., Joly, L., & O'Halloran, S. (2014). Reconciling recovery, personalisation and Housing First: Integrating practice and outcome in the field of multiple exclusion homelessness. *Health & Social Care in the Community,* 22, 134-143.

Cornes, M.M., Jill; Hennessy, Catherine; Anderson, Sarah (2013). LITTLE MIRACLES: Using Communities of Practice to improve front line collaborative responses to multiple needs and exclusions. Revolving Doors and Kings College London.

Crawford, M., & Rutter, D. (2007). Lessons Learned from an Evaluation of Dedicated Community-based Services for People with Personality Disorder. *Mental Health Review Journal,* 12, 55-64.

Curd, P.R., Winter, S.J., & Connell, A. (2007). Participative Planning to Enhance Inmate Wellness: Preliminary Report of a Correctional Wellness Program. *Journal of Correctional Health Care,* 13, 296-308.

Daniels, A.S., Adams, N., Carroll, C., & Beinecke, R.H. (2009). A conceptual model for behavioral health and primary care integration. *International Journal of Mental Health,* 38, 100-112.

Davies, H., Powell, A., & Rushmer, R. (2007). Why don't clinicians engage with quality improvement? *Journal of Health Services Research & Policy,* 12, 129-130.

De Viggiani, N. (2006). Surviving Prison: Exploring prison social life as a determinant of health. *International Journal of Prisoner Health,* 2, 71-89.

DeHart, D.D., Smith, H.P., & Kaminski, R.J. (2009). Institutional Responses to Self-Injurious Behavior Among Inmates. *Journal of Correctional Health Care,* 15, 129-141.

Devilly, G.J., Sorbello, L., Eccleston, L., & Ward, T. (2005). Prison-based peer-education schemes. *Aggression and Violent Behavior,* 10, 219-240.

Dhaliwal, R., & Harrower, J. (2009). Reducing prisoner vulnerability and providing a means of empowerment: evaluating the impact of a Listener Scheme on the Listeners. *British Journal of Forensic Practice, The,* 11, 35-43.

Dillard, D., Bincsik, A.K., Zebley, C., Mongare, K., Harrison, J., Gerardi, K.E., et al. (2010). Integrated nested services: Delaware's experience treating minority substance abusers at risk for HIV or HIV positive. *Journal of Evidence-Based Social Work,* 7, 130-143.

Draine, J., & Herman, D.B. (2007). Critical time intervention for reentry from prison for persons with mental illness. *Psychiatric Services,* 58, 1577-1581.

Duncan, E.A.S., Nicol, M.M., Ager, A., & Dalgleish, L. (2006). A systematic review of structured group interventions with mentally disordered offenders. *Criminal Behaviour and Mental Health,* 16, 217-241.

Durcan, G. (2008). *From the inside: experiences of prison mental health care*: Sainsbury Centre for Mental Health.

Emlyn-Jones, R. (2007). Think about it till it hurts: targeting intensive services to facilitate behaviour change – two examples from the field of substance misuse. *Criminal Behaviour and Mental Health,* 17, 234-241.

Ferguson, G., Conway, C., Endersby, L., & MacLeod, A. (2009). Increasing subjective well-being in long-term forensic rehabilitation: evaluation of well-being therapy. *The Journal of Forensic Psychiatry & Psychology,* 20, 906-918.

Feron, J.-M., Tan, L.H.N., Pestiaux, D., & Lorant, V. (2008). High and variable use of primary care in prison. A qualitative study to understand help-seeking behaviour. *International Journal of Prisoner Health,* 4, 146-155.

Fonagy, P., & Bateman, A.W. (2006). Mechanisms of change in mentalization-based treatment of BPD. *Journal of Clinical Psychology,* 62, 411-430.

Fonagy, P., & Bateman, A.W. (2007). Mentalizing and boderline personality disorder. *Journal of Mental Health,* 16, 83-101.

Fonagy, P., Bateman, A.W., & Luyten, P. (2012). Introduction and overview. In A.W. Bateman, & P. Fonagy (Eds.), *Handbook of mentalizing in mental health practice* pp. 3-42). Washington, DC: American Psychiatric Publishing, Inc.

Forrest, J. (2013). Developing and Delivering Services which Respond to the Unique Needs of Offenders. HMP/YOI Swinfen Hall (Inside Help IAPT Service), Derbyshire Healthcare NHS Foundation Trust.

Fortune, Z., Rose, D., Crawford, M., Slade, M., Spence, R., Mudd, D., et al. (2010). An evaluation of new services for personality-disordered offenders: Staff and service user perspectives. *International Journal of Social Psychiatry,* 56, 186-195.

Fraser, A. (2009). Mental health in prisons: A public health agenda. *International Journal of Prisoner Health,* 5, 132-140.

Goldstein, E.H., Felizardo, V., Conklin, R.M., & Schissel, R. (2006). A Mental Health Clinician Model of Care in a Jail System. *Journal of Correctional Health Care,* 12, 189-202.

Goodwin, N. (2008). Are networks the answer to achieving integrated care? *Journal of Health Services Research & Policy,* 13, 59-60.

Griffin, M.L., Hogan, N.L., & Lambert, E.G. (2012). Doing “People Work” in the Prison Setting: An Examination of the Job Characteristics Model and Correctional Staff Burnout. *Criminal Justice and Behavior,* 39, 1131-1147.

Griffin, M.L., Hogan, N.L., Lambert, E.G., Tucker-Gail, K.A., & Baker, D.N. (2010). Job Involvement, Job Stress, Job Satisfaction, and Organizational Commitment and the Burnout of Correctional Staff. *Criminal Justice and Behavior,* 37, 239-255.

Gudjonsson, G.H., & Young, S. (2007). The role and scope of forensic clinical psychology in secure unit provisions: A proposed service model for psychological therapies. *The Journal of Forensic Psychiatry & Psychology,* 18, 534-556.

Hall, L., & Long, C.G. (2009). Back to basics: progressive muscle relaxation (PMR) training for women detained in conditions of medium security. *The Journal of Forensic Psychiatry & Psychology,* 20, 481-492.

Hayward, J., McMurran, M., & Sellen, J. (2008). Social problem solving in vulnerable adult prisoners: profile and intervention. *The Journal of Forensic Psychiatry & Psychology,* 19, 243-248.

Heckman, J.P., Hutchins, F.A., Thom, J.C., & Russell, L.A. (2004). Allies: Integrating Women's Alcohol, Drug, Mental Health and Trauma Treatment in a County System. *Alcoholism Treatment Quarterly,* 22, 161-180.

Holtfreter, K., & Wattanaporn, K.A. (2014). The Transition from Prison to Community Initiative An Examination of Gender Responsiveness for Female Offender Reentry. *Criminal Justice and Behavior,* 41, 41-57.

Horsfall, J., Cleary, M., Hunt, G.E., & Walter, G. (2009). Psychosocial treatments for people with co-occurring severe mental illnesses and substance use disorders (dual diagnosis): a review of empirical evidence. *Harvard Review of Psychiatry,* 17, 24-34.

Jaffe, M. (2012). The listener scheme in prisons: Final report on the research findings. Keele: Keele University/ Samaritans.

Johnson, R. (2013). Pervasive interactions: a purposive best evidence review with methodological observations on the impact of housing circumstances and housing interventions on adult mental health and well-being. *Housing, Care and Support,* 16, 32-49.

Jordan, M. (2012). Patients’/prisoners’ perspectives regarding the National Health Service mental healthcare provided in one Her Majesty’s Prison Service establishment. *The Journal of Forensic Psychiatry & Psychology,* 23, 722-739.

Karoll, B.R. (2010). Applying social work approaches, harm reduction, and practice wisdom to better serve those with alcohol and drug use disorders. *Journal of Social Work,* 10, 263-281.

Kavanagh, L., & Borrill, J. (2013). Exploring the experiences of ex-offender mentors. *Probation Journal,* 60, 400-414.

Keinan, G., & Malach-Pines, A. (2007). Stress and Burnout Among Prison Personnel: Sources, Outcomes, and Intervention Strategies. *Criminal Justice and Behavior,* 34, 380-398.

Khalifa, N., Saleem, Y., & Stankard, P. (2008). The use of telepsychiatry within forensic practice: A literature review on the use of videolink. *The Journal of Forensic Psychiatry & Psychology,* 19, 2-13.

Kim, M.M., Calloway, M.O., & Selz-Campbell, L. (2004). A two-level community intervention model for homeless mothers with mental health or substance abuse disorders. *Journal of Community Practice,* 12, 107-122.

Kinderman, P. (2009). Understanding and Addressing Psychological and Social Problems: the Mediating Psychological Processes Model. *International Journal of Social Psychiatry,* 55, 464-470.

Kipping, R.R., Scott, P., & Gray, C. (2011). Health needs assessment in a male prison in England. *Public Health,* 125, 229-233.

Kirchner, J.E., Cody, M., Thrush, C.R., Sullivan, G., & Rapp, C.G. (2004). Identifying factors critical to implementation of integrated mental health services in rural VA community-based outpatient clinics. *Journal of Behavioral Health Services & Research,* 31, 13-25.

Klimidis, S., Hsiao, F.-H., & Minas, I.H. (2007). Chinese-Australians' knowledge of depression and schizophrenia in the context of their under-utilization of mental health care: an analysis of labelling. *International Journal of Social Psychiatry,* 53, 464-479.

Lad, S. (2013a). A case report of treatment of offence related PTSD. *The Journal of Forensic Psychiatry & Psychology,* 24, 788-794.

Lad, S. (2013b). Evaluating the most appropriate approach to psychological therapy provision in prison In N.H.N.F. Trust (Ed.), SBK Healthcare conference. Birmingham: SBK Healthcare.

Lambert, E.G., Altheimer, I., & Hogan, N.L. (2010). Exploring the Relationship Between Social Support and Job Burnout Among Correctional Staff. *Criminal Justice and Behavior,* 37, 1217-1236.

Lambert, E.G., Altheimer, I., Hogan, N.L., & Barton-Bellessa, S.M. (2011). Correlates of Correctional Orientation in a Treatment-Oriented Prison: A Partial Test of Person—Environment Fit Theory. *Criminal Justice and Behavior,* 38, 453-470.

Lambert, E.G., Hogan, N.L., Barton-Bellessa, S.M., & Jiang, S. (2012). Examining the Relationship Between Supervisor and Management Trust and Job Burnout Among Correctional Staff. *Criminal Justice and Behavior,* 39, 938-957.

Le Boutillier, C., Leamy, M., Bird, V.J., Davidson, L., Williams, J., & Slade, M. (2011). What does recovery mean in practice? A qualitative analysis of international recovery-oriented practice guidance. *Psychiatric Services,* 62, 1470-1476.

LeBel, T.P., Burnett, R., Maruna, S., & Bushway, S. (2008). The 'chicken and egg' of subjective and social factors in desistance from crime. *European Journal of Criminology,* 5, 131-159.

Lindqvist, P. (2007). Mental disorder, substance misuse and violent behaviour: the Swedish experience of caring for the triply troubled. *Criminal Behaviour and Mental Health,* 17, 242-249.

Markoff, L.S., Finkelstein, N., Kammerer, N., Kreiner, P., & Prost, C.A. (2005). Relational systems change: implementing a model of change in integrating services for women with substance abuse and mental health disorders and histories of trauma. *Journal of Behavioral Health Services & Research,* 32, 227-240.

Marks, L., Gray, A., & Pearce, S. (2006). General practice in prisons in England: Views from the field. *International Journal of Prisoner Health,* 2, 49-62.

Marle, H.J.C.V. (2007). Mental health care in prison: How to manage our care. *International Journal of Prisoner Health,* 3, 115-123.

Marlow, E., Nyamathi, A., Grajeda, W.T., Bailey, N., Weber, A., & Younger, J. (2012). Nonviolent Communication Training and Empathy in Male Parolees. *Journal of Correctional Health Care,* 18, 8-19.

Martin, J. (2006). All together now (housing and health care). Inside Housing pp. pp16-17 2006).

Martin, R.E., Murphy, K., Hanson, D., Hemingway, C., Ramsden, V., Buxton, J., et al. (2009). The development of participatory health research among incarcerated women in a Canadian prison. *International Journal of Prisoner Health,* 5, 95-107.

Martinez, D.J. (2010). Role accumulation theory and prisoner reintegration: The pursuit of transformative social roles. *Probation Journal,* 57, 139-151.

Masterson, S., & Owen, S. (2006). Mental health service user's social and individual empowerment: Using theories of power to elucidate far-reaching strategies. *Journal of Mental Health,* 15, 19-34.

McMurran, M. (2007). What works in substance misuse treatments for offenders? *Criminal Behaviour and Mental Health,* 17, 225-233.

McMurran, M., & Ward, T. (2010). Treatment readiness, treatment engagement and behaviour change. *Criminal Behaviour and Mental Health,* 20, 75-85.

McNeill, F. (2006). A desistance paradigm for offender management. *Criminology and Criminal Justice,* 6, 39-62.

McNeill, F., & Weaver, B. (2010). Changing lives? Desistance research and offender management. Glasgow: Scottish Centre for Crime & Justice Research.

Mezey, G.C., Kavuma, M., Turton, P., Demetriou, A., & Wright, C. (2010). Perceptions, experiences and meanings of recovery in forensic psychiatric patients. *The Journal of Forensic Psychiatry & Psychology,* 21, 683-696.

Milaney, K. (2012). The 6 dimensions of promising practice for case managed supports to end homelessness: part 2: the 6 dimensions of quality. *Professional Case Management,* 17, 4-12; quiz 13-14.

Miles, O.B. (2009). Audit of handover documentation during patient transfers between learning disability psychiatry community teams. *The Psychiatrist,* 33, 341-343.

Minoudis, P., Shaw, J., & Craissati, J. (2012). The London Pathways Project: Evaluating the effectiveness of a consultation model for personality disordered offenders. *Criminal Behaviour and Mental Health,* 22, 218-232.

Murray, M., Culver, T., Farmer, E., Jackson, L., & Rixon, B. (2013). From Theory to Practice: One Agency’s Experience with Implementing an Evidence-Based Model. *Journal of Child and Family Studies*, 1-10.

Nebelkopf, E., & Penagos, M. (2005). Holistic Native network: integrated HIV/AIDS, substance abuse, and mental health services for Native Americans in San Francisco. *Journal of Psychoactive Drugs,* 37, 257-264.

Nedderman, A.B., Underwood, L.A., & Hardy, V.L. (2010). Spirituality Group With Female Prisoners: Impacting Hope. *Journal of Correctional Health Care,* 16, 117-132.

Nee, C., & Farman, S. (2007). Dialectical behaviour therapy as a treatment for borderline personality disorder in prisons: Three illustrative case studies. *The Journal of Forensic Psychiatry & Psychology,* 18, 160-180.

Opie, A. (2012). Vision narratives, hope and transitions in the Antipodes: Early engagement with possibilities of desistance. *Probation Journal,* 59, 203-218.

Parsonage, M. (2009). *Diversion: A better way for criminal justice and mental health*: Sainsbury Centre for Mental Health.

Persons, R.W. (2009). Art Therapy With Serious Juvenile Offenders: A Phenomenological Analysis. *International Journal of Offender Therapy and Comparative Criminology,* 53, 433-453.

Quinn, C., Byng, R., Shenton, D., & Porter, I. (2012). COCOA: Care for Offenders, Continuity of Access. Unpublished Organisational Case Study Report.

Reed, P., Alenazi, Y., & Potterton, F. (2009). Effect of time in prison on prisoners’ use of coping strategies. *International Journal of Prisoner Health,* 5, 16-24.

Reilly, S., Hughes, J., & Challis, D. (2010). Case management for long-term conditions: implementation and processes. *Ageing & Society,* 30, 125-155.

Reimer, G.R. (2007). Transforming Correctional Health Care Through Advanced Correctional Nursing Education. *Journal of Correctional Health Care,* 13, 163-169.

Repper, J. (2013). Peer Support Workers: Theory and Practice. London: Mental Health Network NHS Confederation and Centre for Mental Health.

Ricketts, T., Brooker, C., & Dent-Brown, K. (2007). Mental health in-reach teams in English prisons: Aims, processes and impacts. *International Journal of Prisoner Health,* 3, 234-247.

Robillard, A.G., L. Braithwaite, R., Gallito-Zaparaniuk, P., & Kennedy, S. (2011). Challenges and Strategies of Frontline Staff Providing HIV Services for Inmates and Releasees. *Journal of Correctional Health Care,* 17, 344-360.

Rosenheck, R., Morrissey, J., Lam, J., Calloway, M., Stolar, M., Johnsen, M., et al. (2001). Service delivery and community: social capital, service systems integration, and outcomes among homeless persons with severe mental illness. *Health Services Research,* 36, 691-710.

Rutherford, M., Samele, C., & Duggan, S. (2008). Building momentum: how the Sainsbury Centre for Mental Health's Prisons and Criminal Justice Programme is contributing to the pressure for change. *Criminal Behaviour and Mental Health,* 18, 261-267.

Saitz, R., Larson, M.J., LaBelle, C., Richardson, J., & Samet, J.H. (2008). The case for chronic disease management for addiction. *Journal of Addiction Medicine,* 2, 55-65.

Salman, S. (2011). Another way: transforming peoples' lives through good practice in adult social care. Voluntary Organisations Disability Group.

Schön, U.-K., Denhov, A., & Topor, A. (2009). Social relationships as a decisive factor in recovering from severe mental illness. *The International journal of social psychiatry,* 55, 336.

Scott, A., & Doughty, C. (2012). “Confronted with paperwork”: Information and documentation in peer support. *Journal of Mental Health,* 21, 154-164.

Shelter. (2007). Good practice briefing: Service without substance: addressing the gaps in service provision for street homeless people with a dual diagnosis. London: Shelter.

Short, V., Cooper, J., Shaw, J., Kenning, C., Abel, K., & Chew-Graham, C. (2009). Custody vs care: attitudes of prison staff to self-harm in women prisoners—a qualitative study. *The Journal of Forensic Psychiatry & Psychology,* 20, 408-426.

Smith, & Mogro-Wilson, C. (2007). Multi-level influences on the practice of inter-agency collaboration in child welfare and substance abuse treatment. *Children and Youth Services Review,* 29, 545-556.

Smith, F.M., & Marshall, L.A. (2007). Barriers to effective drug addiction treatment for women involved in street-level prostitution: a qualitative investigation. *Criminal Behaviour and Mental Health,* 17, 163-170.

Strauss, D. (2006). A community-based organization's integration of HIV and substance abuse treatment services for ex-offenders. *Social Work in Health Care,* 42, 61-76.

Sumter, M.T., Monk-Turner, E., & Turner, C. (2009). The Benefits of Meditation Practice in the Correctional Setting. *Journal of Correctional Health Care,* 15, 47-57.

Swenson, D.X., Waseleski, D., & Hartl, R. (2008). Shift Work and Correctional Officers: Effects and Strategies for Adjustment. *Journal of Correctional Health Care,* 14, 299-310.

Thompson, M. (2000). Five giant leaps toward integrating health care delivery and ways to drive organizations to leap or get out of the way. *Journal of Ambulatory Care Management,* 23, 1-18.

Tompkins, C.N.E., Neale, J., Sheard, L., & Wright, N.M.J. (2007). Experiences of prison among injecting drug users in England: A qualitative study. *International Journal of Prisoner Health,* 3, 189-203.

Ungar, M., Liebenberg, L., & Ikeda, J. (2012). Young people with complex needs: Designing coordinated interventions to promote resilience across child welfare, juvenile corrections, mental health and education services. *British Journal of Social Work*.

Unruh, D., Gau, J., & Waintrup, M. (2009). An Exploration of Factors Reducing Recidivism Rates of Formerly Incarcerated Youth with Disabilities Participating in a Re-Entry Intervention. *Journal of Child and Family Studies,* 18, 284-293.

Urada, D., Schaper, E., Alvarez, L., Reilly, C., Dawar, M., Field, R., et al. (2012). Perceptions of mental health and substance use disorder services integration among the workforce in primary care settings. *Journal of Psychoactive Drugs,* 44, 292-298.

Van Harreveld, F., Van der Pligt, J., Claassen, L., & Van Dijk, W.W. (2007). Inmate Emotion Coping and Psychological and Physical Well-Being: The Use of Crying Over Spilled Milk. *Criminal Justice and Behavior,* 34, 697-708.

Vanderplasschen, W., Rapp, R.C., Wolf, J.R., & Broekaert, E. (2004). The development and implementation of case management for substance use disorders in North America and Europe. *Psychiatric Services,* 55, 913-922.

Vaughan, B. (2007). The internal narrative of desistance. *British Journal of Criminology,* 47, 390-404.

Verhaeghe, M., Bracke, P., & Bruynooghe, K. (2008). Stigmatization and self-esteem of persons in recovery from mental illness: The role of peer support. *International Journal of Social Psychiatry,* 54, 206-218.

Wallace, B.C., Conner, L.C., & Dass-Brailsford, P. (2011). Integrated Trauma Treatment in Correctional Health Care and Community-Based Treatment Upon Reentry. *Journal of Correctional Health Care,* 17, 329-343.

Walsh, E., & Freshwater, D. (2009). Developing the Mental Health Awareness of Prison Staff in England and Wales. *Journal of Correctional Health Care,* 15, 302-309.

Ward, T. (2002a). Good lives and the rehabilitation of offenders: Promises and problems. *Aggression and Violent Behavior,* 7, 513-528.

Ward, T. (2002b). The management of risk and the design of good lives. *Australian Psychologist,* 37, 172-179.

Ward, T., & Brown, M. (2004). The good lives model and conceptual issues in offender rehabilitation. *Psychology, Crime and Law,* 10, 243-257.

Ward, T., Mann, R.E., & Gannon, T.A. (2007). The good lives model of offender rehabilitation: Clinical implications. *Aggression and Violent Behavior,* 12, 87-107.

Weinstein, L.C., Lanoue, M.D., Plumb, J.D., King, H., Stein, B., & Tsemberis, S. (2013). A primary care-public health partnership addressing homelessness, serious mental illness, and health disparities. *Journal of the American Board of Family Medicine: JABFM,* 26, 279-287.

Whittle, N., Macdonald, W., & Bailey, S. (2012). A Study of Young Offenders’ Perceptions of Health and Health Care Services in Custody and in the Community. *Journal of Correctional Health Care*.

Windell, D., & Norman, R.M. (2012). A qualitative analysis of influences on recovery following a first episode of psychosis. *International Journal of Social Psychiatry*.

Winter, S.J. (2008). Improving the Quality of Health Care Delivery in a Corrections Setting. *Journal of Correctional Health Care,* 14, 168-182.

Wolff, N., & Draine, J. (2004). Dynamics of Social Capital of Prisoners and Community Reentry: Ties That Bind? *Journal of Correctional Health Care,* 10, 457-490.
