# Supplementary material for: Using realist review to inform intervention development: methodological illustration and conceptual platform for collaborative care in offender mental health
Source: Implement Sci. 2015 Sep 28;10:134. doi: 10.1186/s13012-015-0321-2 (PMC4584430; doi:10.1186/s13012-015-0321-2)
Supplement: Additional file 5: — Consolidated explanatory accounts. [file 13012_2015_321_MOESM5_ESM.docx]

**Supplementary File 5: Consolidated Explanatory Account Table**

| **#** | **Consolidated explanatory account** |
| --- | --- |
|  | ***Practitioners - Organisational context*** |
| 1 | *If* a broad spectrum of already existing health and non-health services are mobilised by the intervention towards a range of idiographic goals  *and* commissioning enables this  *and* they share a clear and common purpose that relates to each of their individual missions  *and* collaboratively develop and review multi-agency protocols  *Then* person-centred service provision will be increased |
| 39 | *If* there is trauma screening and assessment to identify those most vulnerable to trauma in the prison setting as well as practitioner training and effective monitoring of offenders to prevent and respond to incidents of trauma (because there is a high risk or trauma in prison in addition to a likely prior history of trauma in an offenders past)  *And* practitioners are skilled in dealing with people who have experienced abuse/trauma in the past (IAPT +)  *Then* risk of re-traumatisation in prison and the community is reduced  *And* offender wellbeing is improved |
| 43 | *If* there is congruence between staff’s and the organisation’s (prison’s) values  *then* staff satisfaction in the workplace is improved  *and* staff implement care/custody programmes in the way intended by the organisation |
| 50 | *If* practitioners in prisons are trained on issues of comorbidity, dual diagnosis, PD, self-harm, dealing with literacy problems and learning disabilities, risk and safety and have above average levels of supervision  *Then* their work with offender populations will be better supported and more effective |
| 52 | *If* case management includes extensive training and supervision to foster collaboration and pre-contracting of services to ascertain their availability  *And* there are formal agreements and protocols concerning the tasks, responsibilities, and authorities of case managers and other service providers involved; the use of common assessment and planning tools; and exchange and management of client information  *Then* cooperation and coordination between services can be enhanced |
| 53 | *If* health and social care professionals are co-located  *Then* the extent of partnership working is increased and integrated care is improved |
| 56 | *If* cross-cutting targets *across* organisations can be agreed and supported by pooled budgets  *Then* services could be more inclusive of street homeless people with a dual diagnosis |
| 58 | *If* the lead case manager holds multidisciplinary team members accountable  *Then* integrated working is facilitated |
| 61 | *If* there is ‘buy-in’ to joint working at both a strategic and operational level  *And* there is ongoing two-way communication between the levels  *Then* collaborative, cross-sector working is enabled |
|  |  |
|  | ***Practitioners - Social/cultural context*** |
| 25 | *If* there are clear roles for professionals working in multi-disciplinary teams and a shared understanding for each offender who holds clinical responsibility or case management responsibility  *And* ongoing clear communication of problem areas and solutions  *Then* liaison between professionals will be supported by removing uncertainty and/or power struggles between teams/professionals  *Then* support for offender wellbeing will be supported by reducing diffusion of responsibility |
| 2 | *If* practitioners know of services, why to refer offenders to them, who to talk to about referral, and how to refer  *and* perceive that referrals will be welcomed  *then* the number of appropriate referrals will increase |
|  |  |
|  | ***Interactions between practitioners and practitioners*** |
| 2 | *If* practitioners know of services, why to refer offenders to them, who to talk to about referral, and how to refer  *and* perceive that referrals will be welcomed  *then* the number of appropriate referrals will increase |
| 17 | *If* specialist mental health practitioners provide positive feedback to non-specialist practitioners regarding their work with offenders with CMHPs  *Then* this will support and maintain liaison between MH and non-MH professionals |
| 20 | *If* there is someone in the prison full-time that in-reach teams can liaise with  *Then* this facilitates identifying offender needs asap and starting to make contacts with other services |
| 22 | *If* there are opportunities to build individual relationships with people in other services in and out of prison (rather than ‘paper liaison’)  *And* trust is built up between services and the practitioners in them  *And* staff are trained in inter-agency collaborative practice or helped to see potential benefits of collaborative practice  *Then* liaison between practitioners will be facilitated (and perceived as easier) |
| 24 | *If* there is a shared language between professionals across services in and out of prison (e.g. through same training and open lines of communication)  *Then* liaison between teams will be facilitated |
| 32 | *If* offenders see health services in prison as one service but practitioners see them as separate services with separate provision  *And / Or* there is a failure to communicate with other agencies so as to coordinate care  *Then* there will be repeated assessment or disjointed care provision  *And Then* offenders will be more likely to become disengaged with all health services in prison |
| 35 | *If* practitioner acts as an advocate for the offender, practicing assertive outreach and getting the offender to services they need  *then* offender service engagement will increase |
| 37 | *If* all collaborators within a network feel the net worth of their involvement in the network (i.e. practitioners, institutions, agencies) through appropriate incentives  *Then* collaborative working is enabled |
| 52 | *If* case management includes extensive training and supervision to foster collaboration and pre-contracting of services to ascertain their availability  *And* there are formal agreements and protocols concerning the tasks, responsibilities, and authorities of case managers and other service providers involved; the use of common assessment and planning tools; and exchange and management of client information  *Then* cooperation and coordination between services can be enhanced |
|  |  |
|  | ***Practitioners - Engagement*** |
| 5 | *If* practitioners are motivated both intrinsically (e.g. pride, values) and extrinsically (social or financial rewards)  *and* practitioners’ well-being at work is fostered  *and* organisational culture enables practitioners to pursue personal and professional goals  *and* practitioners feel supported by, and trust, colleagues and supervisors  *then*  practitioners’ engagement with an offender wellbeing ethos is increased  *and* staff retention and service continuity is improved |
| 6 | *If* practitioners do not share external perceptions of the standard of services delivered  *then* practitioners’ engagement with changes in practice is decreased |
| 7 | *If* proposed changes in practice challenge traditional ways of working (e.g. role, status, autonomy, relationship with service users)  *then* practitioners’ engagement with changes in practice is decreased |
| 8 | *If* practitioners’ experiential knowledge is incorporated into proposed service changes  *and/or* practitioners identify with the experience and knowledge of those endeavouring to implement change  *then* practitioners’ engagement with an offender wellbeing ethos is increased |
|  |  |
|  | ***Practitioners - Understanding and skills*** |
| 18 | *If* health practitioners are unfamiliar with the prison system and how to work within it  *Then* opportunities to initially engage offenders may be put at risk |
| 19 | *If* there are fundamental differences in opinion about custody and treatment models that exist between CJ and healthcare providers  *or* differences in knowledge and understanding about mental health  *Then* provision of a prison environment that promotes wellbeing will be limited |
| 28 | *If* practitioners can develop the skills to reflect on their own practice  *and* work towards practising empathically  *then* offenders’ well-being will increase |
| 36 | *If* offenders’ and practitioners’ ability to look at another person’s perspective is increased through the use of mentalisation-based skills training  *then* practitioner creativity will be supported  *and* offender relationships will be supported |
| 41 | *If* practitioners assume that offenders’ families can offer support (social, emotional, values, resources) in a similar way to ‘conventional middle-class families’  *Then* the potential for enabling offenders to mobilise their social capital is lessened |
| 50 | *If* practitioners in prisons are trained on issues of comorbidity, dual diagnosis, PD, self-harm, dealing with literacy problems and learning disabilities, risk and safety and have above average levels of supervision  *Then* their work with offender populations will be better supported and more effective |
|  |  |
|  | ***Interactions between practitioners and offenders*** |
| 10 | *If* care making up the intervention for the individual is based around a choice of what the individual wants and a range of evidence-based treatments are available  *and* practical and emotional support are offered in tandem  *Then* individuals are motivated and engaged in their care |
| 12 | *If* idiographic goals, selected as important to the offender, are the focus of care (rather than just the ‘known’ average needs of care group)  *Then* motivation/energy/agency of offenders taking part in intervention is mobilised. |
| 13 | *If* a ‘transition specialist’ provides interactions that stress self-determination, social skill training, and developing a set of services based on the needs and strengths of the youth offender and generally supports youth engagement in services  *and* a mentoring relationship is established  *Then* these interactions may be more powerful than offender being only engaged in education and employment and will support youth offender engagement in services |
| 14 | *If* offenders’ families’ wellbeing is supported  *and* there is ongoing information exchange about family wellbeing  *then* offenders’ own cognitive and emotional resources will be freed up for them to engage with their own wellbeing |
| 27 | *If* offenders have the opportunity to explore with a practitioner their personal narrative in a non-judgmental and non-stigmatising environment  *and* support is provided for building and/or re-modelling an identity that is coherent with but does not discount past identities  *and* temporary support is provided as a person with mental health issues leaving prison re-builds their community living skills and to develop persisting network of community ties  *and* this support instils hope (at minimum an ‘adequate sense of hope’)  *then* resettlement and rehabilitation is promoted. |
| 29 | *If* practitioners can ‘repair’ relationships with offenders when offenders feel that this relationship has ‘gone wrong’  *then* progress towards resettlement and rehabilitation can be maintained |
| 30 | *If* the positive (but ultimately short-term) practitioner relationship provides support for the repair or creation of an/other primary relationships in an offender’s life  *and if* emotional support/befriending is provided by a volunteer (rather than only by a professional) who continues to be involved with the offender’s life on release  *then* progress towards resettlement and rehabilitation can be maintained beyond practitioner involvement |
| 33 | *If* all contacts between an offender and any practitioners in prison and community are conceived of as potentially ‘therapeutic’ by the practitioners  *then* offender engagement with services is supported |
| 39 | *If* there is trauma screening and assessment to identify those most vulnerable to trauma in the prison setting as well as practitioner training and effective monitoring of offenders to prevent and respond to incidents of trauma (because there is a high risk or trauma in prison in addition to a likely prior history of trauma in an offenders past)  *And* practitioners are skilled in dealing with people who have experienced abuse/trauma in the past (IAPT +)  *Then* risk of re-traumatisation in prison and the community is reduced  *And* offender wellbeing is improved |
| 40 | *If* imprisonment weakens offenders’ connections with the community  *Then* offenders identify more with prison culture  *Then* individuals’ social capital, confidence in, and ability to, live independently (non-institutionally) is significantly weakened  *Then* this can militate against many of the approaches used in psychological therapies, such as being aware of and open about emotions |
| 42 | *If* the recovery approach to recovering from mental illness is adapted to forensic populations such that it provides offenders with realistic expectations about what lies ahead, rather than false hope; and takes into account the importance of self-esteem and stigma in this population  *And* takes in to account the offender and their family/communities perceptions of their offending behaviour as a barrier to recovery  *Then* this approach can better support recovery from mental illness in forensic populations |
| 44 | *If* offenders’ relationships (and the responsibilities that these relationships entail) can be resumed or initiated at the time of release  *Then* a positive upward spiral of social integration and mutual obligation is enabled |
| 45 | *If* offenders with mental health issues (depression, PTSD, conduct disorder) are engaged using art therapy  *Then* they have a positive avenue for self-expression across a range of psychological needs (identity, security, adventure/fun, parental relationships, affiliation and affection, erotic and sexual needs, experience of depression, expression of childhood trauma and other serious psychological problems, and spiritual or religious needs)  *and* destructive behaviour is reduced  *and* mental wellbeing is improved |
| 47 | *If* individuals display lower levels of distress  *Then* they may benefit from more prescriptive, group-based therapies  *But* if they have higher levels of distress  *Then* they may be better suited to individually tailored and implemented treatment |
| 48 | *If* practitioners support vulnerable people to exercise agency and be involved in the negotiations for service provision and discussing their plan of care­­  *Then* the more likely it is that treatment is going to be effective |
| 60 | *If* treatment plans foster coping and decision-making capabilities, self-advocacy, self-care, and sustainable support systems that will continue after professional services have ended  *Then* a holistic service that improves health outcomes is attained |
| 62 | *If* short-term ‘recovery’ outcomes are prioritised over longer-term ‘maintenance and prevention’ outcomes  *Then* service-user centred goals and integrated working are inhibited |
| 63 | *If* offenders’ efforts to desist from crime are recognised and rewarded by practitioners  *Then* reintegration in communities is promoted |
| 64 | *If* work with offenders is conducted on the basis that desistance from crime is a (potentially long) journey  *and* offenders are facilitated to understand their journey in its social and cultural context  *and* offenders’ significant others are engaged as partners on the journey  *and* workers retain sufficient flexibility to increase or decrease their support at different stages of the offenders’ journey  *Then* desistance from crime and community reintegration is facilitated |
| 65 | *If* practitioners’ interactions with offenders are not sensitive to the importance of core aspects of offenders’ identities (gender, ethnicity, religion/spirituality)  *Then* an individualised, strengths-based approach to desistance is hindered |
| 66 | *If* people with dual diagnosis of mental health issues and drug misuse have family support from people who remain involved  *Then* this may enhance both group and individual approaches, and have a significant impact on clinical outcomes and recovery |
| 67 | *If* initial contact with a person with drug dependency includes listening attentively and reflecting back their story  *And* it includes offering choices for meeting times that are outside 9 – 5 hours (flexibility in availability)  *Then* initial engagement in intervention will be improved |
| 68 | The assessment process provides an early and ongoing opportunity for the client/social worker partnership to name and rename the problem, shifting perspectives from deficits to strengths and providing the client opportunities to have voice in shaping the method for problem remediation. |
| 70 | *If* prisoners have the opportunity to discuss their anxieties with a peer who is skilled in listening  *Then* a negative spiral of anxiety and depression can be arrested  *And* a firmer foundation laid for wellbeing |
| 72 | *If* a person can be enabled to become aware of and think in a non-judgemental way about their own thoughts, emotions and actions  *And* a balance can be struck between intellectual analysis and emotional involvement  *Then* a person’s *relationship* with their thoughts and emotions can be improved (even if the thoughts and emotions remain unchanged)  *And* the foundations are laid for a person to decide how they may wish to act differently in the future |
| 73 | *If* a safe and sensitive interpersonal environment can be created  *Then* there is space for a person to focus on reflection and ‘mentalisation’ whilst regulating their affect |
| 74 | Mentalisation (understanding why oneself and others do what they do) ‘works’ by:   - a. stimulating a joint consideration of underlying processes (rather than the therapist ‘knowing’ and the person ‘receiving’ this knowledge) - b. acknowledging and exploring the different components of thought processes (rather than the therapist showing a person their ‘inaccuracy’)   c. helping a person to attend to his or her own feelings (rather than identifying and naming these) |
| 75 | *If* an offender perceives that there is an absence of care in the general prison environment  *And* the offender perceives that in the prison environment they are not in a position of power in relation to other people  *And* the relationship with a practitioner is perceived as caring and equalising the power differential  *Then* the relationship is additionally therapeutic |
|  |  |
|  | ***Offenders - Organisational context*** |
| 16 | *If* the prison environment offers choice in, and access to, services  *and* rules are explained and consistently applied  *and* interactions within it are characterised by tolerance  *then* offenders’ motivation to engage with services is increased |
| 49 | *Prisoners say that they need something to do during the day: meaningful activity, including work and exercise.* |
| 57 | *If* a multidisciplinary team is diverse (in terms of gender, culture, etc.)  *Then* clients will have greater opportunity to develop relationships with people who they have a particular connection with |
|  |  |
|  | ***Offenders - Engagement*** |
| 15 | *If* basic needs and wants for shelter and food are met on release from prison  *then* offenders are better able to engage with a wider range of services |
| 34 | *If* the very first contact between services and offenders emphasises the positives without flagging up any negatives of service engagement  *and* if first contact gives the offender something they need/want  *then* offenders’ first perception of services is positive and engagement will be improved |
| 35 | *If* practitioner acts as an advocate for the offender, practicing assertive outreach and getting the offender to services they need  *then* offender service engagement will increase |
| 67 | *If* initial contact with a person with drug dependency includes listening attentively and reflecting back their story  *And* it includes offering choices for meeting times that are outside 9 – 5 hours (flexibility in availability)  *Then* initial engagement in intervention will be improved |
|  |  |
|  | ***Offenders - Perceptions, understanding and skills*** |
| 3 | *If* offenders believe that discussing mental health may lead to treatment that impacts negatively on the length of their sentence  *then* offenders’ mental wellbeing will remain unaddressed |
| 4 | *If* offenders believe that they can trust practitioners and perceive that they will be treated empathically and fairly  *and* believe that there will be continuity of service provision  *then* offenders will feel able to approach and engage with services |
| 26 | *If* offenders’ coping and communication capabilities and social skills can be improved  *then* their ability to empathise will increase  *and* their ability to form or re-connect with a supportive social environment (which offers opportunities for pursuing a non-offender identity) is increased  *and* resettlement and rehabilitation is promoted |
| 31 | *If* offenders’ concerns about past experiences of services  *and* their expectations about service provision are discussed  *then* engagement can be started and maintained even where service provision is imperfect |
| 32 | *If* offenders see health services in prison as one service but practitioners see them as separate services with separate provision  *And / Or* there is a failure to communicate with other agencies so as to coordinate care  *Then* there will be repeated assessment or disjointed care provision  *And Then* offenders will be more likely to become disengaged with all health services in prison |
| 46 | *If* an approach is used with offenders with a PD that focuses on developing core mindfulness skills, with an emphasis on awareness of the moment and identification of mood states  *Then* this will reduce dissociation and reckless behaviours  Then offenders will be more capable of learning other types of skills because of this which can also increase sense of self and regulation of emotion and thus quality of life |
| 71 | *If* people in recovery from mental ill-health share their own stories and model empowerment  *Then* this can help others to improve their self-management skills and ability to take personal responsibility |
|  |  |
|  | ***NOT ACCOUNTED FOR IN CONCEPT MAP*** |
| 9 | *If* practitioners utilise their strengths and creativity  *Then* intervention effectiveness and resultant wellbeing if offenders will be improved |
| 11 | *If* care generated by the intervention is designed to utilise the strengths and creativity of an individual (rather than them being a passive object of intervention)  *Then* wellbeing is improved |
| 21 | *If* in-reach MH teams work with patients prior to release  *Then* continuity of care is supported |
| 23 | *If* there is a formalised liaison process (such as stepped care)  *Then* this supports an integrated approach to case management |
| 38 | *If* the public had a better awareness and understanding of prisons’ role in society, including prisons’ commitment to mental health and illness  *Then* this would support prisoners’ mental health post-release (i.e. through better reintegration into communities etc.) |
| 51 | *If* interventions are tailored to an individual’s readiness to change stages (pre-contemplation, contemplation, preparation, action, maintenance) with an emphasis on developing treatment readiness prior to treatment referral (i.e. use motivational interviewing to move forwards from pre-contemplation or contemplation)  *And* there is continual assessment and reassessment of their motivation to change  *Then* treatment of dually diagnosed mental health issues and drug misuse issues will be more effective |
| 54 | *If* care budgets are ‘individualised’ (i.e. decisions about how to spend are made by recipients)  *Then* the matrix of services provided will match individuals’ needs and wants more closely |
| 55 | *If* trust, co-operation and co-ordination between practitioners can be fostered by systems integration and social capital of practitioners  *Then* the performance of service delivery agencies is improved and user health outcomes are improved |
| 59 | *If* prison terminology changes from ‘health needs assessment’ to ‘health’, ‘wellbeing’, and ‘dignity’  *Then* this will enable the partnership board to focus on the prison as a whole system, and to identify aspects of the environment and culture which may enhance or detract from the promotion of health in its widest sense |
| 69 | *If* ex-offenders act as mentors to other offenders  *Then* they feel empowered, are enabled to continue on a path of desistance by having an opportunity to give back to society, are encouraged in their personal and professional growth and increasing self-esteem, develop an array of interpersonal skills, benefit from building trusting and open relationships with their mentees, and are kept grounded, being reminded in their mentoring activities of paths they never want to walk down again, such as homelessness, addiction, and criminal behaviour, and their own positive changes are reinforced. |
