# Supplementary material for: Using realist review to inform intervention development: methodological illustration and conceptual platform for collaborative care in offender mental health
Source: Implement Sci. 2015 Sep 28;10:134. doi: 10.1186/s13012-015-0321-2 (PMC4584430; doi:10.1186/s13012-015-0321-2)
Supplement: Additional file 6: — Sources of explanatory accounts. [file 13012_2015_321_MOESM6_ESM.docx]

**Supplementary File 6: Sources of Explanatory Accounts (EA)**

| **Consolidated EA no.** | **EA no.** | **Citation/s (Record #)** |
| --- | --- | --- |
| 1 | 17 | Study Group |
|  | 191 | Study Group 25/11/13 |
|  | 265 | Ales (2011) p. S18 (#621) |
|  | 276 | Brown (2008) p.93 (#674) |
|  | 281 | Voluntary Organisations Disability Group (2011) p.16 (#711) |
|  | 284 | Weinstein et al. (2013) p.285 (#785) |
|  | 287 | Shelter (2007) p.4 (#794) |
| 2 | 21 | Quinn et al. (2012) p.13 (#329) |
|  | 22 | Quinn et al. (2012) p.14 (#329) |
|  | 27 | Quinn et al. (2012) p.26 (#329) |
|  | 86 | Walsh (2009) p.304/5/7 (#168) |
|  | 88 | Walsh (2009) p.307 (#168) |
|  | 140 | Ricketts et al. (2007) p.241 (#34) |
|  | 144 | Ricketts et al. (2007) p.242 (#34) |
|  | 145 | Ricketts et al. (2007) p.242 (#34) |
|  | 146 | Ricketts et al. (2007) p.242 (#34) |
|  | 183 | Goldstein et al. (2006) p. 189/195/196 (#136) |
| 3 | 14 | October 2013 Meeting with Peer Researcher |
| 4 | 31 | Feron et al. (2008), p.151 (#27) |
|  | 35 | October 2013 Peer Research Group |
|  | 42 | Emlyn-Jones (2007), p.240 (#79) |
|  | 49 | Schon et al. (2009), p.341 (#122) |
|  | 54 | Collins & Barker (2009), p.379 (#123) |
|  | 105 | Robillard (2011) p.353 (#177) |
|  | 106 | PRG meeting Oct 2013 |
|  | 127 | van Marle et al. (2007) p.120 (#32) |
|  | 128 | van Marle et al. (2007) p.120 (#32) |
|  | 162 | Unruh et al. (2009) p.291 (#105) |
|  | 177 | Fortune et al. (2010) p. 190 (#129) |
|  | 209 | Scott & Doughty (2012) p. 154 (#91) |
|  | 257 | Revolving Doors (2011) p. 9 (#558) |
|  | 305 | McNeill & Weaver (2010) p.7 (#824) |
|  | 342 | Ward (2002), p.173-4 (#850); Ward (2002) (#851); Ward & Brown (2004) (#852); Ward et al. (2007) (#854) |
| 5 | 60 | Buetow (2013) p.183 (#3) |
|  | 61 | Buetow (2013) p.184 (#3) |
|  | 62 | Buetow (2013) p.184 (#3) |
|  | 63 | Buetow (2013) p.184 (#3) |
|  | 64 | Buetow (2013) p.185 (#3) |
|  | 65 | Buetow (2013) p.185 (#3) |
|  | 66 | Davies et al (2007) p. 129 (#5) |
|  | 94 | Winter (2008) p. 179 (#157) |
|  | 218 | Chafin & Biddle (2013) p. 124 (#185) |
|  | 229 | Lambert et al. (2010) (#479), p.1230/ Lambert et al. (2012) (#481) |
|  | 234 | Brough &Wiliams (2007) p. 566 (#321) |
|  | 274 | Allsop (2009) p. 545 (#635) |
| 6 | 67 | Davies et al (2007) p. 129 (#5) |
|  | 68 | Davies et al (2007) p. 129 (#5) |
| 7 | 69 | Davies et al (2007) p. 129 (#5) |
|  | 70 | Davies et al (2007) p. 130 (#5) |
|  | 92 | Winter (2008) p. 178 (#157) |
|  | 93 | Winter (2008) p. 179 (#157) |
| 8 | 71 | Davies et al (2007) p. 130 (#5) |
|  | 87 | Walsh (2009) p.306/7 (#168) |
| 9 | 94 | Winter (2008) p. 179 (#157) |
|  | 187 | October 2013 presentation slides |
| 10 | 15 | October 2013 presentation slides |
|  | 163 | Unruh et al. (2009) p.291 (#105) |
|  | 190 | Study group 25/11/13 |
|  | 209 | Scott & Doughty (2012) p. 154 (#91) |
|  | 215 | Wallace et al. (2011) p.336 (#176) |
|  | 259 | Repper (2013) p. 8, 9 (#544) |
|  | 296 | Kim et al. (2004) p.110 (#701) |
|  | 321 | Karoll (2010) p. 265 (#589) |
|  | 322 | Karoll (2010) p. 266 (#589) |
| 11 | 16 | October 2013 presentation slides |
|  | 245 | McMurran & Ward (2010) p. 83 (#150) |
|  | 259 | Repper (2013) p. 8, 9 (#544) |
|  | 296 | Kim et al. (2004) p.110 (#701) |
|  | 321 | Karoll (2010) p. 265 (#589) |
|  | 322 | Karoll (2010) p. 266 (#589) |
|  | 326 | Karoll (2010) p. 271 (#589) |
| 12 | 18 | October 2013 presentation slides |
|  | 163 | Unruh et al. (2009) p.291 (#105) |
|  | 209 | Scott & Doughty (2012) p. 154 (#91) |
|  | 245 | McMurran & Ward (2010) p. 83 (#150) |
|  | 259 | Repper (2013) p. 8, 9 (#544) |
|  | 296 | Kim et al. (2004) p.110 (#701) |
|  | 309 | McNeill (2006) p.47 (#832) |
|  | 321 | Karoll (2010) p. 265 (#589) |
|  | 322 | Karoll (2010) p. 266 (#589) |
| 13 | 161 | Unruh et al. (2009) p.290) (#105) |
|  | 162 | Unruh et al. (2009) p.291 (#105) |
|  | 164 | Unruh et al. (2009) p.291 (#105) |
| 14 | 8 | September 2013 Peer Researcher Group |
| 15 | 11 | September 2013 Peer Researcher Group |
|  | 44 | Rutherford et al. (2008), p.265 (#83) |
|  | 102 | Robillard (2011) p.352 (#177) |
| 16 | 128 | van Marle et al. (2007) p.120 (#32) |
|  | 242 | Crawford & Rutter (2007) p. 58 (#332) |
| 17 | 88 | Walsh (2009) p.307 (#168) |
|  | 92 | Winter (2008) p. 178 (#157) |
|  | 144 | Ricketts et al. (2007) p.242 (#34) |
|  | 145 | Ricketts et al. (2007) p.242 (#34) |
| 18 | 91 | Winter (2008) p. 177 (#157) |
|  | 72 | Caulfield and Twort (2012) p. 12 (#61) |
|  | 95 | Winter (2008) p. 180 (#157) |
|  | 142 | Ricketts et al. (2007) p.242 (#34) |
|  | 213 | DeHart et al (2009) p.129 (#162); Short et al. (2013) p.408 (#206) |
| 20 | 140 | Ricketts et al. (2007) p.241 (#34) |
| 21 | 141 | Ricketts et al. (2007) p.242 (#34) |
| 22 | 146 | Ricketts et al. (2007) p.242 (#34) |
|  | 147 | Ricketts et al. (2007) p.242/3 (#34) |
|  | 204 | Chew-Graham et al. (2008) p. 6 (#12) |
|  | 207 | Calnan (2008) p. 97 (#15) |
|  | 228 | Minoudis et al. (2012) p.230 (#230) |
|  | 277 | Smith (2007) p.554 (#677) |
|  | 278 | Urada (2012) p.297 (#687) |
| 23 | 183 | Goldstein et al. (2006) p. 189/195/196 (#136) |
| 24 | 89 | Winter (2008) p. 170/1 (#157) |
|  | 172 | Murray et al. (2013) p. 7 (#110) |
| 25 | 28 | Quinn et al. (2012) p.27 (#329) |
|  | 89 | Winter (2008) p. 170/1 (#157) |
|  | 181 | Fortune et al. (2010) p. 190 (#129) |
|  | 205 | Chew-Graham et al. (2008) p. 6 (#12) |
|  | 264 | Ales (2011) p. S17 (#621) |
|  | 275 | Thomson (2000) p. 6 (#637) |
|  | 289 | Milaney (2012) p.5 (#704) |
|  | 300 | Cameron et al. (2006) (#735) |
| 26 | 188 | Study group 25/11/13 |
|  | 217 | Marlow et al (2012) p. 8 (#178) |
|  | 219 | Hayward, McMurran, & Sellen (2008) p. 243 (#202) |
|  | 221 | Hall & Long (2009) p.481 (#207) |
|  | 222 | Ferguson (2009) p.906 (#210) |
|  | 224 | Wolff & Draine (2004) p.466 (#310) |
|  | 232 | Dhaliwal & Harrower (2009) p. 35 (#274); Jaffe (2012) #316 |
|  | 233 | Van Harreveld (2007) p. 697 (#320) |
|  | 316 | Draine (2007) p. 1577 (#619) |
| 27 | 189 | Study group 25/11/13 |
|  | 214 | Nedderman et al. (2010) p. 171 (#171) |
|  | 236 | Opie (2012) p.210-11 (#348) |
|  | 244 | McMurran & Ward (2010) p. 82 (#150) |
|  | 307 | LeBel et al. (2008) p.154 (#831) |
|  | 308 | LeBel et al. (2008) p.154 (#831) |
|  | 310 | Vaughan (2007) p.401 (#833) |
|  | 316 | Draine (2007) p. 1577 (#619) |
|  | 322 | Karoll (2010) p. 266 (#589) |
|  | 326 | Karoll (2010) p. 271 (#589) |
|  | 327 | Karoll (2010) p. 271 (#589) |
| 28 | 192 | Study group 25/11/13 |
|  | 298 | Kim et al. (2004) p.114 (#701) |
| 29 | 193 | Study group 25/11/13 |
| 30 | 194 | Study group 25/11/13, 66 |
|  | 210 | Tirril Harris 25/11/13 |
|  | 211 | Tirril Harris 25/11/13 |
| 31 | 195 | Study group 25/11/13 |
|  | 197 | Study group 25/11/13 |
| 32 | 198 | Study group 25/11/13 |
|  | 201 | Study group 25/11/13 |
|  | 257 | Revolving Doors (2011) p. 9 (#558) |
| 33 | 199 | Study group 25/11/13 |
|  | 268 | Vanderplasschen (2004) p.919 (#624) |
| 34 | 196 | Study group 25/11/13 |
|  | 200 | Study group 25/11/13 |
| 35 | 202 | Study group 25/11/13 |
| 36 | 203 | Study group 25/11/13 |
| 37 | 206 | Goodwin (2008) p.59 (#14) |
|  | 263 | Ales (2011) p. S17 (#621) |
| 38 | 208 | Fraser (2009) p. 133 (#56) |
| 39 | 216 | Wallace et al. (2011) p.340 (#176); Brooker & Birmingham (2009) p. S3 (#212) |
|  | 255 | Sunil Lad (2013) p. 3 (#565) |
|  | 256 | Sunil Lad (2013) p. 3 (#565 and #818) |
|  | 273 | Amaro (2007) p. 508 & 512 (#630) |
|  | 276 | Brown (2008) p.93 (#674) |
| 40 | 223 | Wolff & Draine (2004) p.461 (#310) |
|  | 252 | Judith Forrest (2013) p. 1, 2 (#564) |
|  | 317  346 | Draine (2007) p. 1579 (#619)  Bateman & Fonagy (2012) (#920) |
| 41 | 225 | Wolff & Draine (2004) p.471 (#310) |
| 42 | 226 | Mezey et al. (2010) p.693 (#225) |
| 43 | 230 | Lambert et al. (2011), p.455 |
| 44 | 235 | Martinez (2010) p.145-7 (#346) |
|  | 319 | Draine (2007) p. 1580 (#619) |
| 45 | 237 | Persons (2009) p.439 (#448) |
| 46 | 238 | Nee & Farman (2007) p.176 (#196) |
|  | 239 | Nee & Farman (2007) p.176 (#196) |
| 47 | 243 | McMurran & Ward (2010) p. 82 (#150) |
| 48 | 246 | Ungar et al. (2012) p. 14 (#521) |
| 49 | 250 | Sainsbury’s Centre (2008) p. 7 (#533); Whittle et al. (2012) (#445) |
|  | 333 | Bensimon (2013) p.1 (#846) |
| 50 | 254 | Judith Forrest (2013) p. 1, 2 (#564) |
| 51 | 262 | Strauss (2006) p. 64 & 69 (#609) |
|  | 311 | Horsfall (2009) p. 27 (#693) |
|  | 312 | Horsfall (2009) p. 27 (#693) |
|  | 314 | Horsfall (2009) p. 27 (#693) |
| 52 | 266 | Vanderplasschen (2004) p.915 (#624) |
| 53 | 279 | Anon. (2006) p.17 (#709) |
| 54 | 280 | Voluntary Organisations Disability Group (2011) p.11 (#711) |
|  | 302 | Cornes et al. (2014) p.141 (#823) |
| 55 | 285 | Rosenheck et al. (2001) p.706 (#793) |
| 56 | 286 | Shelter (2007) p.4 (#794) |
| 57 | 288 | Milaney (2012) p.5 (#704) |
| 58 | 290 | Milaney (2012) p.5 (#704) |
| 59 | 295 | Kipping (2011) p. 232 (#638) |
| 60 | 297 | Kim et al. (2004) p.114 (#701) |
| 61 | 299 | Cameron et al. (2006) (#735) |
| 62 | 248 | Bodenheimer (2002) p. 1775 (#579 and #578) |
|  | 301 | Cornes et al. (2014) p.140 (#823) |
| 63 | 303 | McNeill & Weaver (2010) p.6 (#824) |
| 64 | 304 | McNeill & Weaver (2010) p.7 (#824) |
|  | 318 | Draine (2007) p. 1580 (#619) |
| 65 | 306 | McNeill & Weaver (2010) p.64-70 (#824) |
| 66 | 313 | Horsfall (2009) p. 27 (#693) |
| 67 | 323 | Karoll (2010) p. 270 (#589) |
| 68 | 328 | Karoll (2010) p. 271 (#589) |
| 69 | 332 | Kavanagh (2013) p. 411 (#845) |
| 70 | 343 | Jaffe (2012) (#316) |
| 71 | 344 | Le Boutillier et al. (2011) p.1474 (#359) |
| 72 | 345 | Bateman & Krawitz (2013) p.176 (#919) ; Fonagy et al. (2012) p.37 (#921) |
| 73 | 347 | Fonagy et al. (2012) p.34 (#921) ; Fonagy & Bateman (2006) p.424 (#923) |
| 74 | 348 | Fonagy et al. (2012) p.37 (#921) ; Bateman & Fonagy (2012) p.68 (#922) ; Fonagy & Bateman (2007) p.93 (#925) |
| 75 | 394 | Jordan (2012) p. 730 |
